# Artificial Gold Enzymes Using a Genetically Encoded Thiophenol‐Based Noble‐Metal‐Binding Ligand

**DOI:** 10.1002/anie.202421912

**Published:** 2024-12-17

**Authors:** Mathijs J. Veen, Friso S. Aalbers, Henriëtte J. Rozeboom, Andy‐Mark W. H. Thunnissen, Daniel F. Sauer, Gerard Roelfes

**Affiliations:** ^1^ Stratingh Institute for Chemistry University of Groningen 9747 AG Groningen, the Netherlands; ^2^ Groningen Biomolecular Sciences and Biotechnology Institute University of Groningen 9747 AG Groningen, the Netherlands

**Keywords:** Designer enzymes, Gold catalysis, Thiophenol, Non-canonical amino acids, Biocatalysis

## Abstract

Incorporating noble metals in artificial metalloenzymes (ArMs) is challenging due to the lack of suitable soft coordinating ligands among natural amino acids. We present a new class of ArMs featuring a genetically encoded noble‐metal‐binding site based on a non‐canonical thiophenol‐based amino acid, 4‐mercaptophenylalanine (*p*SHF), incorporated in the transcriptional regulator LmrR through stop codon suppression. We demonstrate that *p*SHF is an excellent ligand for noble metals in their low oxidation states. The corresponding gold(I) enzyme was characterised by mass spectrometry, UV/Vis spectroscopy and X‐ray crystallography and successfully catalysed hydroamination reactions of 2‐ethynyl anilines with turnover numbers over 50. Interestingly, two equivalents of gold(I) per protein dimer proved to be required for activity. Up to 98 % regioselectivity in the hydroamination of an ethynylphenylurea substrate was observed, yielding the corresponding phenyl‐dihydroquinazolinone product, consistent with a π‐activation mechanism by single gold centres. The ArM was optimized by site saturation mutagenesis using an on‐bead screening protocol. This resulted in a single mutant that showed higher activity for one class of substrates. This work brings the power of noble‐metal catalysis into the realm of enzyme engineering and establishes thiophenols as alternative ligands for noble metals, providing new opportunities in coordination chemistry and catalysis.

## Graphical abstract

A new class of artificial metalloenzymes containing genetically encoded noble‐metal‐binding sites featuring a non‐canonical thiophenol‐based amino acid, which serves as an excellent soft ligand for binding various noble metals, was developed. The corresponding gold(I) enzyme was characterised, confirming gold binding to the thiophenol, and successfully applied in catalytic hydroamination reactions.

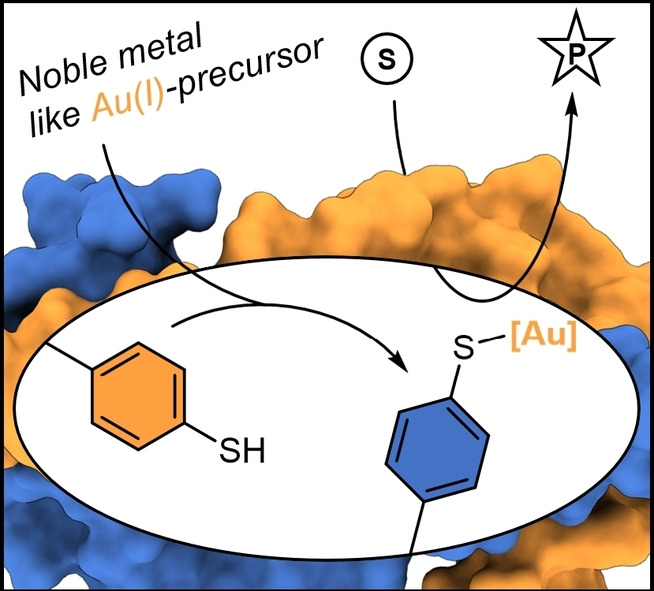

## Introduction

**Figure 1:**
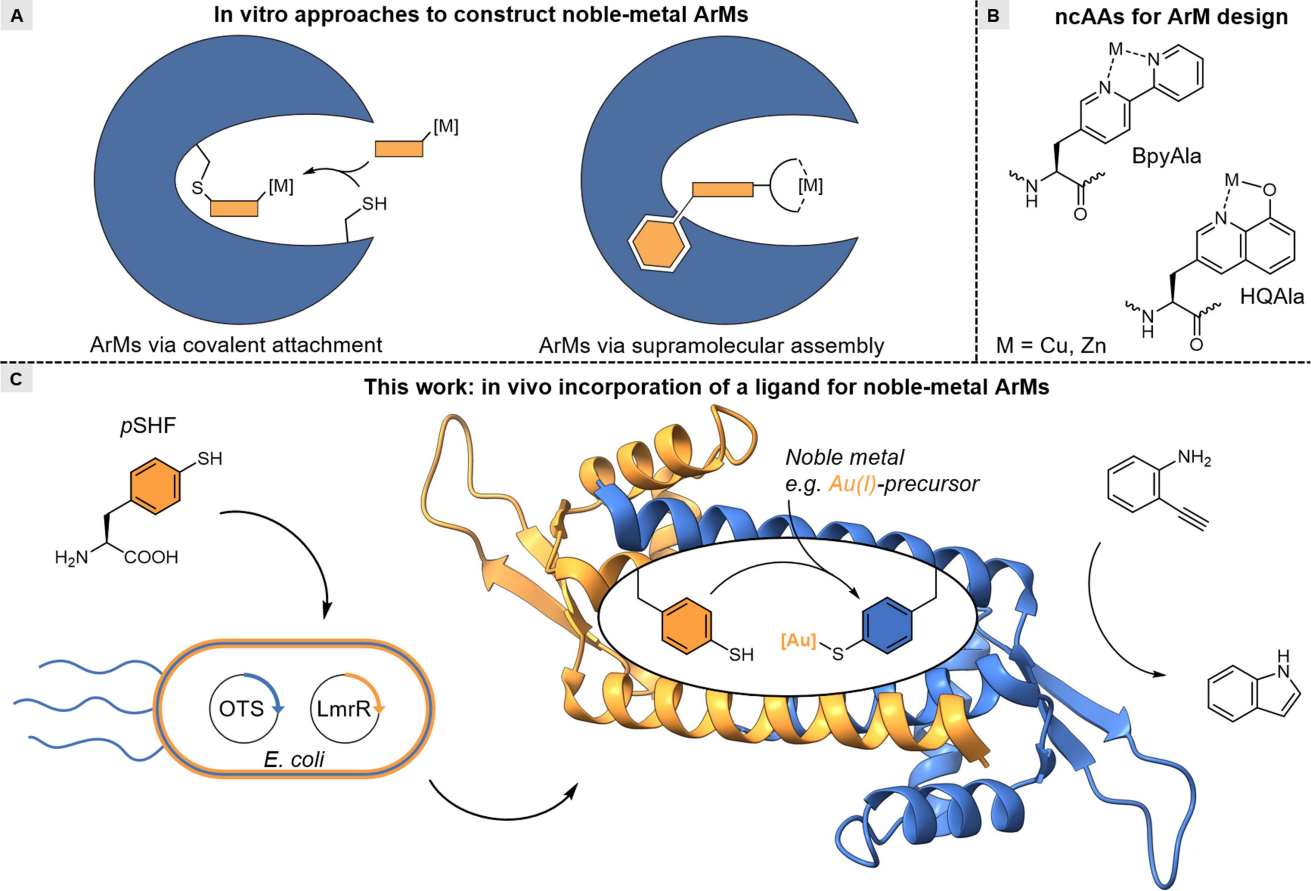
Context of in vivo incorporation of *p*SHF for ArM design. A) Currently most common approaches to anchor noble‐metal complexes to protein scaffolds for ArMs include covalent modification (left) and supramolecular assembly (right). B) BpyAla and HQAla expanded the metal‐binding amino acid repertoire for ArM design. C) In vivo incorporation of a noble‐metal‐binding ligand gives convenient access to noble‐metal ArMs, which catalyse hydroamination reactions (this work).

Metalloenzymes catalyse many of the challenging chemical transformations that are essential for life with unmatched activities and selectivities.[^1^, ^2^] To enable these reactions, they primarily rely on 3d‐transition metals. In contrast, in synthetic chemistry, noble‐metal catalysis is omnipresent due to its unique and diverse reaction scope, which includes reaction classes that are not available in nature, such as cross‐coupling reactions,^[3]^ olefin metathesis,^[4]^ and alkyne activation.[^5^, ^6^] The desire to expand the catalytic repertoire of biocatalysis beyond the transformations known in nature has given rise to artificial metalloenzymes (ArMs), which are hybrids comprising abiological metal ions or complexes embedded in natural or designed protein scaffolds.^[7]^ Many of these ArMs feature noble metals in the active site to bring their unique chemistry into the realm of biocatalysis. Noble metals, especially in their low oxidation states, generally require soft polarisable ligands, such as *N*‐heterocyclic carbenes (NHCs) and phosphines, which are not available in nature's canon of amino acids. Hence, dative anchoring of noble metals in proteins is virtually non‐existent.[^8^, ^9^] Therefore, ArMs featuring noble metals are mainly constructed from pre‐prepared complexes that are incorporated into proteins via supramolecular anchoring or bioconjugation (Figure 1A).[^10^, ^11^, ^12^, ^13^, ^14^, ^15^, ^16^, ^17^, ^18^, ^19^, ^20^, ^21^] This involves the additional chemical syntheses of complexes, is limited to distinct protein scaffolds, and often requires an in vitro ligand anchoring step that in certain cases requires additional purification steps.

Genetic code expansion strategies such as stop codon suppression (SCS) have emerged as a powerful tool to introduce non‐canonical amino acids (ncAAs) into proteins.^[22]^ Such ncAAs can be employed as metal‐binding ligands, which removes the need for an in vitro ligand anchoring step.[^23^, ^24^, ^25^] Using SCS, 3‐(2,2′‐bipyridin‐5‐yl)‐alanine (BpyAla) and 8‐hydroxyquinolin‐3‐yl alanine (HQAla) have been applied to create artificial metalloenzymes (Figure 1B). However, these amino acids are better equipped to recruit 3d‐transition metals for ArMs.[^26^, ^27^, ^28^, ^29^] Genetically encoding common ligands for noble‐metal complexes, like NHCs or phosphines, has proven difficult to achieve due to their structure and their instability under aerobic conditions in a cellular environment. To date, there are no reports of ArMs containing genetically encoded noble‐metal‐binding ligands. There is an example where a masked phosphine ligand, a phosphine‐borane adduct, was genetically incorporated.^[30]^ However, the associated post‐translational deprotection step required long reaction times, and metal‐binding was only achieved with one metal complex. No catalysis was reported for this artificial metalloprotein.

In addition to NHCs[^31^, ^32^] and phosphines,^[33]^ thiolates represent an important class of soft donor ligands for noble‐metal‐binding.[^34^, ^35^] In synthetic transition metal complexes, thiolates mainly exist as part of multidentate ligands in combination with phosphines or amines, or as sterically demanding thiophenolate ligands.[^36^, ^37^] Homoleptic transition metal complexes using thiolates are rare as complexation is often accompanied by the formation of polymers and aggregates, leading to insoluble clusters.^[38]^ As a result, creating well‐defined catalytic centres involving donating thiophenolic ligands is challenging. However, in nature, the aliphatic thiol sidechain of the canonical amino acid cysteine is often used for the binding of transition metals in metalloproteins without forming aggregates.[^39^, ^40^] Spatial separation of the thiolate ligands allows for the formation of defined 3d‐transition metal complexes. To apply this to noble metals, we aimed to construct a metal‐binding site using a thiophenolic ligand, which is a more polarisable residue than cysteine, and utilise it to bind softer transition metals, like the noble metals. Here, we report a new class of artificial metalloenzymes featuring a genetically encoded thiophenol for binding noble metals and the application of this design approach by the creation of an artificial gold enzyme that shows efficient catalysis of abiological hydroamination reactions (Figure 1C).

## Results and Discussion

### Design considerations

We selected 4‐mercaptophenylalanine (*p*SHF), a thiophenolic amino acid reminiscent of tyrosine as the noble‐metal‐binding amino acid. *p*SHF was synthesised in two steps from the commercially available *N*‐Boc‐4‐iodo‐l‐phenylalanine with an overall yield of 70 % (Supporting Information section 1).[^41^, ^42^] Incorporation of *p*SHF into proteins via amber stop codon suppression requires an appropriate orthogonal translation system (OTS) that consists of an aminoacyl‐tRNA synthetase (aaRS) that can orthogonally load *p*SHF onto a tRNA with the anti‐codon to the amber stop codon. Even though the structure of *p*SHF closely resembles the arsenal of ncAAs that have been incorporated by OTS systems based on the tyrosyl tRNA/synthetase pair of *Methanococcus jannaschii*, no dedicated OTS has been reported for *p*SHF, to date.[^22^, ^43^] Therefore, we screened a library of 11 candidate OTSs to probe for promiscuous activity for the incorporation of *p*SHF, using superfolder green fluorescent protein (sfGFP) with the stop codon substitution Y151TAG (TAG=amber stop codon) as the reporter (Supporting Information section 2).^[44]^ From this screening, the pEVOL_pAzF OTS,^[45]^ initially developed for *para*‐azido‐phenylalanine (*p*AzF), was found to have promiscuous activity towards *p*SHF.

Next, we investigated the incorporation of *p*SHF into Lactococcal multidrug resistance regulator (LmrR), which has been demonstrated to be a highly versatile protein scaffold for artificial enzyme design.^[46]^ We selected position V15 for incorporation, since this places *p*SHF inside the hydrophobic pocket at the dimer interface.[^30^, ^47^] Initial attempts to incorporate *p*SHF into LmrR resulted in low protein yields (<5 mg/L) and undesirable protein modifications. Oxidation as well as methylation of the thiol were observed by mass spectrometry (Supporting Information section 3).

The protein yield was substantially improved by switching to pEVOL_pAzF_RS.2.t1,^[48]^ which is an improved variant of the pEVOL_pAzF OTS. However, the improvement was associated with substantial misincorporation of the canonical amino acids phenylalanine and tyrosine. This problem was negated by using minimal medium with vitamins (MMV) as the expression medium, which contains lower concentrations of canonical amino acids and tRNAs during expression compared to rich media. Optimisation of the expression temperature, as well as the addition of the reductant tris(2‐carboxyethyl)phosphine (TCEP) during protein production and purification, minimised modifications of the thiol group. Using the optimised protocol, after affinity chromatography purification, LmrR_V15*p*SHF was obtained in yields of 75–145 mg/L cell culture. The protocol is robust and was used for all different LmrR_*p*SHF variants. We confirmed the purity of LmrR_V15*p*SHF with HRMS and SDS‐page (Supporting Information section 4). In addition, purified LmrR_V15*p*SHF was crystallised and its structure was determined at 2.35 Å resolution, revealing well‐resolved electron density for the two *p*SHF residues in the protein homodimer with no apparent chemical modifications of the thiol groups (Supporting Information section 5).

### Binding of noble metals

**Scheme 1:**
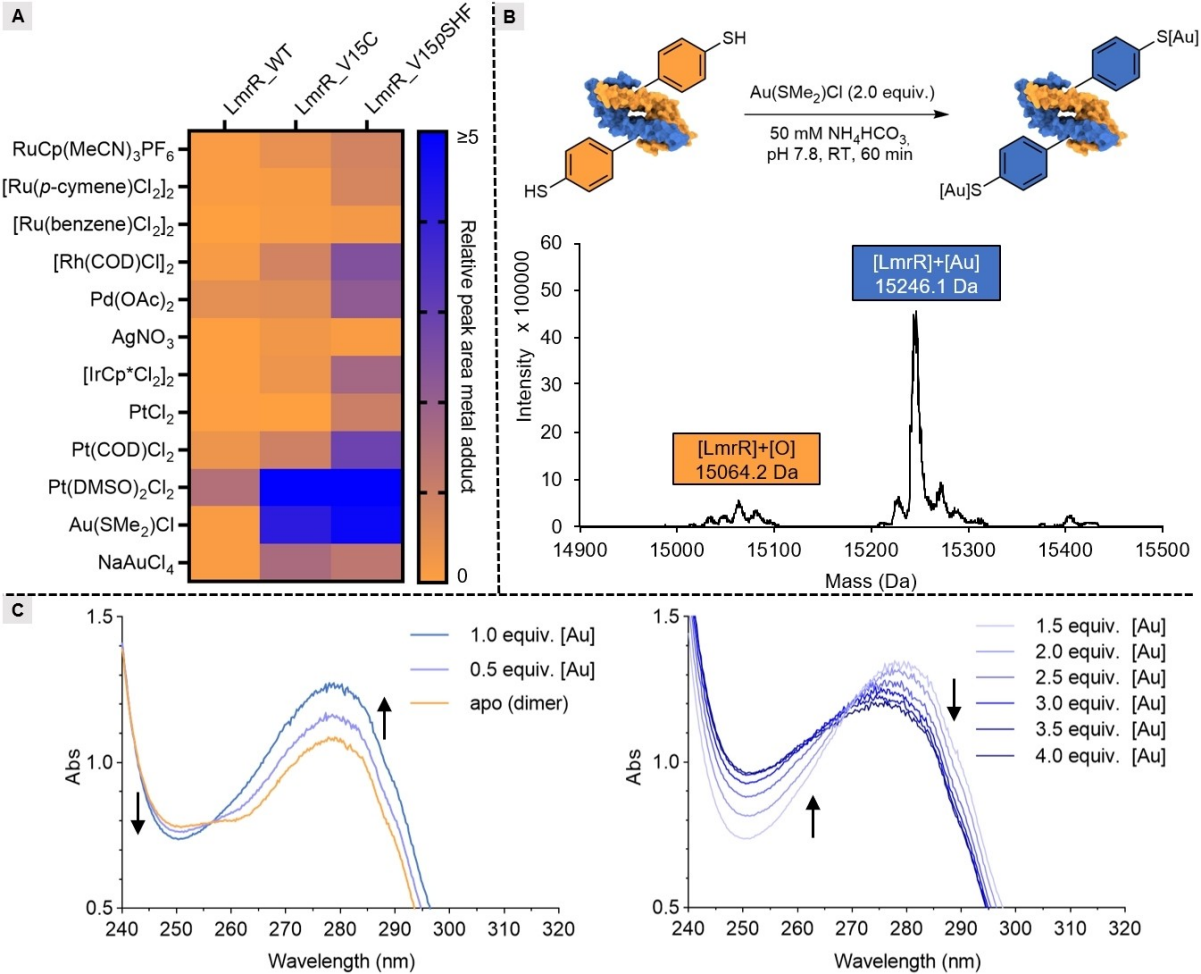
Evaluation of thiophenol containing protein LmrR_V15*p*SHF in noble‐metal‐binding. A) Overview of metal adduct formation of LmrR_WT, LmrR_V15 C and LmrR_V15*p*SHF using LC–MS. The areas of the peaks containing LmrR with metal are divided by the areas of the peaks containing LmrR without metal. The metal precursors (2.0 equiv. of metal ion) were incubated with LmrR variants (1.0 equiv. dimer) in NH_4_HCO_3_ buffer. B) A representative example for one of the metal‐binding experiments, where Au(SMe_2_)Cl is supplemented to LmrR_V15*p*SHF yielding LmrR_V15*p*SHF−Au. For clarity the thiophenols are depicted pointing out of the protein scaffold, while in reality they are inside the protein, as shown in Figure 2. C) On the left, the UV/Vis spectrum of 0, 0.5, and 1.0 equiv. of Au(SMe_2_)Cl titrated to LmrR_V15*p*SHF (dimer). On the right, the UV/Vis spectrum of 1.5–4.0 equiv. of Au(SMe_2_)Cl titrated to LmrR_V15*p*SHF (dimer).

Having our noble‐metal‐ligand‐containing protein in hand, we investigated its affinity towards noble metals. The noble‐metal‐binding properties were assessed by electrospray ionisation mass spectrometry (ESI‐MS). We conducted binding experiments by supplying a variety of noble‐metal ions and complexes to the apo protein and measured adduct formation either by direct injection into the mass spectrometer or by first separating metals that are weakly interacting with the protein using liquid chromatography (LC) (Scheme 1A, Supporting Information section 6). LmrR_V15*p*SHF is envisioned to have two specific metal‐binding sites per dimer. As a control, the same experiments were performed with the LmrR protein without *p*SHF (LmrR_WT) and the corresponding cysteine variant (LmrR_V15C).

The artificial metalloproteins were readily formed in situ by incubating the metal precursors with the protein scaffold at ambient temperature. To our delight, LmrR_V15*p*SHF binds a variety of noble metals, albeit with varying degrees of efficiency and sometimes accompanied by oxidation. Metal complexes with ligands like cyclopentadiene (Cp), pentamethylcyclopentadiene (Cp*), or 1,5‐cyclooctadiene (COD) predominantly retained that ligand after binding to the proteins. Precursors like Au(SMe_2_)Cl and Pd(OAc)_2_, upon combination with the protein, showed the formation of a single species in ESI‐MS, corresponding to the (LmrR_V15*p*SHF+metal) mass. In most cases, there was substantially more of the metal adduct detectable for LmrR_V15*p*SHF compared to LmrR_WT and LmrR_V15C, showing the potency of *p*SHF in binding noble‐metal ions or complexes. No binding was observed when using metal precursors with high oxidation states, like RuI_3_, RuCl_3_, and RhCl_3_, which is in accordance with the Hard and Soft Acids and Bases (HSAB) principle.^[49]^ Based on the rapid and clean conversion observed under MS‐conditions for LmrR_V15*p*SHF with Au(SMe_2_)Cl (LmrR_V15*p*SHF−Au from here on) (Scheme 1B), we investigated this metalloprotein further. Since LmrR_WT does not show significant binding of Au(SMe_2_)Cl in LC–MS, we hypothesised that *p*SHF plays a key role in the binding. To confirm this hypothesis, Au(SMe_2_)Cl was titrated to LmrR_WT and LmrR_V15*p*SHF and monitored by UV/Vis spectroscopy. Indeed, no changes were observed in the UV/Vis spectrum when Au(SMe_2_)Cl was titrated to LmrR_WT. In contrast, titrating Au(SMe_2_)Cl into LmrR_V15*p*SHF solutions resulted in notable changes in the absorption spectra (Scheme 1C). Up to a 1 : 1 [Au]:LmrR_V15*p*SHF (dimer) ratio, an increase of the absorption band at 280 nm, and a decrease in absorption at 250 nm with an isosbestic point at 257 nm was detected. Further addition of Au(SMe_2_)Cl until two equivalents with respect to dimer resulted in a distinctive change of the absorption spectra, which suggests that the second gold ion interacts differently with the protein scaffold. Adding more gold precursor gives rise to additional small changes in the UV/Vis, tentatively due to non‐specific interactions of the excess gold with the protein. When gold is titrated to thiophenol, the absorption peaks disappear at higher gold concentrations, which indicates that the formation of thiophenol‐Au gives rise to insoluble complexes (Supporting Information section 7).[^50^, ^51^] Aggregate formation of LmrR_V15*p*SHF−Au is avoided because the two thiophenolic residues are spatially separated in the protein dimer. In addition, the folding of LmrR_V15*p*SHF appears to be intact upon gold‐binding, since no changes in CD‐spectroscopy are observed after the addition of Au(SMe_2_)Cl to LmrR_V15*p*SHF (Supporting Information section 8).

**Figure 2:**
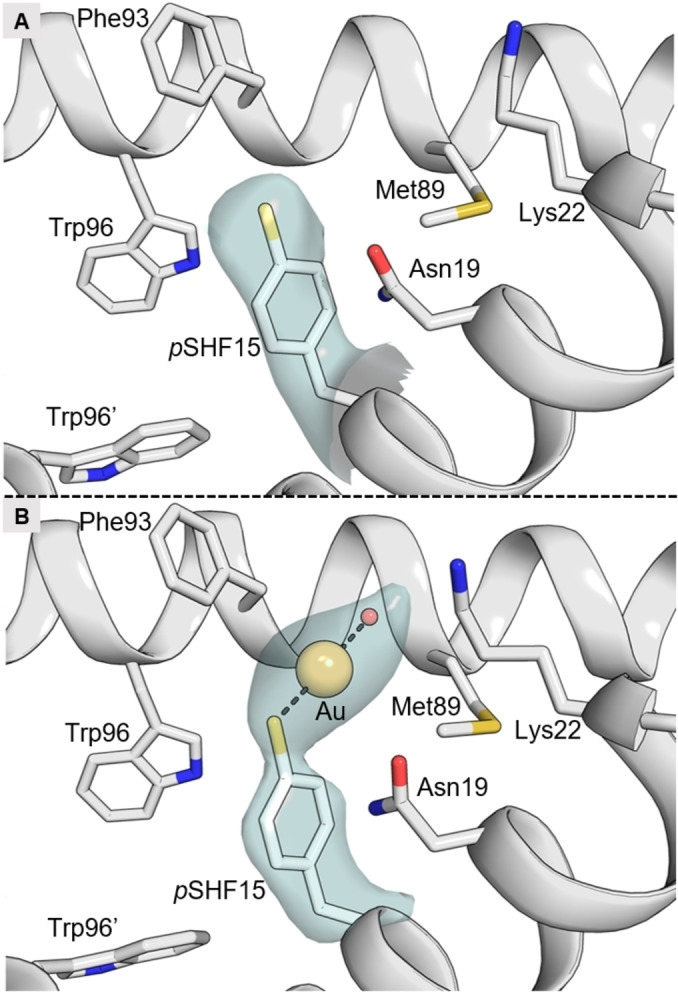
Crystal structures of apo and Au‐bound LmrR_V15*p*SHF. A) Close‐up view of apo LmrR_V15*p*SHF (PDB: 9G51), showing one of the two *p*SHF15 residues and surrounding amino acids as sticks. The cyan surface represents the Fo−Fc omit Fourier density of *p*SHF15 contoured at 2.5σ. B) Similar close‐up view of Au‐bound LmrR_V15*p*SHF (PDB: 9G52). The Fo−Fc omit Fourier density (cyan) of *p*SHF15 and the gold ion (together with a coordinating water or hydroxide ion) was contoured at 3σ.

After establishing the incorporation and position of the *p*SHF residues in LmrR using X‐ray crystallography (Figure 2A), we aimed to establish the binding mode of the gold ions in LmrR_V15*p*SHF−Au. An X‐ray crystal structure at 2.50 Å resolution was obtained via co‐crystallisation with KAuCN_2_, which is more water‐soluble than Au(SMe_2_)Cl and has been used extensively for heavy atom phasing in protein X‐ray crystallography.^[52]^ The crystal structure and crystallographic Fourier maps revealed the presence of one gold ion, coordinating one of the two *p*SHF residues in the LmrR_V15*p*SHF dimer (Figure 2B, Supporting Information section 9). The gold atom is associated with a strong peak in the anomalous difference Fourier map, unambiguously establishing its identity and position in the crystal structure. In addition to the sulphur of the *p*SHF residue, a hydroxyl ion or water molecule appears to serve as a coordinating ligand of the gold ion, establishing a linear two‐coordination arrangement that is typical for Au(I).[^53^, ^54^] The gold atom is surrounded by the side chains of Asn19, Lys22, Phe93, and Met89 at a minimal distance of 4–5 Å, establishing a second coordination sphere and restricting the solvent accessibility of the gold. No clear anomalous difference peak is present near the other *p*SHF residue, and the electron density of this residue appears distorted and weak compared to its counterpart in the dimer, indicative of substantial conformational disorder. Although it is possible that gold is also bound to this second *p*SHF residue, contributing to the observed disorder, the X‐ray data is insufficient to determine this conclusively.

### Gold‐catalysed hydroamination

The thiophenolic ligand allows us to have a defined noble‐metal centre in the protein scaffold. To probe if the protein environment can enable catalytic activity, we evaluated LmrR_V15*p*SHF in combination with various metal precursors in an intramolecular hydroamination reaction of 2‐ethynylaniline (**1 a**) to form indole (**2 a**). These reactions are catalysed by a variety of transition metal catalysts.[^55^, ^56^, ^57^, ^58^, ^59^, ^60^] We probed Ru, Pt, Rh, and Au‐precursors in combination with LmrR_V15*p*SHF, using a stoichiometry of one metal ion per *p*SHF (so two metal ions per dimer) as catalyst (Supporting Information section 10). To our delight, both Au(SMe_2_)Cl and NaAuCl_4_, in combination with LmrR_V15*p*SHF, exhibited substantial reactivity, while the precursors without the protein scaffold only showed trace activity. In case of Au(III)‐binding, substantial oxidative damage of the protein was observed, which we hypothesised to result from Au(III) being reduced in situ to Au(I) by oxidising the protein. Therefore, we decided to continue with Au(SMe_2_)Cl as the metal precursor for ArM assembly. After reaction optimisation, LmrR_V15*p*SHF−Au gave rise to >50 turnover numbers (TONs) (Table 1, Supporting Information section 11), which is comparable to artificial gold‐enzymes that are constructed with NHC‐ligands by supramolecular assembly.[^12^, ^15^, ^61^] At least 2‐fold lower TON values were obtained using LmrR variants with cysteine, tyrosine, or other metal‐binding ncAAs in the pocket (Supporting Information section 12). The comparably low activity of the cysteine‐ligated gold centre is notable. It has been shown in engineered ferritin‐based systems that cysteine‐ligated gold centres are active in the catalysis of intramolecular hydrolactonisation, a reaction related to the intramolecular hydroamination studied here.^[62]^ However, in that case the cysteine is proposed to serve as a bridging ligand between two gold centres, of which one is catalytically active. In case of LmrR_V15C, the ESI‐MS results show that one gold is bound per monomer and, hence, in this case the cysteine acts as terminal ligand. The addition of a water‐soluble phosphine (TCEP) completely inactivated the catalyst. This is most likely due to the strong binding of the phosphine to the catalytic gold centre, thus making it unavailable for catalysis. Intriguingly, when using only one equivalent of gold per LmrR_V15*p*SHF dimer, no product was obtained, showing that a >1 : 1 [Au]:LmrR_V15*p*SHF (dimer) stoichiometry is necessary to obtain catalytic activity (Supporting Information section 13).

**Table 1:** Hydroamination reaction catalysed by different LmrR variants.^[a]^

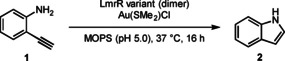			
Entry	LmrR variant	Au equiv.	TON
1	LmrR_V15*p*SHF	2.0 equiv. (20 μM)	56±12
2	LmrR_V15*p*SHF	1.0 equiv. (10 μM)	0±0
3	LmrR_V15*p*SHF	–	0±0
4	No protein	2.0 equiv. (20 μM)	4±0
5	LmrR_WT	2.0 equiv. (20 μM)	21±4
6	LmrR_V15C	2.0 equiv. (20 μM)	9±1
7	LmrR_V15Y	2.0 equiv. (20 μM)	17±3

[a] Reaction conditions: 1 mM substrate **1 a**, 1 mol % ArM, 20 mM MOPS, 150 mM NaCl, pH 5.0, 2.6 %v/v MeCN, 37 °C, 850 rpm, 16 h. Yields and conversions are obtained by GC‐FID using mesitylene as internal standard. Results are obtained as an average of two experiments, errors are given as ±(standard deviation).

To gain further insight into the relation between [Au]‐stoichiometry and activity, we investigated a reaction in which urea substrate (**3**) converts either into a 6‐*exo*‐*dig* cyclisation product (**4**), through π‐activation by a single gold centre, or a 5‐*endo*‐*dig* cyclisation product (**5**), through σ,π‐activation by two gold centres.[^15^, ^63^] Using free Au(SMe_2_)Cl or LmrR_WT−Au gave rise to the formation of both products, with a preference for product **4**. While increasing the concentration of gold led to higher yields of **4** and **5**, the selectivity remained the same.

**Scheme 2:**
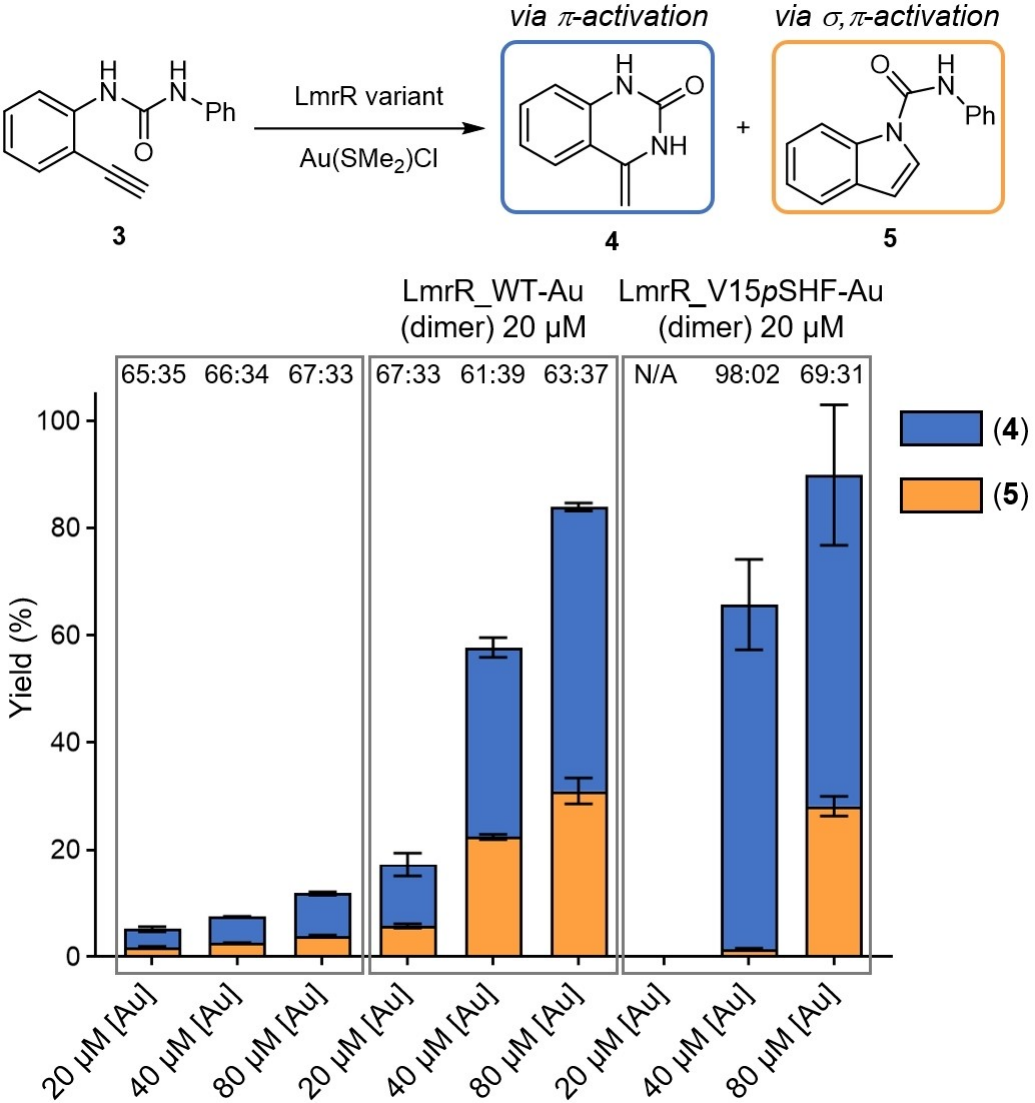
Gold ArM catalysed intramolecular hydroamination of substrate **3**. Single π‐activation or dual σ,π‐activation of the substrate will afford a 6‐*exo‐dig* cyclisation product (**4**) or a 5‐*endo‐dig* cyclisation product (**5**), respectively. Yield and selectivity profiles with different scaffolds and varying equivalents of Au(SMe_2_)Cl. Results are obtained as an average of two experiments, and errors are given as ±(standard deviation).

In contrast, no product was formed when using a 1 : 1 [Au]:LmrR_V15*p*SHF (dimer) ratio, similar to what was observed in the hydroamination of substrate **1 a**. Notably, using a 2 : 1 [Au]:LmrR_V15*p*SHF (dimer) ratio gave a good activity with near complete selectivity towards product **4**. Using more gold, so a ratio of >2 : 1 [Au]:LmrR_V15*p*SHF (dimer), an increasing amount of product **5** was observed, eventually approaching the same product ratio as obtained with LmrR_WT−Au (Scheme 2, Supporting Information section 14).

The fact that at a 2 : 1 [Au]:LmrR_V15*p*SHF (dimer) ratio almost exclusively product **4** is obtained, points to a π‐activation mechanism by a single gold centre. Therefore, even though the LmrR_V15*p*SHF dimer binds two gold ions, there is no synergistic interaction between them, most likely due to spatial separation of the gold ions. Only when more Au(SMe_2_)Cl is added, considerable amounts of product **5** are formed. The loss of selectivity can be attributed to unspecific gold‐binding or protein‐free gold after the *p*SHF sites are saturated, this could then assist in the catalysis, making a σ,π‐activation mechanism by two gold centres possible.

Intriguing is also the absence of any catalytic activity when only one equivalent of Au(SMe_2_)Cl per LmrR_V15*p*SHF dimer is used, both in this reaction and in the hydroamination of substrate **1 a**. The reason for this remains unclear at the moment. It could suggest that the two gold‐binding sites are not equivalent, in agreement with the UV/Vis results and the X‐ray crystal structure. Alternatively, this may suggest that in the case of an 1 : 1 [Au]:LmrR_V15*p*SHF (dimer) stoichiometry, the gold atom is coordinatively saturated and therefore unable to engage in catalysis. Further research will be needed to understand the origin of this effect.

### Probing the second coordination sphere

**Figure 3:**
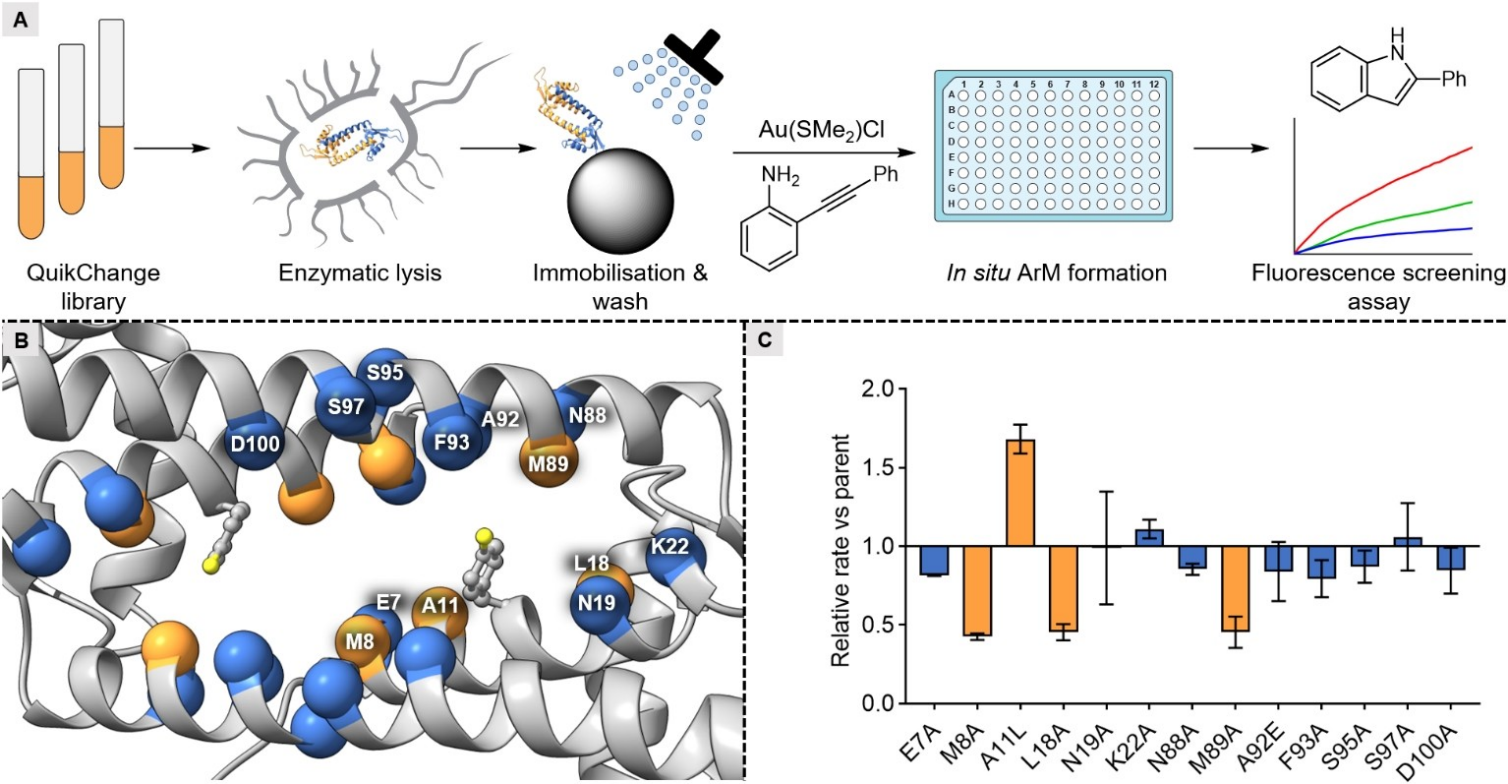
Mutagenesis strategy to probe the second coordination sphere of the gold centre. A) Protocol to generate and screen LmrR_*p*SHF−Au variants. B) Various positions in and around the hydrophobic pocket were targeted for an alanine scanning. Selected positions are indicated by orange and blue spheres. For clarity, only one of the monomers is labelled. C) Depiction of relative rates of the alanine variants compared to LmrR_V15*p*SHF (parent). The variants showing a significant change in reaction rate are labelled orange, whereas variants showing similar rates to the parent are labelled blue. Results are obtained as an average of two experiments, errors are given as ±(standard deviation).

**Scheme 3:**
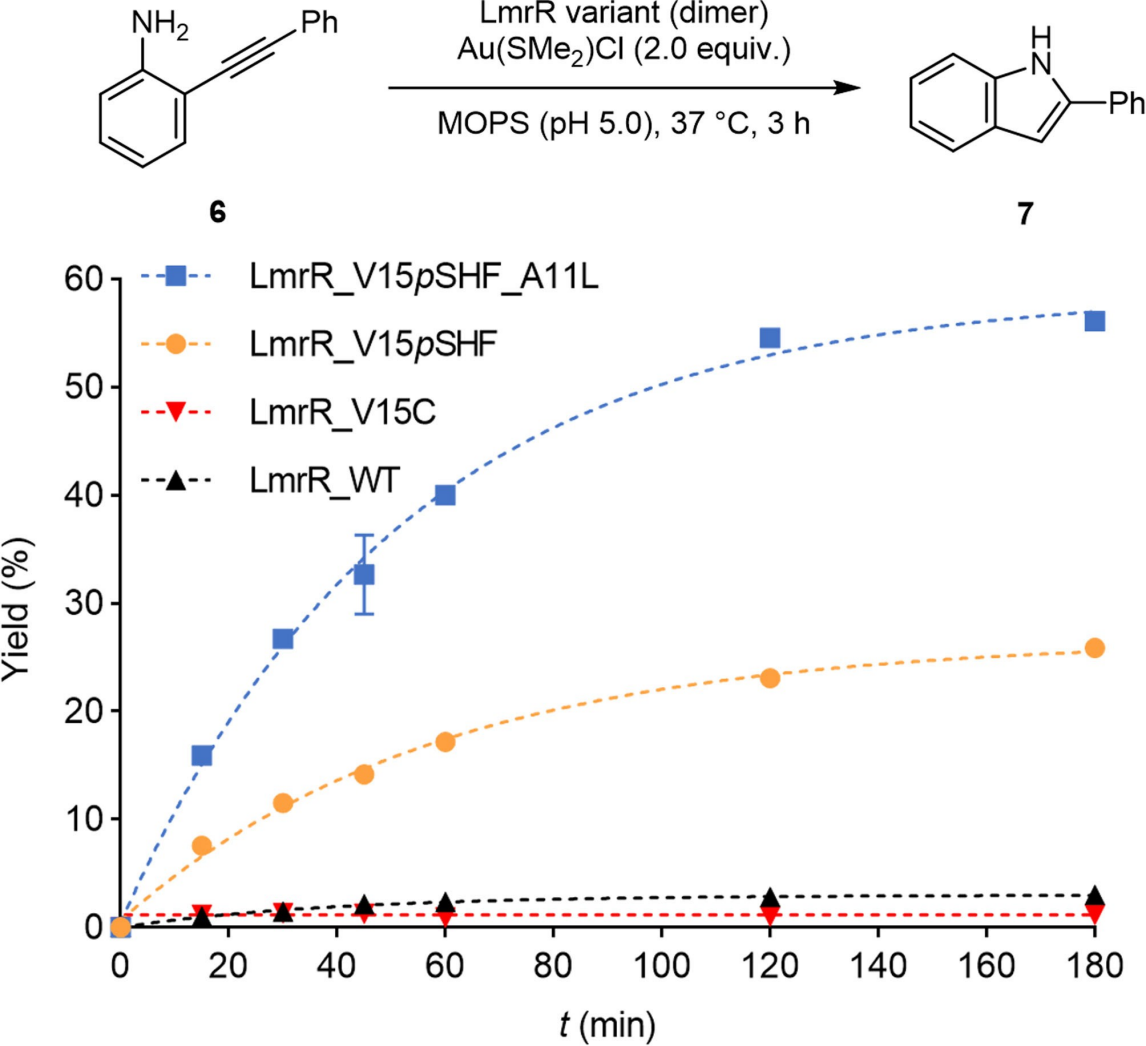
Time course of gold ArMs catalysing the intramolecular hydroamination of substrate **6 a**. Relative activity of LmrR variants, displayed by yield of **7 a** over time. Reaction conditions: 200 μM substrate **6 a**, 5 μM LmrR variant, 10 μM Au(SMe_2_)Cl, 20 mM MOPS, 150 mM NaCl, pH 5.0, 2.6 %v/v MeCN, 37 °C, 3 h. Results are obtained as an average of two experiments, and errors are given as ±(standard deviation).

An attractive feature of the genetic incorporation of a metal‐binding ligand is the close proximity of surrounding amino acids that can interact with the metal as a second coordination sphere. The effect of the environment around the gold centre was investigated by varying the position of the *p*SHF within the hydrophobic pore and mutagenesis of the residues close to the gold centre. To facilitate increased throughput methodologies for enzyme screening, we selected 2‐(phenylethynyl)aniline (**6 a**) as a substrate. The intramolecular hydroamination of the internal alkyne gives rise to 2‐phenylindole (**7 a**), which is fluorescent and thus allows for experiments to be performed in a plate reader (Figure 3A, Supporting Information section 15). In addition, the observed background reactivity was drastically reduced compared to substrate **1 a**, as is evident from the results of LmrR_WT−Au (Scheme 3). Using cell‐free extracts for the screening of reactivity proved not feasible due to the presence of inhibiting cellular components. Therefore, we used the C‐terminally fused Strep‐tag II to bind the LmrR variants to Strep‐Tactin resin after cell lysis. This enables facile removal of other cellular components by washing and, upon sequential addition of Au(SMe_2_)Cl and substrate **6 a**, the catalytic reaction was performed using the immobilised LmrR_*p*SHF−Au variants.

We tested eight different LmrR variants with the *p*SHF placed at various positions (Supporting Information section 16). Of these, LmrR_A11*p*SHF and LmrR_V15*p*SHF showed notable activity. The V15*p*SHF variant was selected for further optimisation, since it showed the highest activity. Next, an alanine scanning was performed to determine which residues in the second coordination sphere have an impact on the catalytic activity (Figure 3B). This entails substituting several residues around the *p*SHF by alanine or, if the original residue was alanine, by another amino acid. Four positions in the proximity of the *p*SHF showed significantly changed activity upon mutation (Figure 3C, Supporting Information section 17). Of these, we selected two positions for site‐saturation mutagenesis: A11, which showed a faster reaction upon substitution to leucine, and M89, which in the crystal structure is in close proximity of the *p*SHF‐bound gold centre and gave rise to lower activity when replaced with alanine. In case of site‐saturation at position A11, improved activities were observed for several variants in the plate screen. A comparison of the isolated and purified hits confirmed the A11L variant to be the most active. Subsequently, using LmrR_V15*p*SHF_A11L as the template, position M89 was site‐saturated and the variants were screened. All substitutions at this position were deleterious, which implies that the methionine at position 89 is crucial for the catalytic activity (Supporting Information section 18). A time course experiment on isolated and purified LmrR_V15*p*SHF_A11L−Au showed a substantial improvement in rate and yield (Scheme 3). The kinetic studies revealed a ~2.3‐fold improvement in catalytic efficiency (*k*_cat_/*K*_M_) compared to LmrR_V15*p*SHF−Au (Supporting Information section 19).

The mutagenesis studies demonstrate the evolvability of these designed scaffolds for noble‐metal catalysis. This is especially vital for the development of catalysts for synthetically relevant transformations that can be evolved for enantioselectivity.

**Scheme 4:**
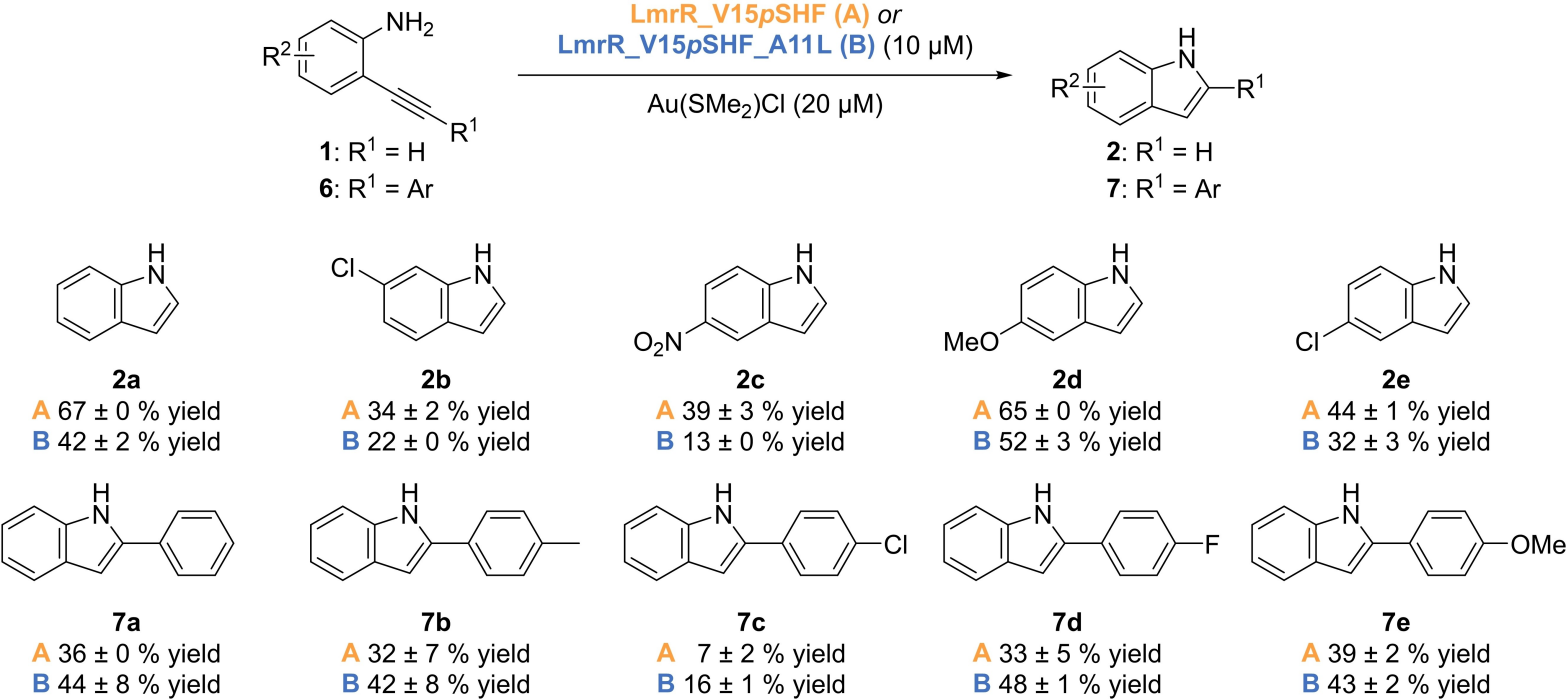
Substrate scope of the hydroamination of various 2‐ethynyl anilines with LmrR_V15*p*SHF−Au and LmrR_V15*p*SHF_A11L−Au. Reaction conditions: 1 mM substrate, 10 μM LmrR variant, 20 μM Au(SMe_2_)Cl, 20 mM MOPS, 150 mM NaCl, pH 5.0, 2.6 %v/v MeCN, 37 °C, 16 h. Yields are obtained by SFC using 2‐phenylquinoline as internal standard. Results are obtained as an average of two experiments, and errors are given as ±(standard deviation).

Finally, we evaluated the substrate scope for catalytic conversion by LmrR_V15*p*SHF−Au and LmrR_V15*p*SHF_A11L−Au (Scheme 4). In general, moderate to good yields of product were obtained. Somewhat lower activities were observed when electron‐withdrawing substituents were present. A clear trend is observed for the two enzyme variants: using the terminal alkyne substrates **1**, better product yields were obtained with the parent gold enzyme. In contrast, with the internal alkyne substrates **6**, improved yields of the corresponding products **7 a**–**e** were obtained with the mutant LmrR_V15*p*SHF_A11L−Au.

## Conclusion

Herein we have shown that by using stop codon suppression, we can create genetically encoded noble‐metal‐binding sites in proteins based on *p*SHF, a ncAA containing a thiophenol side chain, at any desired location. This gives convenient access to artificial noble‐metal enzymes for selective new‐to‐nature transformations, as demonstrated for the artificial gold enzyme in this study. Moreover, thiophenol ligands are under‐represented in transition metal catalysis due to their propensity to form insoluble polymeric structures. As shown here, this can be circumvented by the incorporation into a protein scaffold, which keeps them in a well‐defined, structurally tunable microenvironment and spatially separated from other thiophenols. Thus, this work establishes thiophenol as an attractive alternative for NHC and phosphine ligands in noble‐metal catalysis. Our current efforts are focused on the application of this noble‐metal enzyme design approach for the development of enantioselective noble‐metal biocatalysis of synthetically relevant reactions.

## Conflict of Interests

The authors declare no conflict of interest.

1

## Supporting information

As a service to our authors and readers, this journal provides supporting information supplied by the authors. Such materials are peer reviewed and may be re‐organized for online delivery, but are not copy‐edited or typeset. Technical support issues arising from supporting information (other than missing files) should be addressed to the authors.

Supporting Information

## Data Availability

The data that support the findings of this study are available in the supplementary material of this article.
