# Supplementary material for: Artificial Gold Enzymes Using a Genetically Encoded Thiophenol‐Based Noble‐Metal‐Binding Ligand
Source: Angew Chem Int Ed Engl. 2024 Dec 17;64(12):e202421912. doi: 10.1002/anie.202421912 (PMC11914936; doi:10.1002/anie.202421912)
Supplement: Supplementary file 1 — Supporting Information [file ANIE-64-e202421912-s001.pdf]

Angewandte Chemie
Eine Zeitschrift der Gesellschaft Deutscher Chemiker

Supporting Information

Artificial Gold Enzymes Using a Genetically Encoded Thiophenol-Based Noble-Metal-Binding Ligand

M. J. Veen, F. S. Aalbers, H. J. Rozeboom, A.-M. W. H. Thunnissen, D. F. Sauer, G. Roelfes**

Supporting Information

Artificial Gold Enzymes using a Genetically Encoded Thiophenol-Based Noble-Metal-Binding Ligand

Mathijs J. Veen,[a] Friso S. Aalbers,[a] Henriëtte J. Rozeboom,[b] Andy-Mark W. H. Thunnissen,[b] Daniel F. Sauer,*[a] and Gerard Roelfes*[a]

[a] Stratingh Institute for Chemistry, University of Groningen, 9747 AG, Groningen, The Netherlands. Email: J.G.Roelfes@rug.nl, danielsa@miltenyi.com

[b] Groningen Biomolecular Sciences and Biotechnology Institute, University of Groningen, 9747 AG, Groningen, The Netherlands

Table of contents

Supplementary information ''Artificial Gold Enzymes using a Genetically Encoded Thiophenol-Based Noble-Metal-Binding Ligand''

SI.1 *p*SHF synthesis
SI.2 OTS screening for *p*SHF incorporation
SI.3 Optimisation of expression and purification LmrR_V15*p*SHF
SI.4 Characterisation LmrR_V15*p*SHF
SI.5 Structural analysis apo LmrR_V15*p*SHF by X-ray crystallography
SI.6 Metal-binding screening using mass spectrometry
SI.7 [Au]-titration using UV-vis spectroscopy (LmrR_WT and thiophenol)
SI.8 CD-spectroscopy of LmrR_V15*p*SHF-Au
SI.9 Structural analysis of Au-bound LmrR_V15*p*SHF
SI.10 Metal precursor screening for biocatalytic hydroamination
SI.11 Reaction optimisation
SI.12 Control experiments relevance of *p*SHF residue
SI.13 Control experiments catalyst components
SI.14 Regioselectivity of the hydroaminase
SI.15 Validation of screening protocol LmrR_*p*SHF variants
SI.16 Positional screening of the *p*SHF residue
SI.17 Alanine scanning
SI.18 Site-saturation mutagenesis
SI.19 Comparison kinetic properties of improved variant and parent

General procedures and additional data

SI.20 General considerations
SI.21 sfGFP assay procedures
SI.22 Molecular biology procedures (protein expression and mutagenesis)
SI.23 Library preparation and screening procedures
SI.24 Mass spectrometry procedures
SI.25 HR-mass spectra of purified proteins and Au-bound proteins
SI.26 X-ray crystallography procedures
SI.27 Spectroscopy procedures (UV-vis, CD)
SI.28 Catalysis and kinetic experiments procedures
SI.29 Synthetic chemistry (synthesis and NMR)
SI.30 SFC and GC-FID chromatograms and calibration curves
SI.31 References supporting information

SI.1 *p*SHF synthesis

Incorporation of ncAAs in proteins generally involves a synthetic approach to prepare the target amino acid. Our target amino acid can be synthesised in two high-yielding steps from a commercially available substrate (Scheme S1).[1,2] The synthesis can be upscaled to decagram scale, which is desirable to minimise the synthetic effort for the production of LmrR_*p*SHF variants. The final product is isolated as a mixture of its monomer and dimer form. Due to the oxidation sensitivity of the thiophenolic residue, varying amounts of monomer/dimer ratios were obtained in repetitive syntheses. Upon reduction of the dimer towards the monomer, it becomes challenging to separate the amino acid from the reducing agent and formed salts. Therefore, a mixture of reductant and *p*SHF was shortly incubated prior to addition to the cell culture for LmrR_*p*SHF expression.

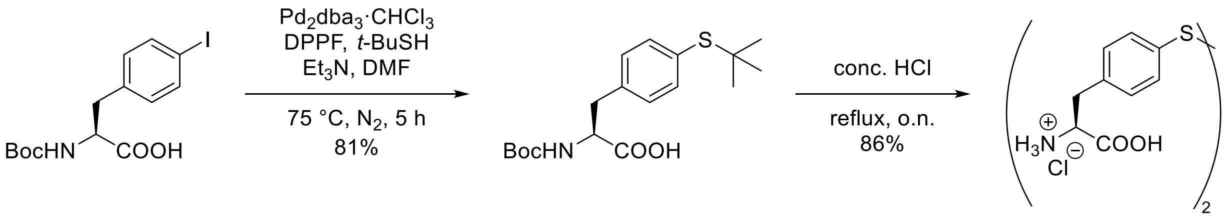

Scheme S1. Two-step synthesis towards *p*SHF.

SI.2 OTS screening for *p*SHF incorporation

Since 2001, when stop codon suppression was introduced, over 200 OTS systems have been developed to incorporate a large variety of different ncAAs. The development of these OTS systems is a laborious endeavour that generally involves creating a large library of variants and carrying out multiple rounds of positive and negative selections. Therefore, we opted for a method to screen existing OTSs for promiscuous incorporation of *p*SHF. We selected a set of OTSs based on structural similarity of the amino acids for which the OTSs were originally designed and whether the OTSs had known promiscuous activity. The OTSs were screened with a sfGFP assay. A stop codon was introduced at the position that codes for Y151 in the sfGFP gene, and the plasmid was combined with various OTS plasmids in *E. coli* BL21(DE3) cells. If the stop codon is suppressed by the OTS, a fluorescent signal appears due to sfGFP expression. Using this screening we identified basal incorporation of *p*SHF by pEVOL_pAzF, an OTS that was originally designed for *para*-azidophenylalanine (*p*AzF) (Figure S1). This is clear upon comparing the increase in relative fluorescence units (RFU) to the negative control (NC), in which no ncAA is added. *p*SHF is isolated as a mixture of monomer and dimer. Therefore we probed the effect of dithiothreitol (DTT) on the expression levels of sfGFP_Y151*p*SHF. We found that both the reduction of the synthesised *p*SHF prior to cell addition and supplementing fresh DTT to the cell culture had a beneficial effect on sfGFP production (Figure S2).

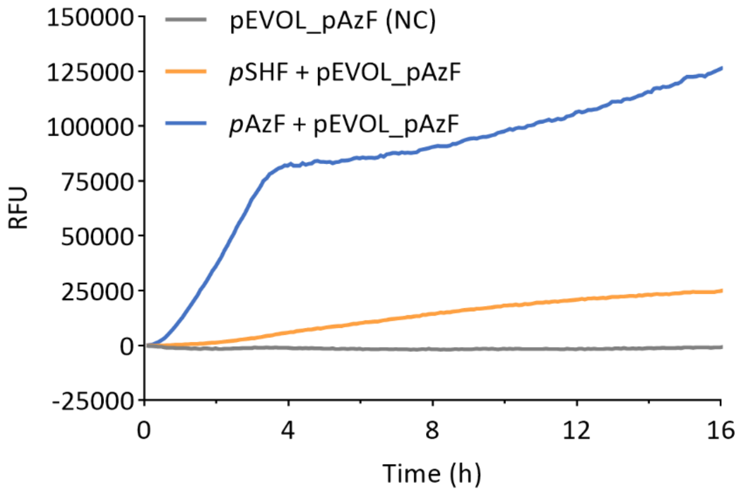

Figure S1. Fluorescence intensity spectra of the OTS screening using a sfGFP reporter.

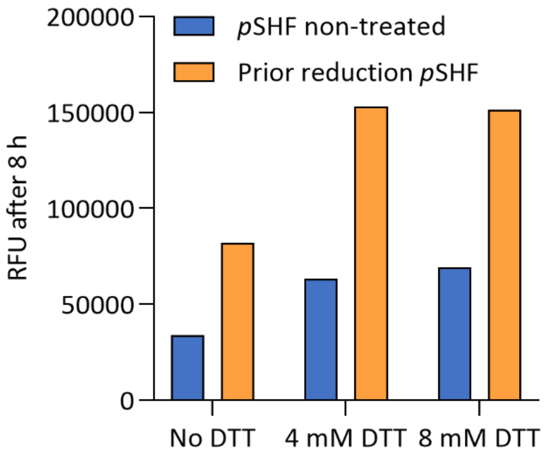

Figure S2. Effect of reducing *p*SHF-dimer prior to addition and adding reductant to media culture.

SI.3 Optimisation of expression and purification LmrR_V15*p*SHF

Starting point LmrR_V15pSHF

Initial attempts to produce and purify LmrR_V15*p*SHF were largely analogue to previously described literature for the incorporation of *p*AzF using *Escherichia coli* (*E. coli*).[3] The *p*SHF amino acid was reduced by 90 min of shaking 200 mM *p*SHF with 400 mM DTT at 37 °C. *E. coli* BL21(DE3) cells with plasmids pEVOL_pAzF and pET17b_LmrR_V15TAG were used for protein expression at 37 °C. Purification was performed according to the literature, but 1 mM EDTA was used as protease inhibitor and the lysed cells were not incubated with DNAse. This resulted in 2 mg/L protein yield and a large degree of undesirable modifications on the protein (Figure S3).

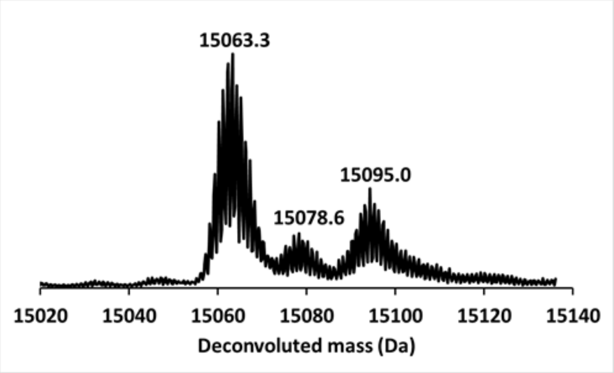

Figure S3. Deconvoluted mass spectrum of LmrR_V15*p*SHF (M_{calc} = 15048.6 Da) using pEVOL_pAzF as OTS using *E. coli*.

Expression yield optimisation of LmrR_V15pSHF

To increase the protein yield of LmrR_V15*p*SHF, we first looked for derivatives of pEVOL_pAzF that could incorporate *p*SHF more efficiently. As a result we found that pEVOL_pAzF_RS.2.t1 was also able to incorporate *p*SHF, but was giving higher protein yields. However, this came at a cost of misincorporation of canonical amino acids and in some cases significant modification on the protein (Figure S4A).

Then we attempted to change the host organism for protein expression to *Vibrio natriegens* (*V. natriegens*), an organism that is reported to have high potential to be used in overexpression of proteins.[4,5] Even though, this resulted in increased protein yields, the degree of modification was higher than in *E. coli* (Figure S4B). Therefore, from then on we continued with protein expression using *E. coli.*

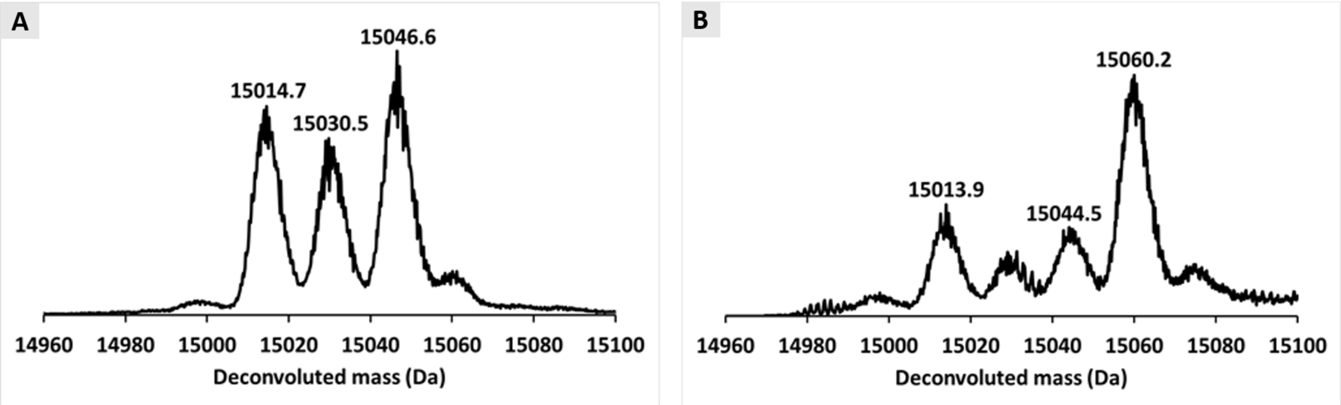

Figure S4. Deconvoluted mass spectra of LmrR_V15*p*SHF (M_{calc} = 15048.6 Da) using pEVOL_pAzF_RS.2.t1 as OTS expressed in different host organisms. **A.** Expressed in *E. coli* and **B.** Expressed in *V. natriegens*.

Identification modifications LmrR_V15pSHF

Balancing the conditions to avoid both misincorporation and modification on the protein appeared challenging. Due to the oxidation sensitivity and nucleophilicity of the thiophenol, we hypothesised the modifications were on the thiophenol. The masses in the ESI-MS were consistent with oxidation of the thiophenol mostly to its respective sulfenic- and sulfonic acid. The sulfenic acid (M_{calc} = 15064.6 Da) is a labile species and should be reduced back to the free thiol upon treatment with a potent reductant. From this point on we used tris(2-carboxyethyl)phosphine (TCEP) as the reducing agent due to a higher potency and increased stability compared to DTT. When we treated LmrR_V15*p*SHF with TCEP, ESI-MS showed that the reduction was incomplete (Figure S5). So this hinted towards a different type of modification occurring as well. Thiophenols are known to be excellent nucleophiles, so it is possible they can be alkylated in the cell. To confirm this, we treated LmrR_V15*p*SHF expressed by *V. natriegens* with TCEP and trypsin, and measured LC-MS/MS to confirm methylation on the thiophenol. The most abundant fragment containing position 15 was consistent with methylation on *p*SHF (Table S1, Figure S6).

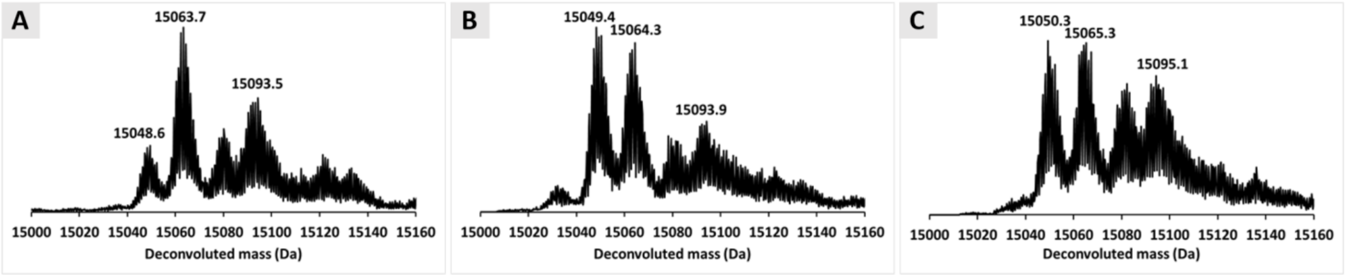

Figure S5. Deconvoluted mass spectra of LmrR_V15*p*SHF (M_{calc} = 15048.6 Da) **A.** before TCEP-treatment, **B.** after 6 h of TCEP reduction and **C.** after 26 h of TCEP reduction.

Table S1. MS/MS ion table of LmrR_V15*p*SHF (*V. natriegens* expression), most abundant mass fragment containing position 15.

Ion	Sequence	M_{obs} (Da)	M_{calc} (Da)	Error (Da)
b2	AQ	200.17400	200.10297	0.07103
b3	AQT	301.13699	301.15065	-0.01366
b4	AQTN	415.24899	415.19357	0.05542
b5	AQTN(*p*SHF*)	608.09698	608.24971	-0.15273
b5(2+)	AQTN(*p*SHF*)	305.11401	305.13241	-0.0184
b6	AQTN(*p*SHF*)I	721.18903	721.33377	-0.14474
b7	AQTN(*p*SHF*)IL	834.08197	834.41784	-0.33587
b8	AQTN(*p*SHF*)ILL	947.17700	947.50190	-0.3249
b9	AQTN(*p*SHF*)ILLN	1061.14197	1061.54483	-0.40286
b10	AQTN(*p*SHF*)ILLNV	1160.32703	1160.61324	-0.28621
b11	AQTN(*p*SHF*)ILLNVL	1273.28601	1273.69731	-0.4113
y11(2+)	KLVNLLI(*p*SHF*)NTQ	674.42499	674.88650	-0.46151
y10	KLVNLLI(*p*SHF*)NT	1220.37695	1220.70714	-0.33019
y10(2+)	KLVNLLI(*p*SHF*)NT	610.97699	610.85721	0.11978
y9	KLVNLLI(*p*SHF*)N	1119.38599	1119.65946	-0.27347
y9(2+)	KLVNLLI(*p*SHF*)N	560.36401	560.33337	0.03064
y8	KLVNLLI(*p*SHF*)	1005.38702	1005.61654	-0.22952
y8(2+)	KLVNLLI(*p*SHF*)	503.31299	503.1191	0.19389
y7	KLVNLLI	812.34900	812.56040	-0.2114
y6	KLVNLL	699.39600	699.47634	-0.08034
y5	KLVNL	586.29102	586.39227	-0.10125
y4	KLVN	473.28201	473.30821	-0.0262
y3	KLV	359.33701	359.26528	0.07173
y2	KL	260.43799	260.19687	0.24112

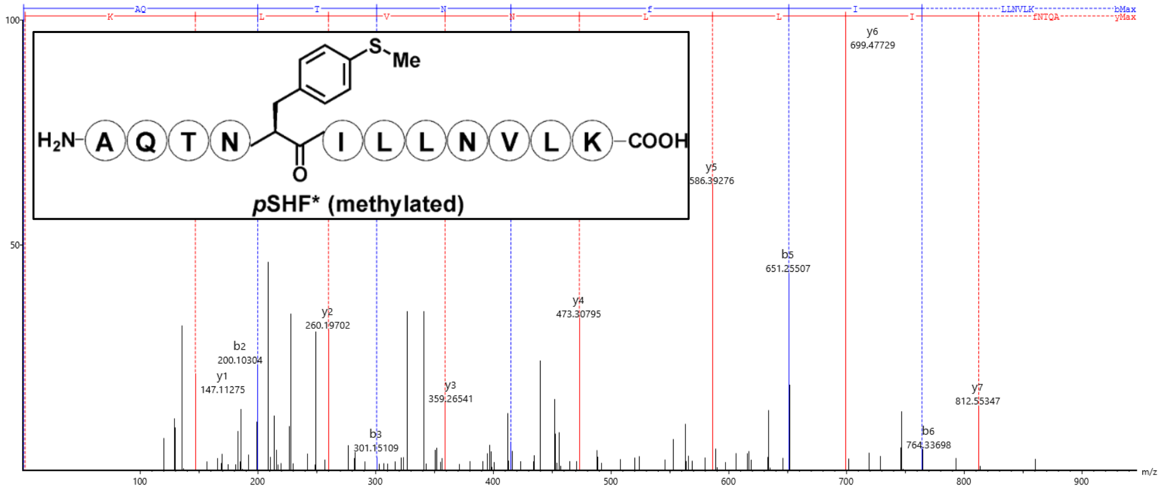

Figure S6. MS/MS spectrum of LmrR_V15*p*SHF (*V. natriegens* expression), most abundant mass fragment containing position 15. *p*SHF* represents methylated *p*SHF.

Further optimisation of LmrR_V15pSHF production

Using this information we targeted parameters during expression and purification to mitigate misincorporation, oxidation and methylation. The key parameters to obtain a higher protein purity were:

- Media: minimal medium with vitamins (MMV)
- Expression temperature: 24 °C
- Expression and purification additives: 1 mM TCEP

The deconvoluted mass spectra show that under optimised conditions a high purity of LmrR_V15*p*SHF is obtained (Figure S7).

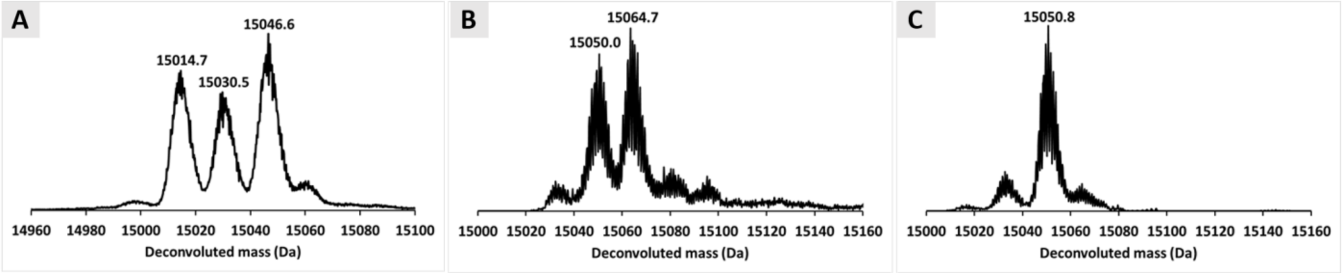

Figure S7. Deconvoluted mass spectra of LmrR_V15*p*SHF (M_{calc} = 15048.6 Da) expressed under different conditions. **A.** Expressed in LB at 30 °C. **B.** Expressed in MMV at 30 °C. **C.** Expressed in MMV at 24 °C.

SI.4 Characterisation LmrR_V15*p*SHF

The purity of LmrR_V15*p*SHF, expressed under optimised conditions, was confirmed by HRMS, SDS-page gel and trypsin digest. HRMS and SDS-page gel indicate that the desired structure was formed without any apparent modifications (Figure S8, Figure S9). During trypsin digest, free thiols are protected with iodoacetamide. The most abundant fragment containing position 15 was the protected thiol, which extrapolates from the unmodified thiol before protection (Table S2, Figure S10).

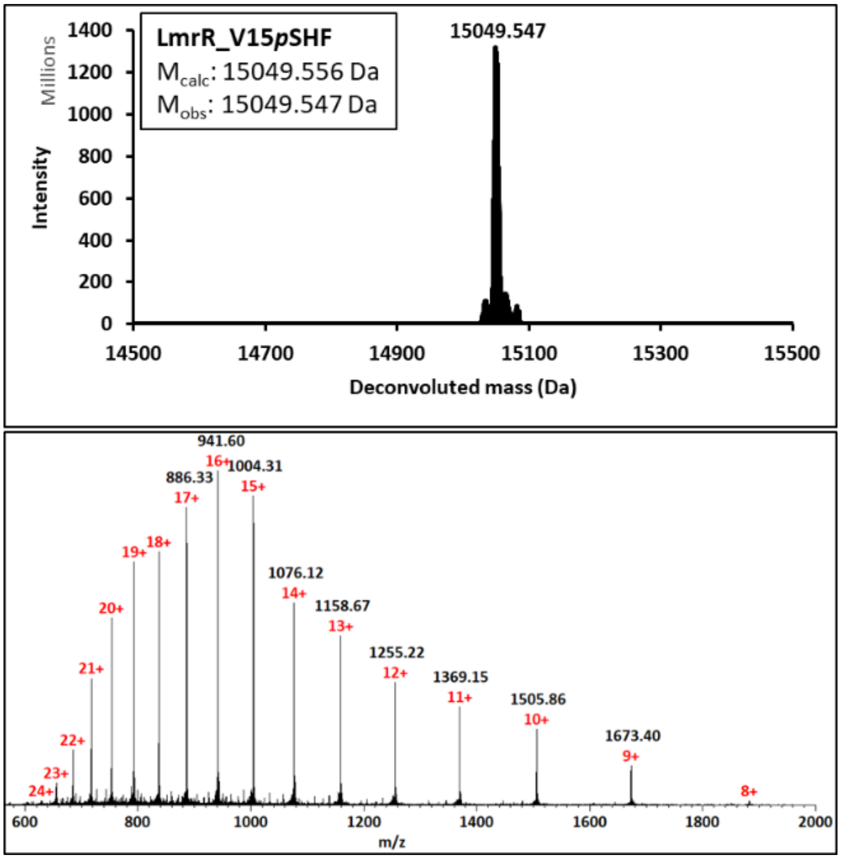

Figure S8. High resolution mass spectrum of LmrR_V15*p*SHF

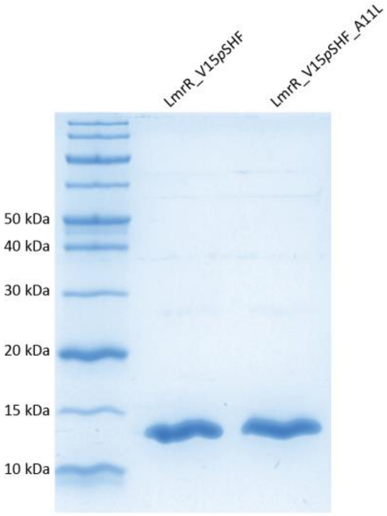

Figure S9. SDS-page gel of LmrR_V15*p*SHF and LmrR_V15*p*SHF_A11L

Table S2. MS/MS ion table of LmrR_V15*p*SHF (after optimisation), most abundant mass fragment containing position 15. *p*SHF** represents protected *p*SHF.

Ion	Sequence	M_{obs} (Da)	M_{calc} (Da)	Error (Da)
b2	AQ	200.10304	200.10297	0.00007
b3	AQT	301.15109	301.15065	0.00044
b4	AQTN	415.19479	415.19357	0.00122
b5	AQTN(pSHF**)	651.25507	651.25552	-0.00045
b6	AQTN(pSHF**)I	764.33698	764.33959	-0.00261
y7	KLVNLLI	812.55347	812.56040	-0.00693
y6	KLVNLI	699.47729	699.47634	0.00095
y5	KLVNL	586.39276	586.39227	0.00049
y4	KLVN	473.30795	473.30821	-0.00026
y3	KLV	359.26541	359.26528	0.00013
y2	KL	260.19702	260.19687	0.00015
y1	K	147.11275	147.11280	-0.00005

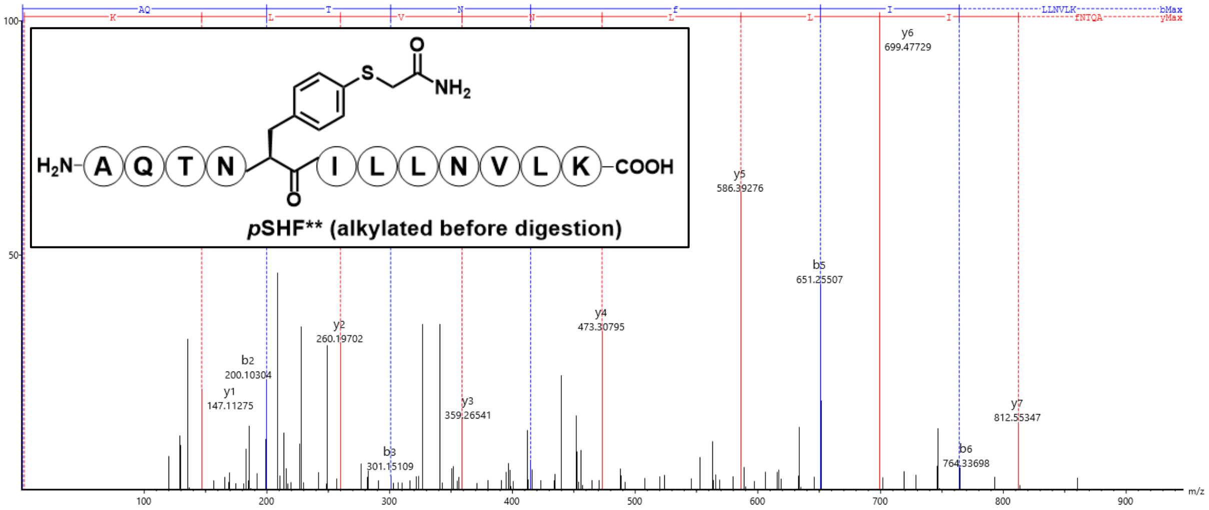

Figure S10. MS/MS spectrum of LmrR_V15*p*SHF (after optimisation), most abundant mass fragment containing position 15.

SI.5 Structural analysis of apo LmrR_V15*p*SHF by X-ray crystallography

The crystal structure of apo LmrR_V15*p*SHF exhibits a dimeric protein conformation closely resembling that of the wild-type LmrR. This indicates that the introduced *p*SHF residues do not cause destabilising interactions that affect the protein's overall structure. The electron density for the two *p*SHF residues is well-resolved, confirming their presence within the pocket at position 15 in each of the two subunits, with no apparent modifications of the thiol groups (Figure S11).

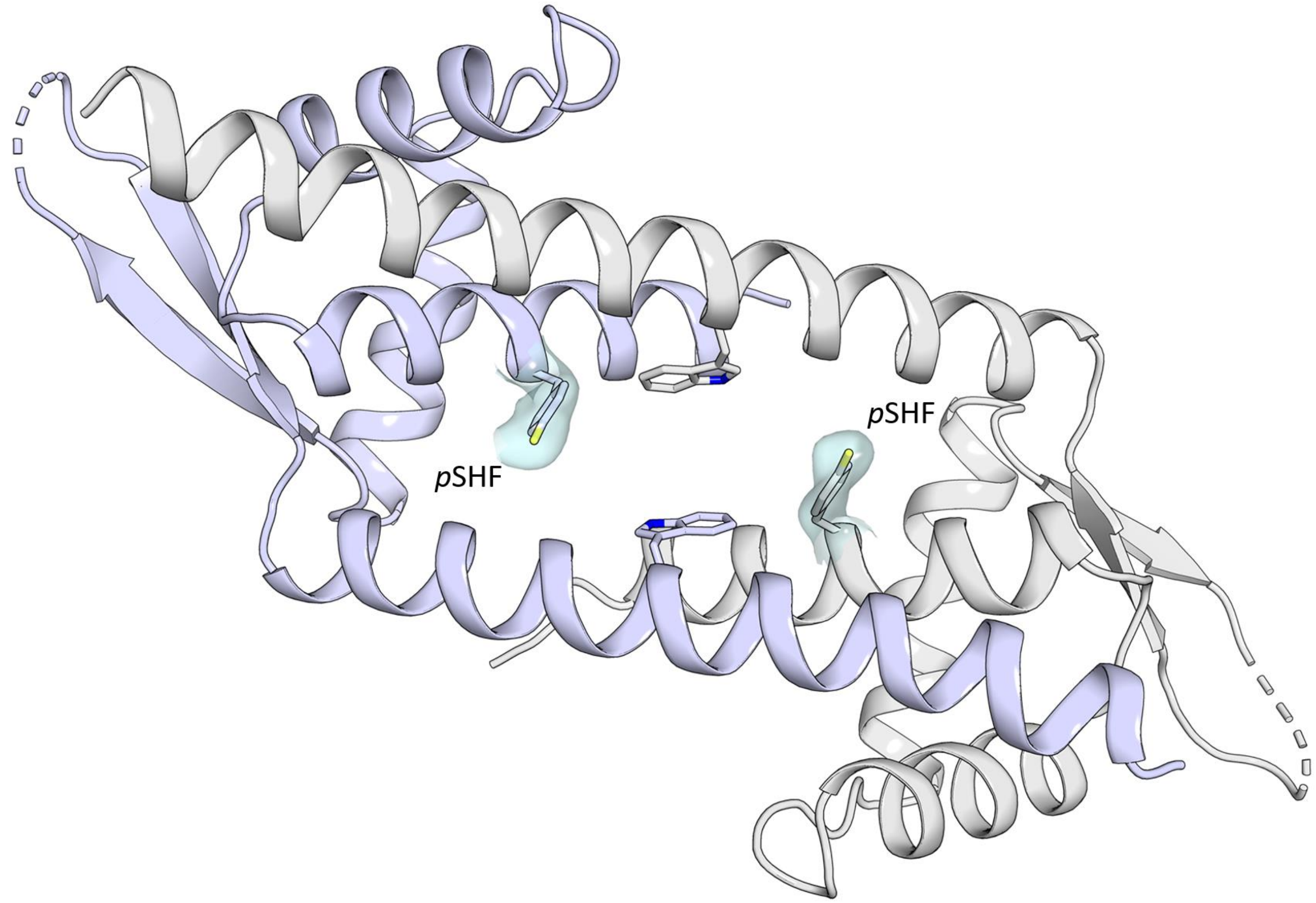

Figure S11. Crystal structure of dimeric apo LmrR_V15*p*SHF (PDB: 9G51), showing the two *p*SHF residues as sticks and their associated $2F_o$-F_c electron densities (contoured at 1.2σ) in cyan. Also shown are the two Trp96 residues in the central hydrophobic pore.

SI.6 Metal-binding screening using mass spectrometry

To probe the metal-binding properties of LmrR_V15pSHF, various noble-metal ions and complexes were incubated with the protein scaffold. The binding was evaluated as metal-adduct formation using ESI-MS. Three measurements per sample were performed: after 30-60 min (BP1) and 180-240 min (BP2), the sample was injected onto a bypass column, and after 60-180 min, the sample was injected onto a C4 column before ESI-MS analysis. LmrR_WT and LmrR_V15C were measured as controls.

The bypass measurements BP1 and BP2 avoid chromatographic separation of sample components, facilitating the observation of metal-protein interactions that would otherwise not be visible due to dissociation upon interaction with a C4 column. We implemented two separate bypass measurements to assess potential increases in metal-adduct formation over time. In addition, we evaluated metal-adduct formation by subjecting the proteins to elution through a C4 column before ESI-MS. Persistence of metal binding under these conditions would indicate the formation of more stable interactions. We hypothesise that metal-protein interactions exhibiting a higher degree of covalent character are more readily detectable via mass spectrometry.

Upon integration of the peaks in the deconvoluted mass spectra, the ratios of the peak areas representing metal-bound LmrR species to those representing apo LmrR species were determined. These ratios were used to qualitatively compare the metal-binding properties of the different LmrR species (Table S3).

Comparing LmrR_V15*p*SHF to LmrR_WT, the results show the incorporation of *p*SHF greatly impacts the metal-binding properties of the LmrR scaffold. This is especially clear when the samples are injected over the column, where metal complexes that are not bound or too weakly bound to the protein are separated. We also compared the effect of *p*SHF to cysteine. Even though LmrR_V15C shows good binding towards platinum and gold, most metals give only trace metal adduct formation. Therefore, it can be concluded that *p*SHF has potential to be used for creating artificial noble-metal-binding sites in protein scaffolds. In the deconvoluted mass spectra various species were detected, which was highly dependent on the precursor that was used.

Table S3. Metal adduct formation in ESI-MS between various transition metal precursors and LmrR variants. Number ratios are given as (peak area metal containing peaks in deconvoluted mass spectrum/peak area of apo LmrR peaks in deconvoluted mass spectrum)

	**LmrR_WT**			**LmrR_V15C**			**LmrR_V15*p*SHF**		
	BP1	BP2	Column	BP1	BP2	Column	BP1	BP2	Column
**$RuCp(MeCN)_3PF_6$**	0.15	0.14	0.11	0.45	0.43	0.45	0.78	0.74	0.87
**[Ru(*p*-cymene)Cl_2]$_2$**	0.06	0.13	0.10	0.07	0.23	0.13	0.58	0.79	0.79
**$[Ru(benzene)Cl_2]_2$**	0.05	0.05	0.00	0.19	0.18	0.12	0.06	0.52	0.20
**$RuCl_3$**							0.14	0.00	0.15
**$[Rh(COD)Cl]_2$**	0.13	0.14	0.14	0.40	0.46	0.88	6.43	9.29	2.44
**$RhCl_3$**							0.00	0.03	0.06
**$Pd(OAc)_2$**	0.16	0.24	0.52	0.30	0.43	0.57	1.60	1.87	2.17
**$AgNO_3$**	0.11	0.11	0.01	0.67	0.77	0.26	0.59	0.54	0.10
**$[IrCp^*Cl_2]_2$**	0.50	0.50	0.01	1.80	3.29	0.34	2.73	3.44	1.77
**$PtCl_2$**	0.02	0.09	0.03	0.05	0.11	0.00	0.65	1.06	1.01
**$Pt(COD)Cl_2$**	0.43	0.61	0.32	3.06	3.04	0.95	15.38	18.88	2.88
**$Pt(DMSO)_2Cl_2$**	1.44	1.54	1.48	5.81	6.65	10.20	4.84	4.91	5.34
**$O[Si(CH_3)_2CH{=}CH_2]_2Pt$**							0.00	0.01	0.06
**$Au(SMe_2)Cl$**	0.81	0.71	0.10	4.13	7.82	4.13	2.66	1.69	4.80
**$NaAuCl_4$**	0.30	0.30	0.10	0.90	1.06	1.63	0.67	0.45	1.26

LmrR_V15pSHF + RuCp(MeCN)$_3$PF$_6$ – column

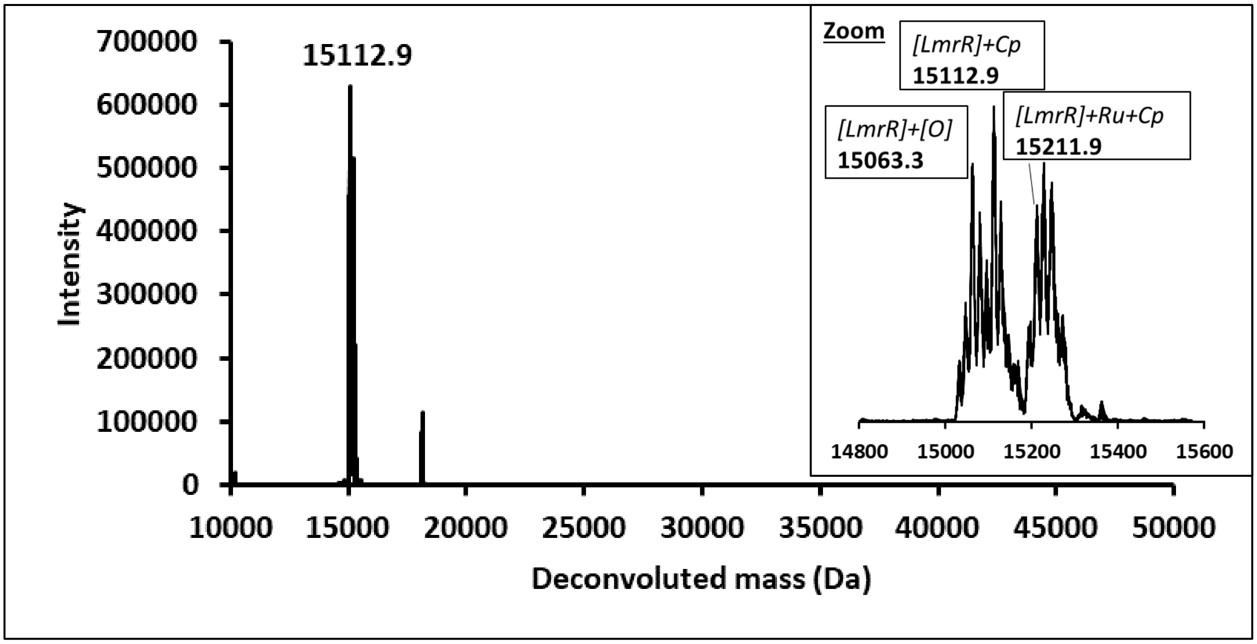

LmrR_V15pSHF + [Rh(COD)Cl]$_2$ – column

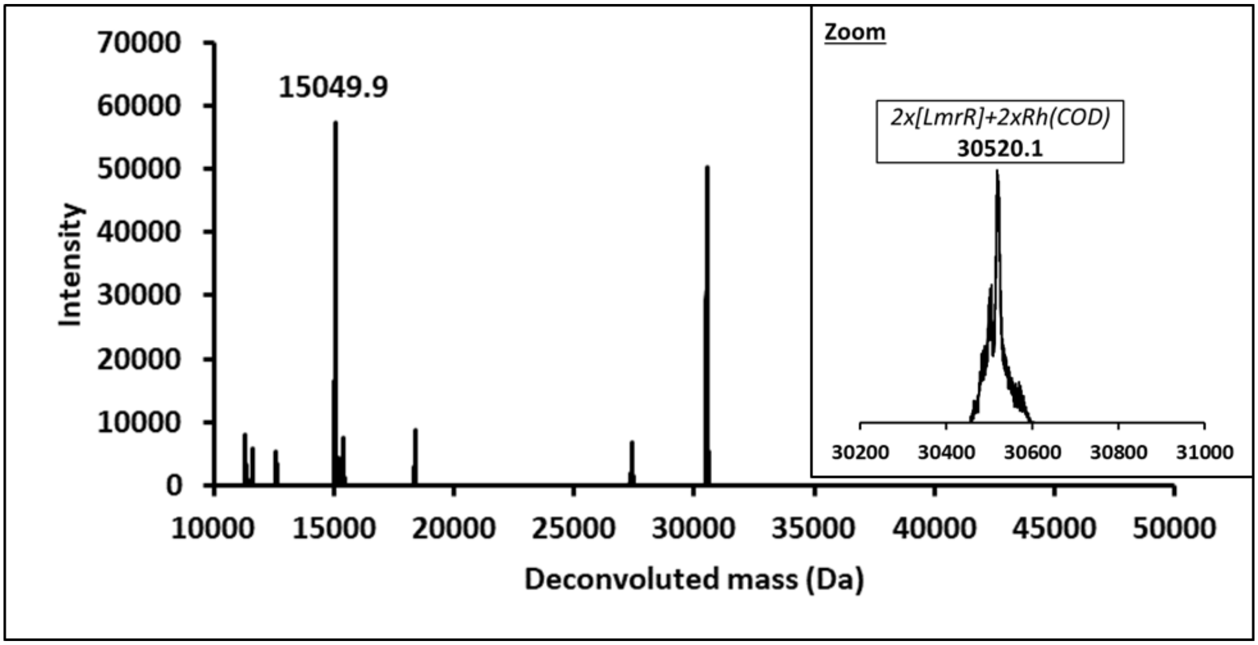

LmrR_V15pSHF + Pt(COD)Cl_2 – column

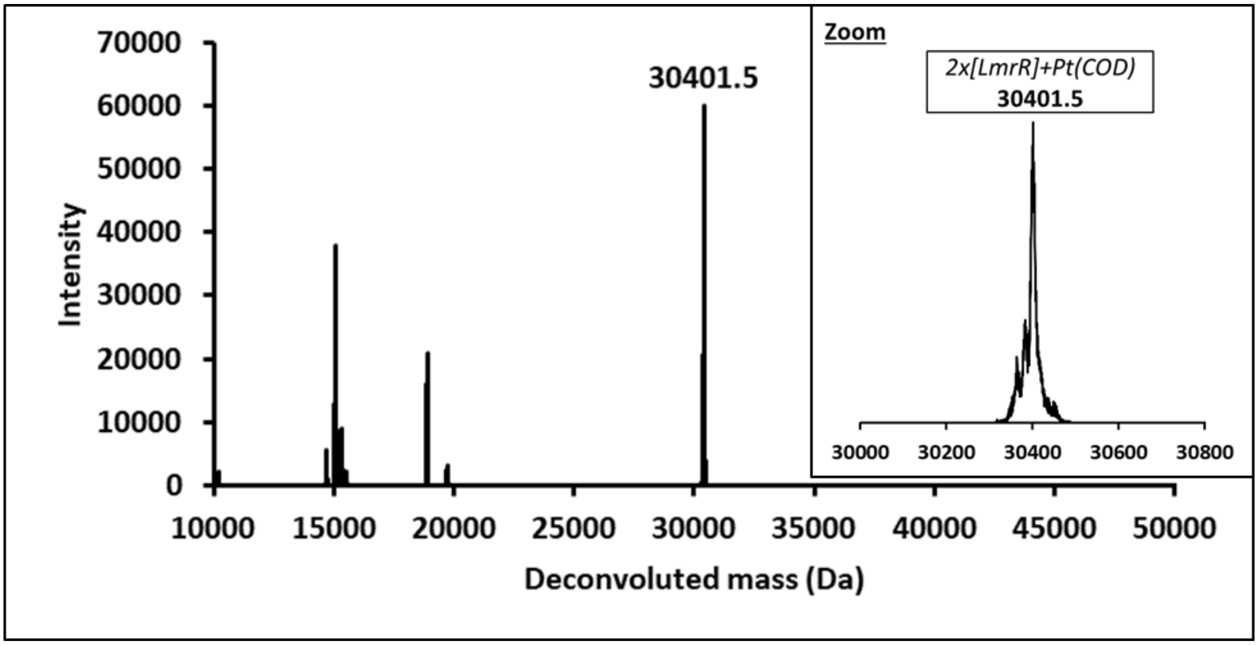

LmrR_V15pSHF + Pt(DMSO)$_2$$Cl_2$ – column

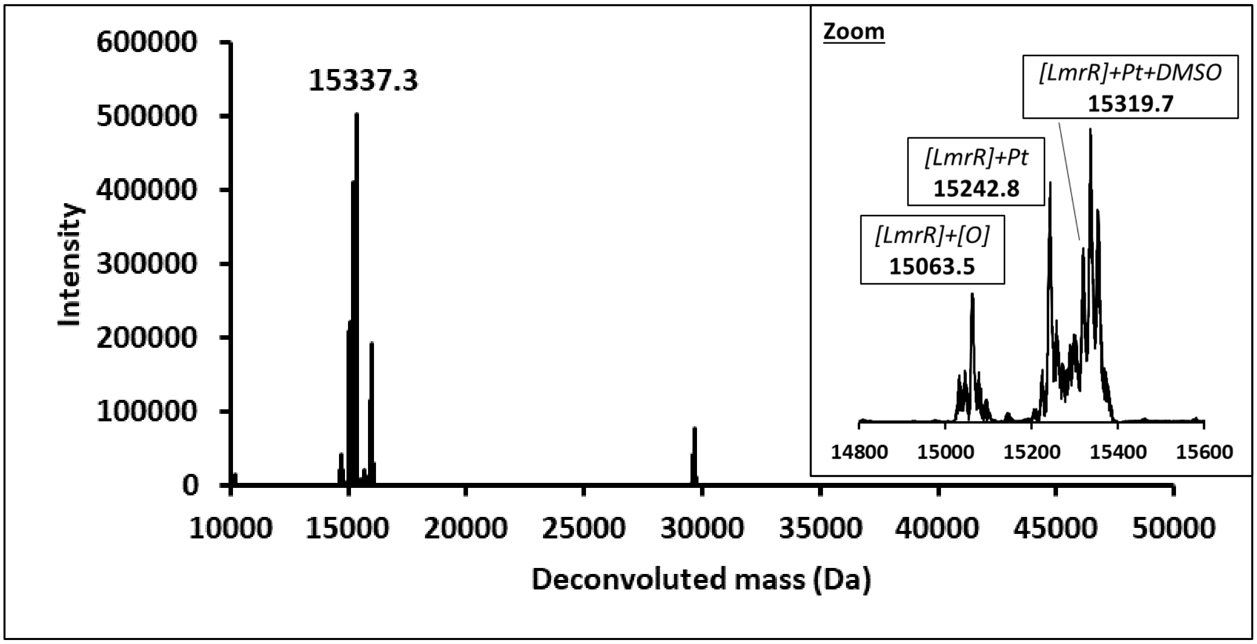

LmrR_V15pSHF + Au(SMe$_2$)Cl – column

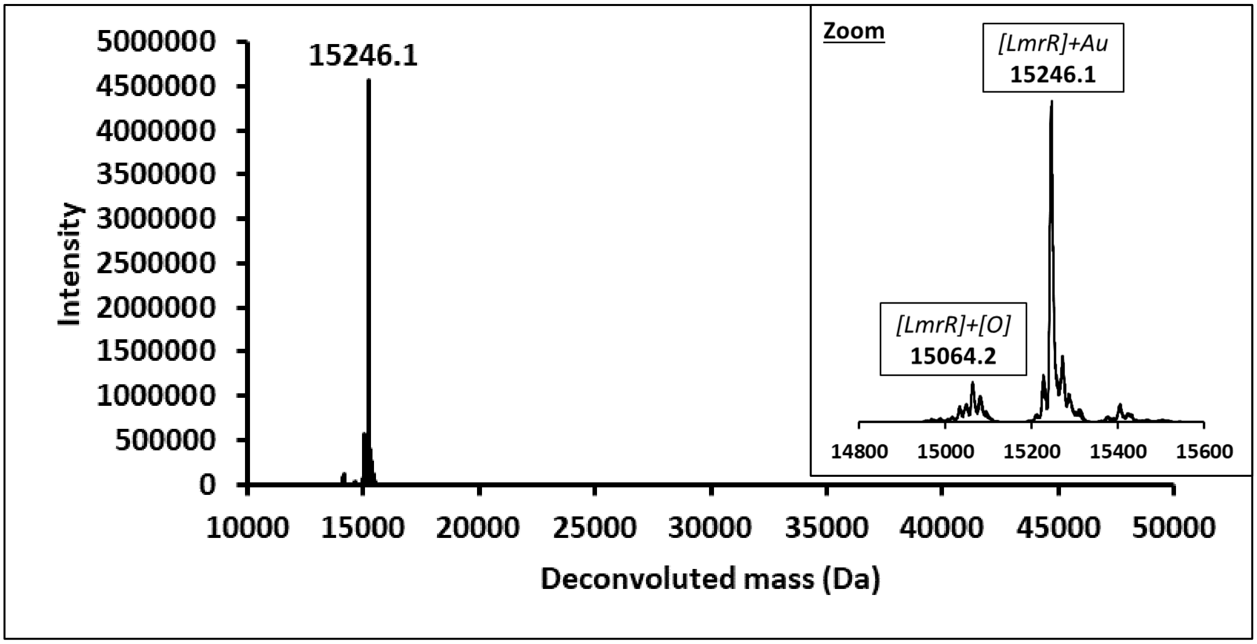

LmrR_V15pSHF + NaAuCl$_4$ – column

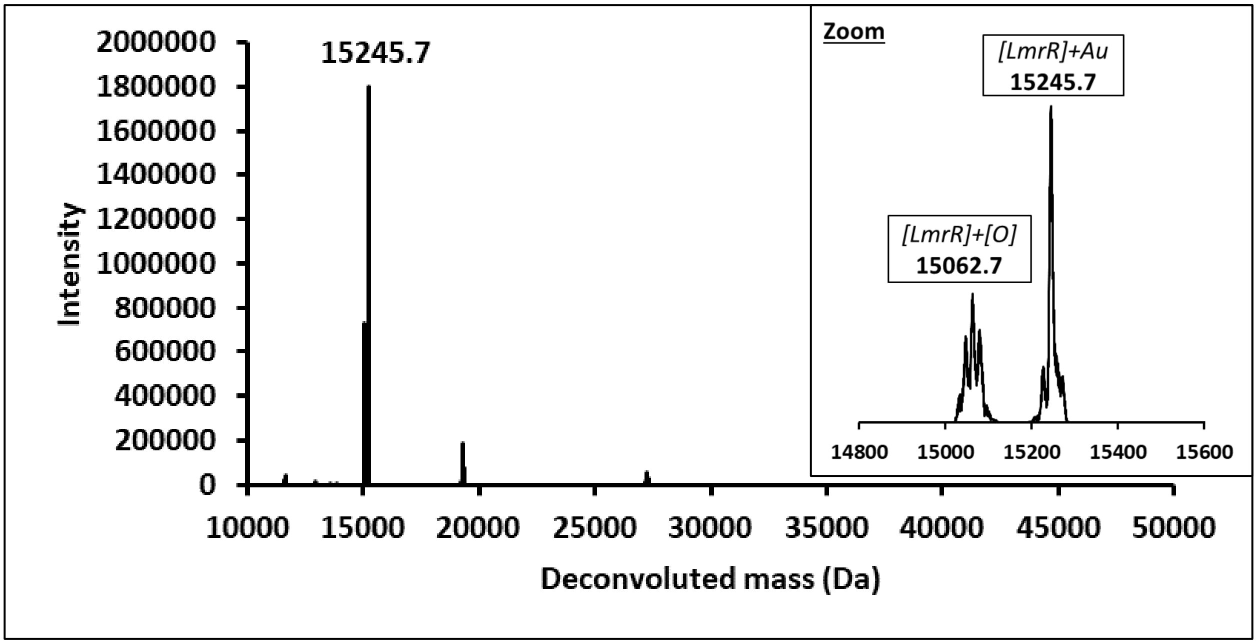

LmrR_V15pSHF + Au(SMe$_2$)Cl – BP1

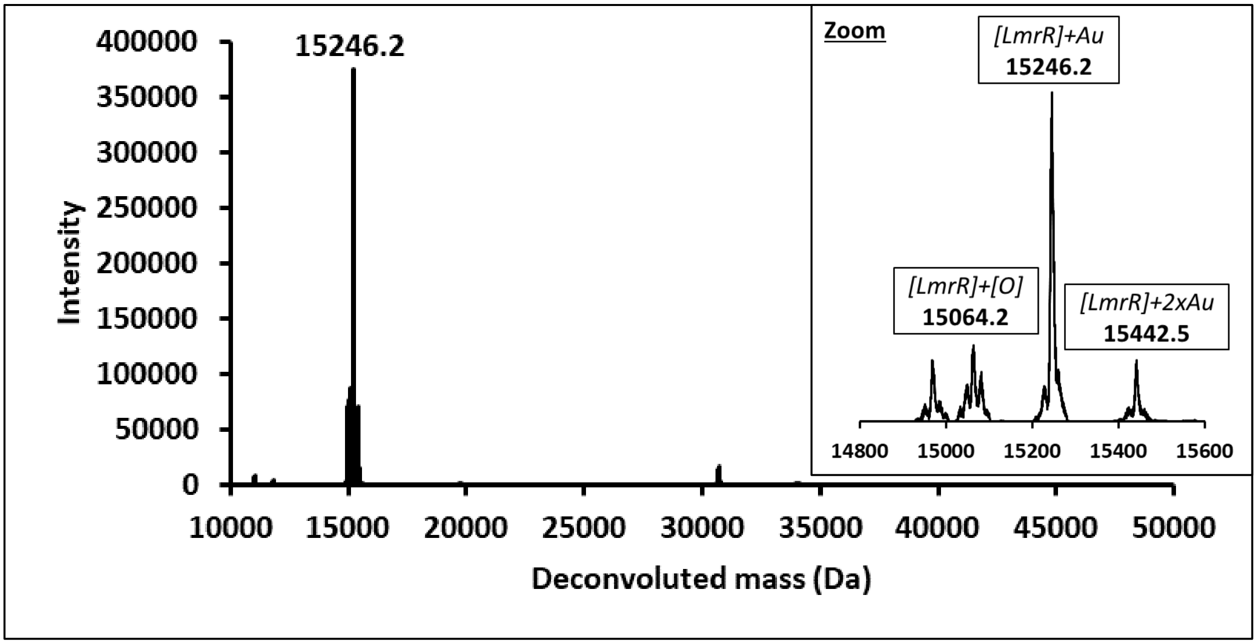

LmrR_V15pSHF + NaAuCl$_4$ – BP1

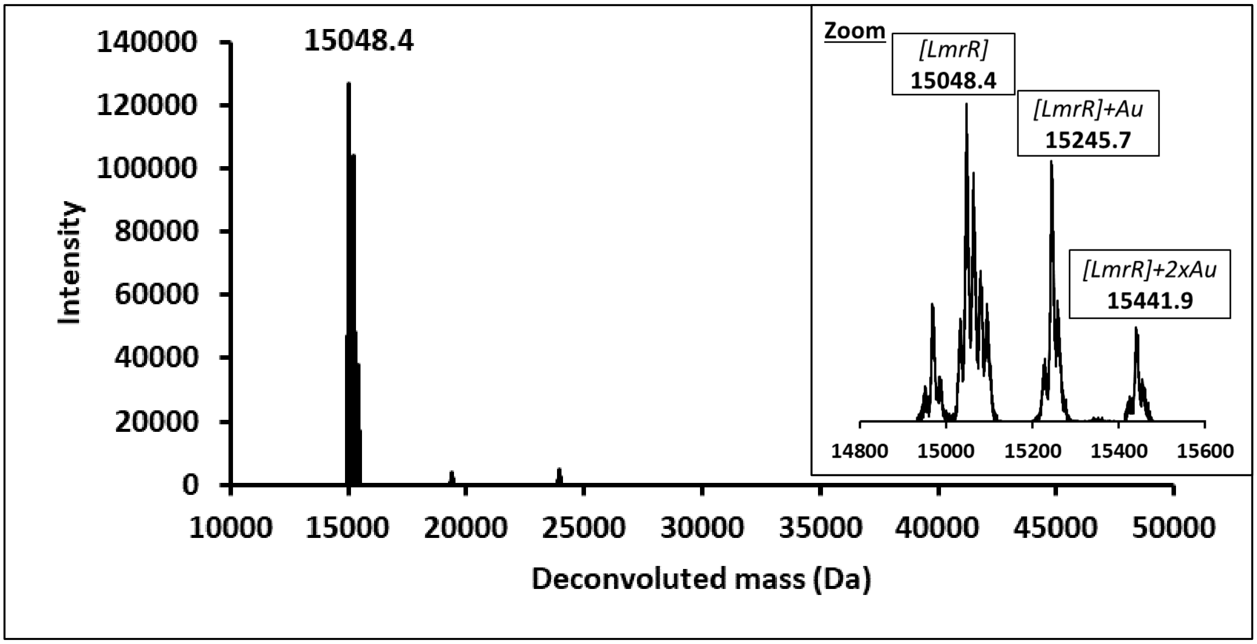

LmrR_V15C + Au(SMe₂)Cl – column

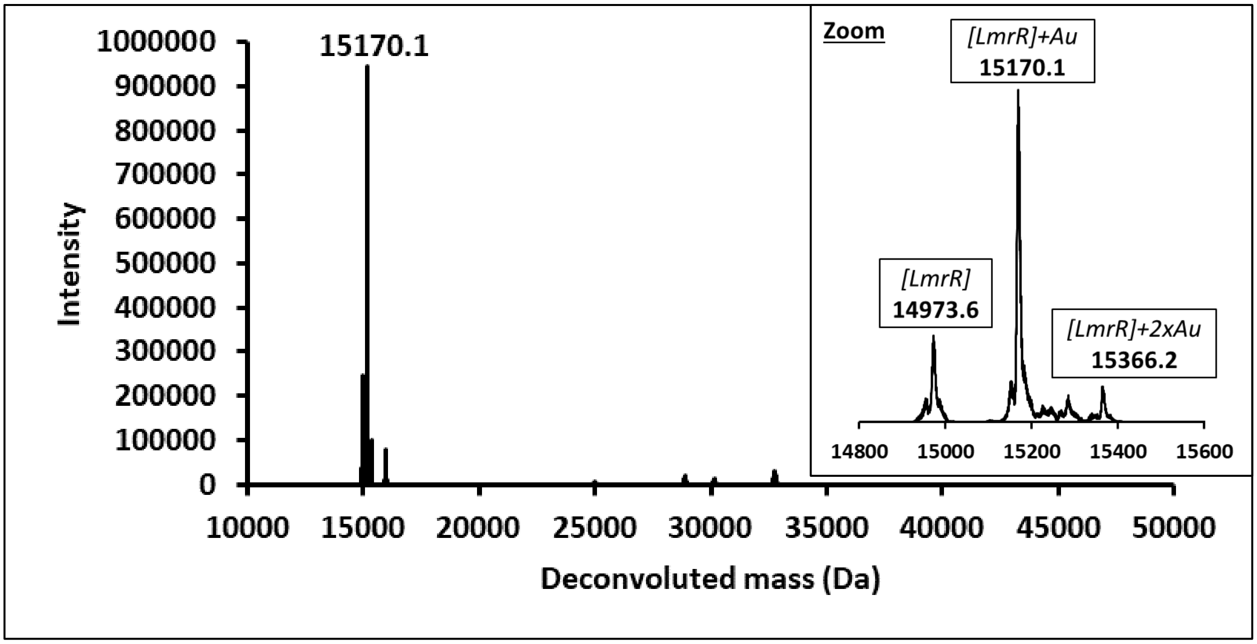

LmrR_WT + Au(SMe₂)Cl – column

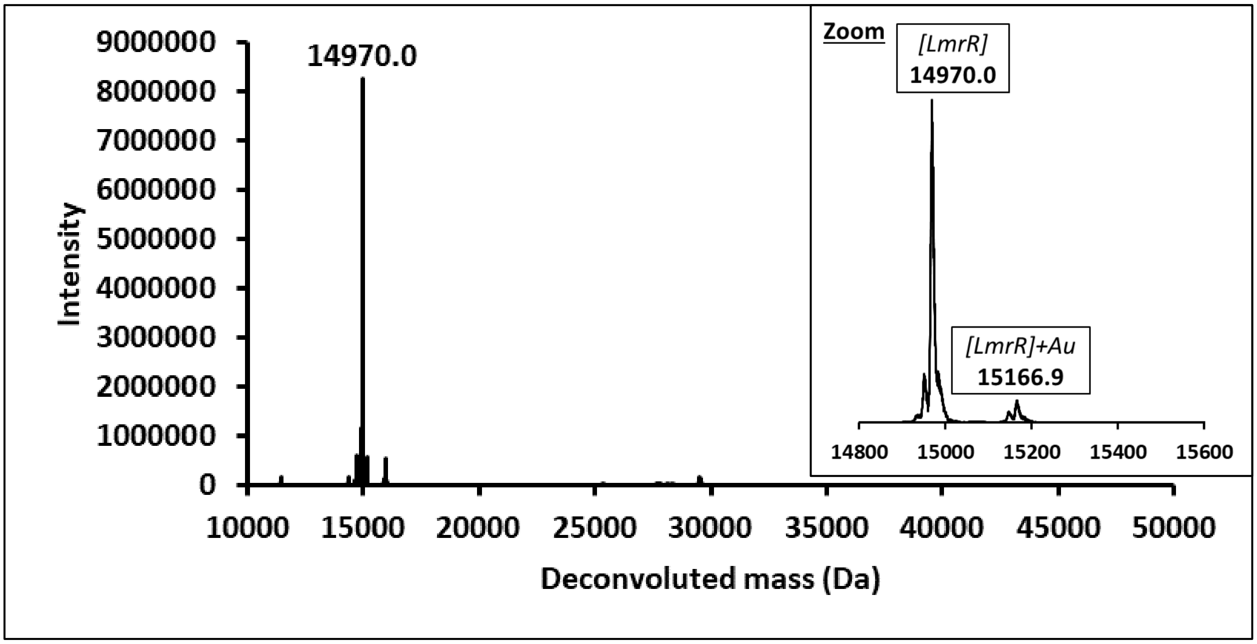

LmrR_V15pSHF + Pd(OAc)$_2$ – BP1

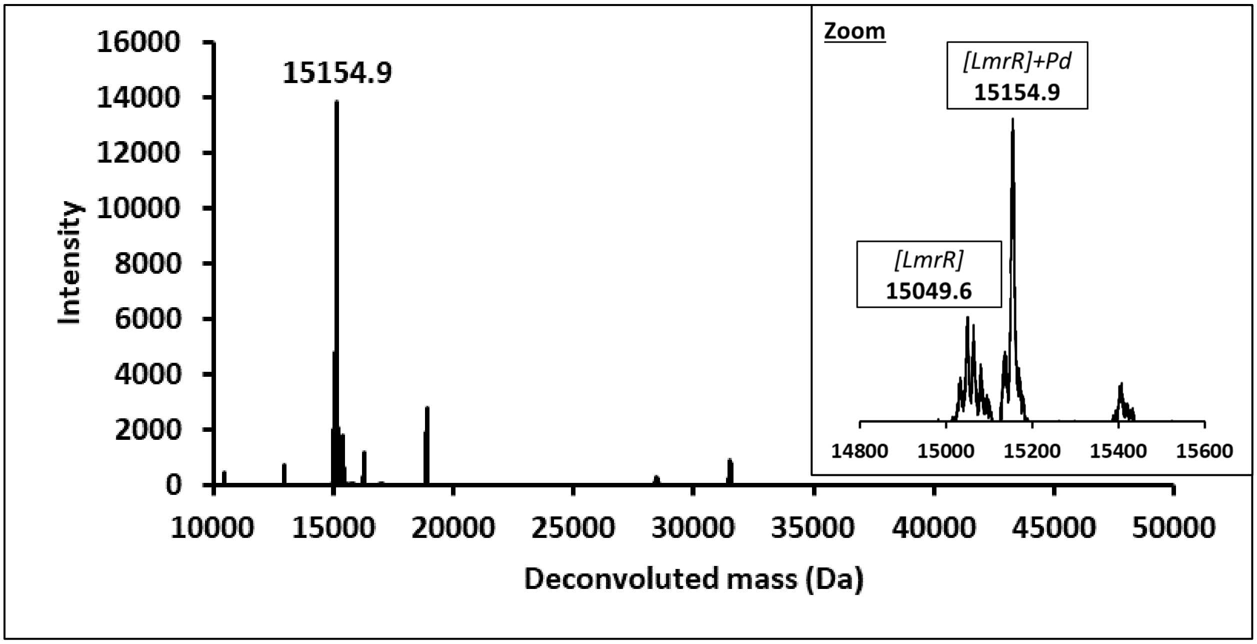

SI.7 [Au]-titration using UV-vis spectroscopy (LmrR_WT and thiophenol)

While [Au]-titration to LmrR_V15*p*SHF gives clear changes in absorption bands upon increasing concentrations of Au(SMe_2)Cl, the absorption spectra of LmrR_WT are unaffected with increasing Au(SMe_2)Cl concentrations (Figure S12). This indicates the difference in binding interactions between LmrR_V15*p*SHF and LmrR_WT. In addition, when thiophenol is subjected to increasing Au(SMe_2)Cl concentrations, the disappearance of absorption bands is observed, which is consistent with the formation of insoluble clusters (Figure S13).

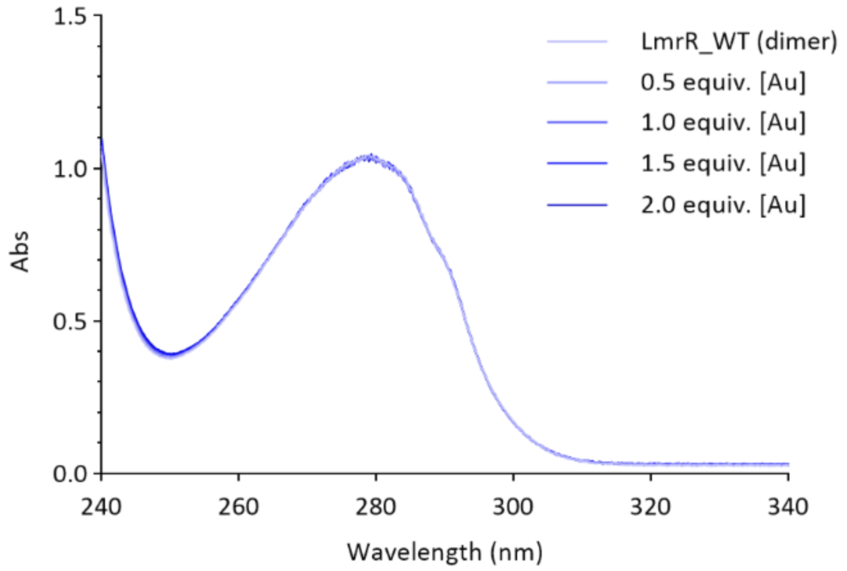

Figure S12. UV-vis spectrum of 0, 0.5, 1.0, 1.5, 2.0 equivalence of Au(SMe_2)Cl titrated to LmrR_WT (dimer)

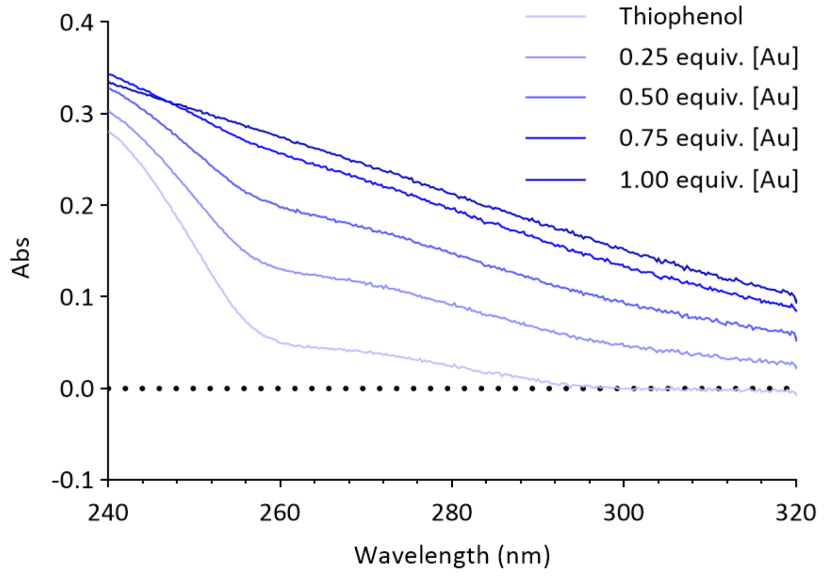

Figure S13. UV-vis spectrum of 0, 0.25, 0.5, 0.75, 1.00 equivalence of Au(SMe_2)Cl titrated to thiophenol

SI.8 CD-spectroscopy of LmrR_V15*p*SHF-Au

The CD-spectra of LmrR_V15*p*SHF-Au and the apo-protein LmrR_V15*p*SHF are identical (Figure S14). Therefore, the secondary structure of the protein scaffold is still intact upon gold-binding.

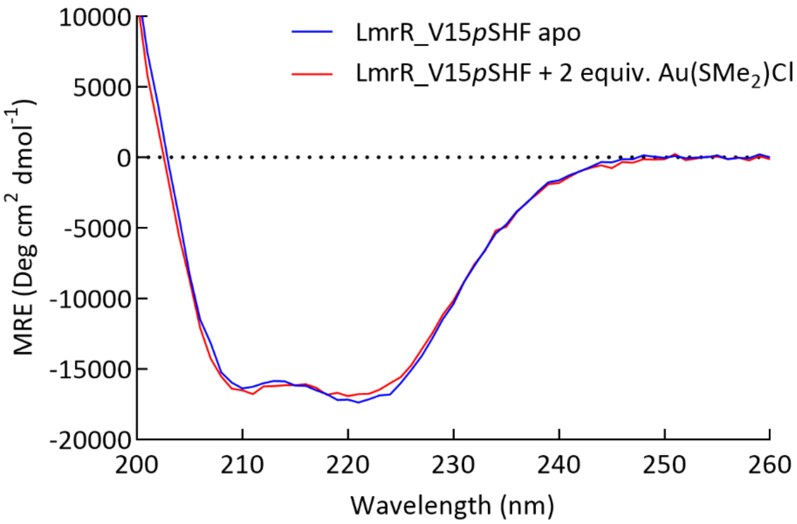

Figure S14. Comparison of CD-spectra of apo LmrR_V15*p*SHF and LmrR_V15*p*SHF after incubation with Au(SMe_2)Cl.

SI.9 Structural analysis of Au-bound LmrR_V15*p*SHF

The crystal structure of Au-bound LmrR_V15*p*SHF, obtained by co-crystallisation with $KAuCN_2$, reveals a gold atom coordinated to one of the two *p*SHF residues (bond distance S-Au = 2.4 Å). The position of the Au atom coincides with the highest peak in the anomalous difference Fourier map (Figure S15). The atom is pointing away from the tryptophan residues in the middle of the hydrophobic pocket. Only one gold atom could be unambiguously positioned into the crystal structure based on the electron density maps. No significant anomalous difference Fourier peak was detected near the other *p*SHF residue in the LmrR dimer. The $2F_o$-F_c electron density map for this second *p*SHF residue reveals significant disordering of its side chain.

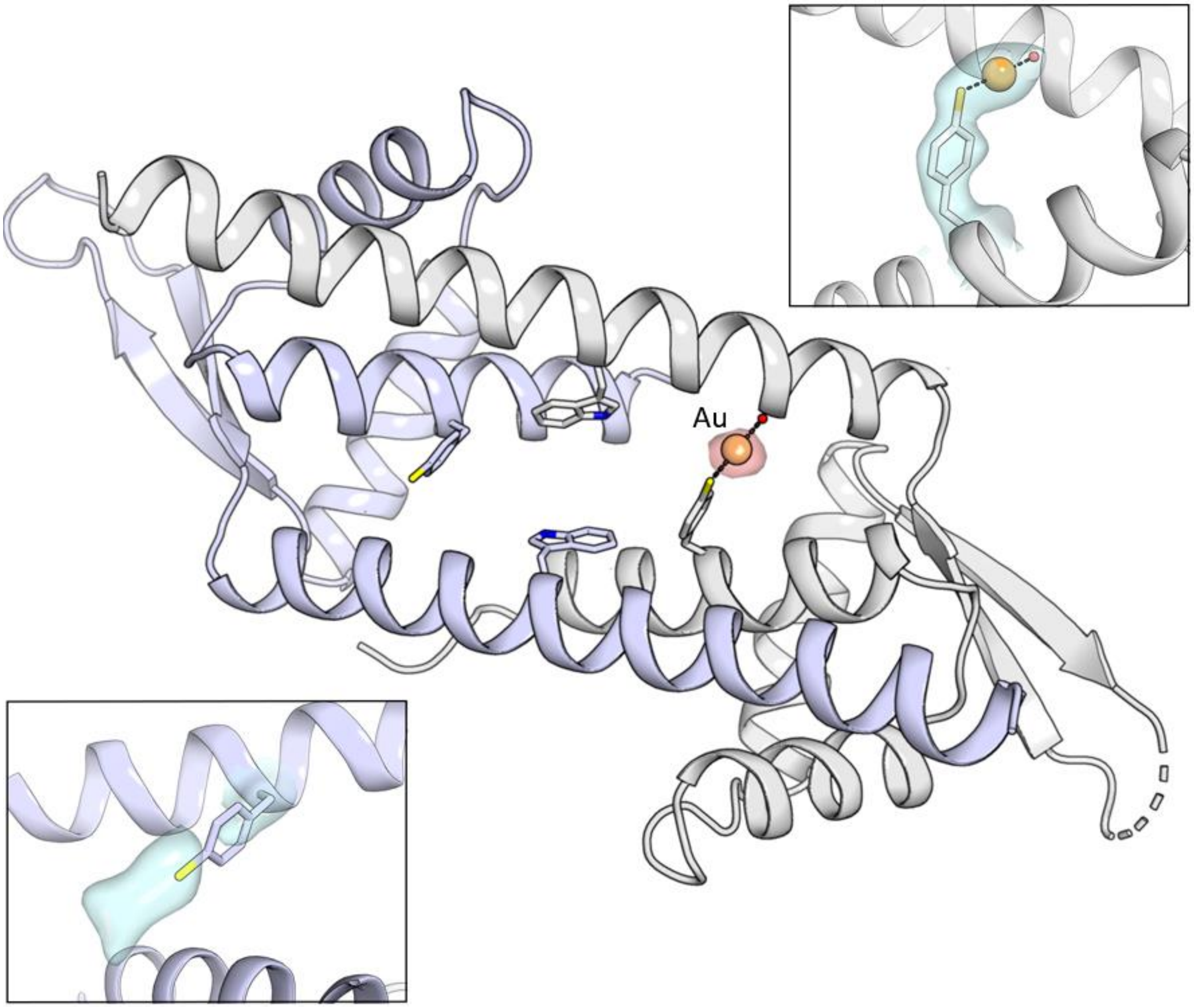

Figure S15. Crystal structure of dimeric Au-bound LmrR_V15*p*SHF (PDB: 9G52), showing the two *p*SHF residues as sticks and the bound gold ion as a sphere. The density around the gold ion is the highest peak in the anomalous difference Fourier map (contoured at 5σ). The insets show zoomed-in views of the *p*SHF residues, together with their associated $2F_o$-F_c electron densities in cyan (contoured at 1σ). The putative water or hydroxide ion coordinating the gold ion is shown as a small red sphere (bond distances Au-S = 2.4 Å, Au-O = 2.3 Å).

SI.10 Metal precursor screening for biocatalytic hydroamination

With a set of noble metals in hand that could bind LmrR_V15*p*SHF, we probed the use of LmrR_V15*p*SHF in transition metal catalysis using the hydroamination of 2-ethynylaniline (**1a**) to indole (**2a**). Under the given conditions, substrate **1a** was fully converted using either an Au(I) or Au(III) precursor (Table S4).

A common side reaction that is observed is the hydration of the alkyne. This is seen in the hydroamination catalysed by Au, but also in the reactions catalysed by other noble metals (SI.30, 2-amino acetophenone). However, this does not account for all of the side reactions. If the substrate is incubated without metal or protein under reaction conditions, also part of the substrate is converted, suggesting uncatalysed side reactions.

Table S4. List of attempted biocatalytic hydroaminations using different transition metal precursors.

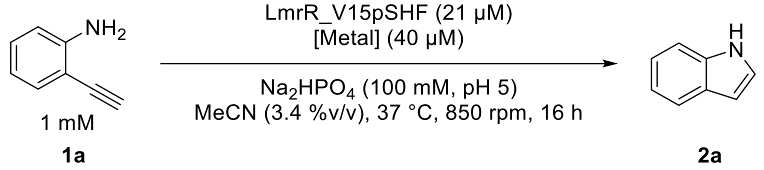

Entry	Metal	Yield (%)[a] (no protein)	Conversion (%)[a] (no protein)	TON
1	$RuCp(MeCN)_3PF_6$	2 (1)	31 (28)	<1
2	$[Rh(COD)Cl]_2$ (20 μM)	1 (1)	34 (32)	<1
3	$Pt(COD)Cl_2$	1 (2)	38 (34)	<1
4	$Pt(DMSO)Cl_2$	1 (1)	30 (38)	<1
5	$Au(SMe_2)Cl$	66 (6)	100 (41)	33
6	$NaAuCl_4$	76 (4)	100 (58)	38

Reaction conditions: 1 mM substrate **1a**, 2 mol% ArM, 100 mM Na_2HPO_4, pH 5, 3.4 %v/v MeCN, 37 °C, 850 rpm, 16 h
[a] Yields and conversions are obtained by GC-FID using mesitylene as internal standard.
Results are obtained as single measurement.

SI.11 Reaction optimisation

Under the standard conditions, substrate **1a** is converted to indole (**2a**) using Au(SMe_2)Cl as precursor. A pH screening showed that pH 5 was the optimum pH, giving the highest yield. Changing the reaction medium to Na_2HPO_4 had no significant effect on the yield. While pH 5 is outside of the buffering range of MOPS, the pH remains constant throughout the reaction, which we confirmed by measuring the pH before and after the reaction. Lowering the reaction temperature to 25 °C gave a small reduction in yield, however the mass balance of the reaction improved. Prolonging the reaction time increases the conversion, but the yield does not change significantly. Using $NaAuCl_4$ as precursor gave comparable yields as Au(SMe_2)Cl. However, oxidative damage on the protein was observed upon binding $NaAuCl_4$. Additionally, we performed the reaction with **1a** and **6a** using $KAuCN_2$ as precursor, which was used to obtain the Au-bound LmrR_V15pSHF crystal structure. However, in both cases the gold enzyme precipitated during the reaction and no product formation was observed. Therefore, we continued with Au(SMe_2)Cl as precursor (Table S5).

Table S5. List of optimisation experiments.

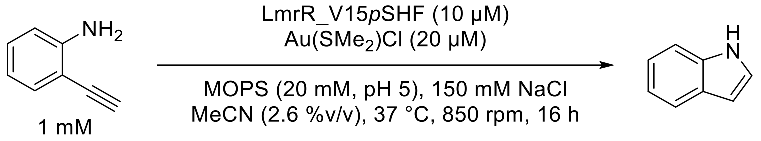

1a → **2a**

Entry	Deviation from standard conditions	Yield (%)[a]	Conversion (%)[a]	TON
1	-	56±12	87±7	56±12
2	pH 4	11±0	27±4	11±0
3	pH 6	45±4	67±2	45±4
4	pH 7	29±11	52±12	29±11
5	pH 8	17±4	41±2	17±4
6	$NaAuCl_4$	60±9	87±7	60±9
7	100 mM Na_2HPO_4 (no NaCl)	55±3	83±4	55±3
8	RT	43±5	51±6	43±5
9	25 h	52±3	93±4	52±3

Reaction conditions: 1 mM substrate **1a**, 1 mol% ArM, 20 mM MOPS + 150 mM NaCl, pH 5, 2.6 %v/v MeCN, 37 °C, 850 rpm, 16 h
[a] Yields and conversions are obtained by GC-FID using mesitylene as internal standard.
Results are obtained as an average of two experiments, errors are given as ±(standard deviation).

SI.12 Control experiments relevance of *p*SHF residue

The *p*SHF residue is important for achieving the activity of the biocatalytic hydroamination. We compared various LmrR variants with Au(SMe_2)Cl in the hydroamination of substrate **1a**. These include LmrR wild-type (LmrR_WT) and various amino acids incorporated at position 15, including cysteine (LmrR_V15C), tyrosine (LmrR_V15Y), *para*-aminophenylalanine (*p*AF), and 2,2-bipyridinealanine (LmrR_V15BpyAla). Other LmrR variants can still promote basal catalysis compared to free gold in solution. Therefore, the LmrR scaffold likely harbours aspecific binding sites for gold. However, LmrR_V15*p*SHF-Au gives at least a 2-fold increase in TON compared to the other LmrR variants (Table S6), which indicates a unique environment for the catalytic gold ion.

Table S6. List of control experiments by using different LmrR-variants.

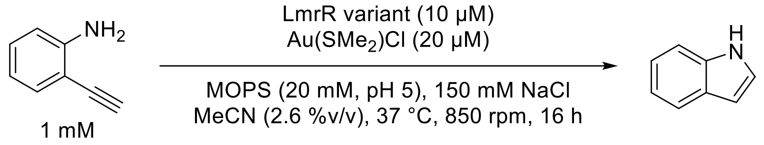

1a **2a**

Entry	LmrR-variant	Yield (%)[a]	Conversion (%)[a]	TON
1	LmrR_V15*p*SHF	56±12	87±7	56±12
2	LmrR_WT	21±4	64±2	21±4
3	LmrR_V15C	9±1	54±1	9±1
4	LmrR_V15Y	17±3	56±2	17±3
5	LmrR_V15*p*AF	17±0	64±1	17±0
6	LmrR_V15BpyAla	19±0	60±2	19±0

Reaction conditions: 1 mM substrate **1a**, 1 mol% ArM, 20 mM MOPS + 150 mM NaCl, pH 5, 2.6 %v/v MeCN, 37 °C, 850 rpm, 16 h
[a] Yields and conversions are obtained by GC-FID using mesitylene as internal standard.
Results are obtained as an average of two experiments, errors are given as ±(standard deviation).

SI.13 Control experiments catalyst components

Both the Au(SMe$_2$)Cl and LmrR_V15*p*SHF are pivotal components in the catalytic hydroamination. When either of the components is removed, only product traces are observed. If only 1 equiv. of Au(SMe$_2$)Cl relative to LmrR_V15*p*SHF dimer is used, no product is observed. If after the assembly of LmrR_V15*p*SHF-Au an excess of TCEP is added, the enzyme is inactivated (Table S7).

Table S7. List of control experiments by omission of reaction components or addition of a metal scavenger (TCEP).

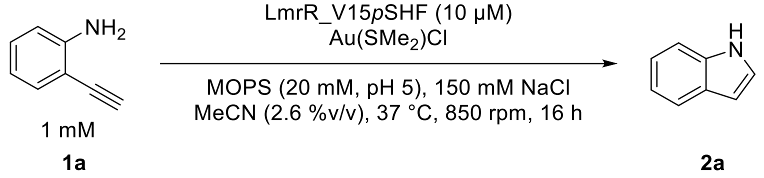

Entry	Au(SMe$_2$)Cl	Deviation from standard conditions	Yield (%)[a]	Conversion (%)[a]	TON
1	20 µM (2 equiv.)	-	56±12	87±7	56±12
2	10 µM (1 equiv.)	-	0±0	27±0	0±0
3	0 µM (0 equiv.)	-	0±0	26±1	0±0
4	20 µM (2 equiv.)	No protein	4±0	33±2	4±0
5	20 µM (2 equiv.)	100 µM TCEP added	4±0	43±0	4±0

Reaction conditions: 1 mM substrate **1a**, 1 mol% ArM, 20 mM MOPS + 150 mM NaCl, pH 5, 2.6 %v/v MeCN, 37 °C, 850 rpm, 16 h
[a] Yields and conversions are obtained by GC-FID using mesitylene as internal standard.
Results are obtained as an average of two experiments, errors are given as ±(standard deviation).

SI.14 Regioselectivity of the hydroaminase

Hydroamination of substrate **3** can form two different cyclisation products, either via 6-*exo-dig* cyclisation (**4**), or via 5-*endo-dig* cyclisation (**5**). Even though LmrR_WT can promote the hydroamination reaction, it shows the same regioselectivity as free gold in solution. Even though the yield increases upon higher Au-equiv. compared to LmrR_WT, the selectivity is not affected (Table S8). On the contrary, the regioselectivity that is obtained with LmrR_V15*p*SHF-Au largely depends on the stoichiometry of $Au(SMe_2)Cl$. Similar to the hydroamination of substrate **1a**, <1 equiv. [Au] results in no product formation. At 1.5-2 equiv. [Au] near complete selectivity towards product **4** is observed. Upon further increasing the [Au] equiv., the same selectivity is approached as free gold in solution (Table S9).

Table S8. List of control experiments with different [Au]-stoichiometries in the hydroamination of substrate **3**.

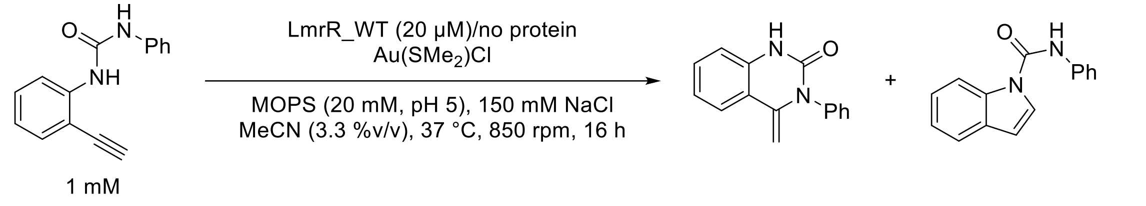

Entry	Control	[Au]-equiv.	Total yield[a]	Selectivity (**4**:**5**)[a]
1	LmrR_WT	1 (20 μM)	17±2	(67:33)
2	LmrR_WT	2 (40 μM)	58±2	(61:39)
3	LmrR_WT	3 (60 μM)	79±2	(63:37)
4	LmrR_WT	4 (80 μM)	84±3	(63:37)
5	No protein	20 μM	5±1	(65:35)
6	No protein	40 μM	8±0	(66:34)
7	No protein	60 μM	11±1	(69:31)
8	No protein	80 μM	12±0	(67:33)

Reaction conditions: 1 mM substrate **3**, 2 mol% ArM, 20 mM MOPS + 150 mM NaCl, pH 5, 3.3 %v/v MeCN, 37 °C, 850 rpm, 16 h
[a] Yields and selectivities are obtained by SFC using 2-phenylquinoline as internal standard.
Results are obtained as an average of two experiments, errors are given as ±(standard deviation).

Table S9. List of different [Au]-stoichiometries compared to LmrR_V15*p*SHF affecting the hydroamination of substrate **3**.

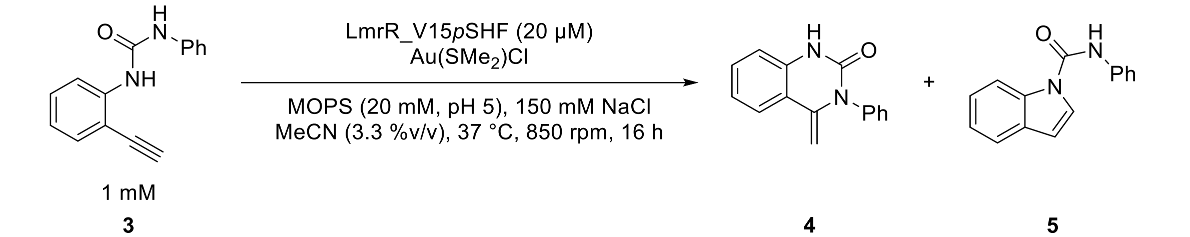

Entry	Au-equiv.	Total yield[a]	Selectivity (**4**:**5**)[a]
1	0, 0.5, 1 (0-20 μM)	0	N/A
2	1.5 (30 μM)	18±4	(100:0)
3	2 (40 μM)	66±9	(98:2)
4	2.5 (50 μM)	82±2	(91:9)
5	3 (60 μM)	82±5	(74:26)
6	3.5 (70 μM)	95±2	(68:32)
7	4 (80 μM)	90±15	(69:31)
8	4.5 (90 μM)	89±17	(66:34)
9	5 (100 μM)	89±6	(69:31)

Reaction conditions: 1 mM substrate **3**, 2 mol% ArM, 20 mM MOPS + 150 mM NaCl, pH 5, 3.3 %v/v MeCN, 37 °C, 850 rpm, 16 h
[a] Yields and selectivities are obtained by SFC using 2-phenylquinoline as internal standard.
Results are obtained as an average of two experiments, errors are given as ±(standard deviation).

SI.15 Validation of screening protocol LmrR_*p*SHF variants

The initial rate of product formation using substrate **6a** was determined using a fluorescence plate reader assay. Product formation can be detected at $\lambda_{excitation}$= 320 nm and $\lambda_{emission}$ = 365 nm. We tested the reaction in the plate reader by varying the LmrR_V15*p*SHF-Au concentration and measuring the fluorescence intensity over time. The fluorescence output shows a linear dependency with enzyme concentration (Scheme S2). Therefore, it can be a suitable screening method to distinguish reactivity differences.

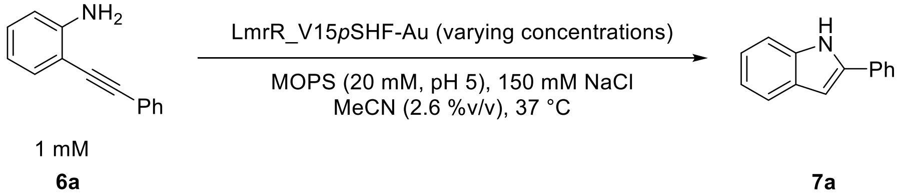

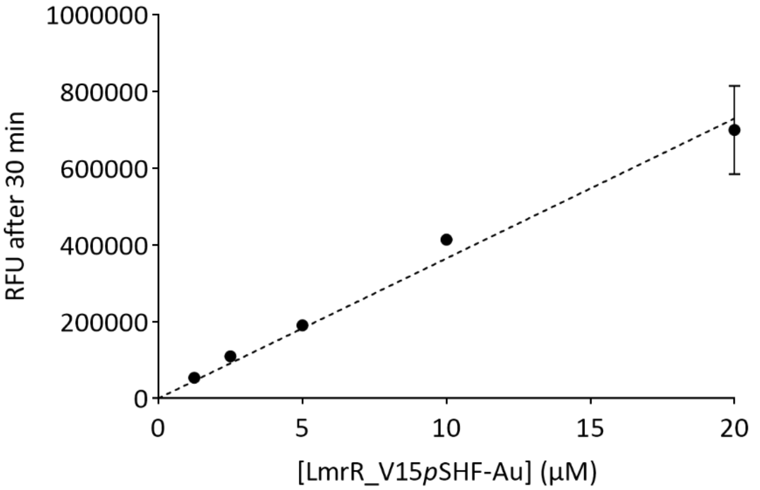

Scheme S2. Dependency of LmrR_V15*p*SHF-Au concentration on product **7a** formation over time in 96-well fluorescence plate screen. Results are obtained as an average of two experiments, errors are given as ±(standard deviation).

SI.16 Positional screening of the *p*SHF residue

Screening for LmrR variants in lysates resulted in inconsistent results. Therefore, after enzymatic lysis of the cells by lysozyme, the LmrR variants were immobilised on streptavidin beads and washed before addition of Au(SMe_2)Cl. Using this screening we incorporated the *p*SHF residue at various positions in LmrR. The results of this screening shows that ArMs with the pSHF at positions 8, 11, 15 and 19 have increased activity compared to LmrR_WT. The screening showed that position 15 accommodates the best initial rate of the reaction (Figure S16).

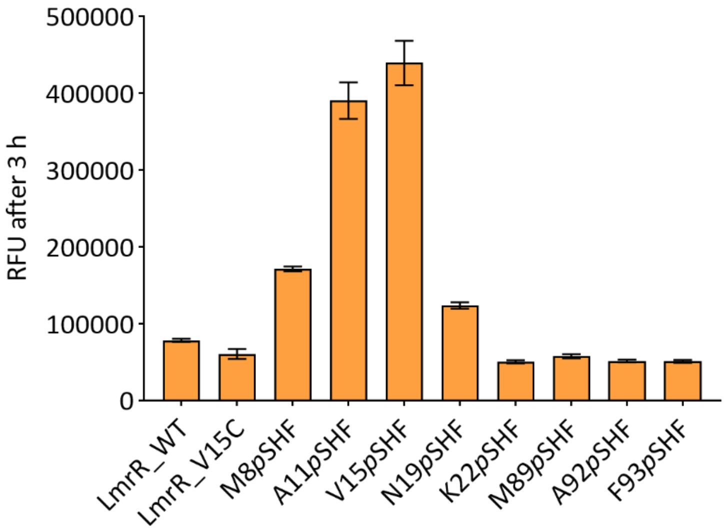

Figure S16. Fluorescence plate screening results from positional screening of *p*SHF residue. Results are obtained as an average of two experiments, errors are given as ±(standard deviation).

SI.17 Alanine scanning

We performed an alanine scanning to identify residues that have a significant contribution to the activity of the biocatalytic hydroamination reaction. While most alanine substitutions retained similar initial rates as the parent, four substitutions were identified to have a significant effect on the initial reaction rate. The A11L variant showed an increase in rate and the M8A, L18A and M89A variants showed a significant decrease in rate (Figure S17). From the crystal structure it can be seen that these four residues are in close proximity of the *p*SHF residue. We confirmed that the effect of the A11L and M89A substitution on the hydroamination yield were consistent with the observed activity from the plate screen (Table S10).

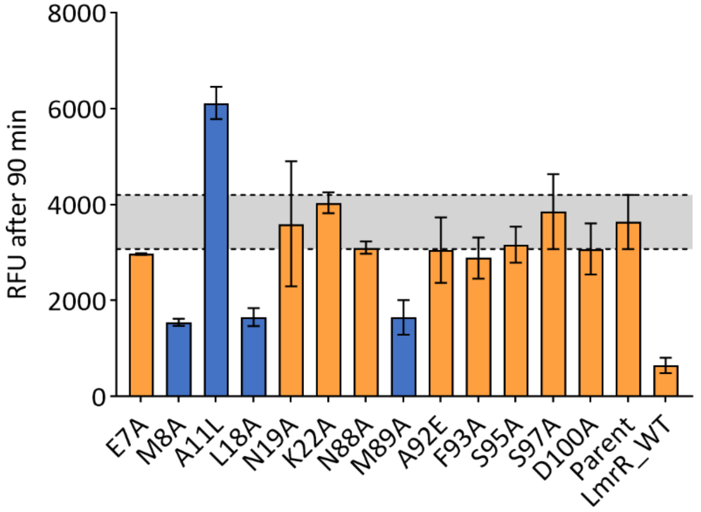

Figure S17. Fluorescence plate screening results from alanine scanning. Results are obtained as an average of two experiments, errors are given as ±(standard deviation).

Table S10. Comparison of alanine scanning hits and controls with purified proteins.

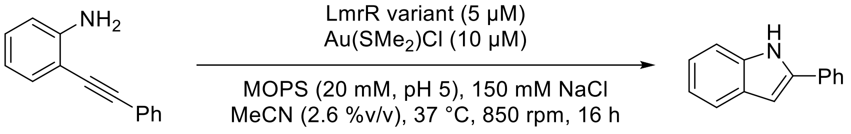

1 mM
6a

7a

Entry	LmrR variant	Yield[a]	TON
1	LmrR_V15*p*SHF	39±6	79±11
2	LmrR_V15*p*SHF_A11L	46±2	92±4
3	LmrR_V15*p*SHF_M89A	17±2	34±4
4	LmrR_WT	8±1	16±2
5	No protein	4±1	8±1

Reaction conditions: 1 mM substrate **6a**, 0.5 mol% ArM, 20 mM MOPS + 150 mM NaCl, pH 5, 2.6 %v/v MeCN, 37 °C, 850 rpm, 16 h
[a] Yields and selectivities are obtained by GC-FID using mesitylene as internal standard.
Results are obtained as an average of two experiments, errors are given as ±(standard deviation).

SI.18 Site-saturation mutagenesis

First, we randomised position A11, which is adjacent to the *p*SHF residue. In the screening various substitutions showed an improvement over the parent (Figure S18). We purified six variants and confirmed that A11L was the best substitution (Figure S19). Since the crystal structure indicated that methionine 89 is in close proximity of the gold, we randomised position 89 as well. We included the A11L substitution in the template and found that any mutation of M89 is deleterious (Figure S20).

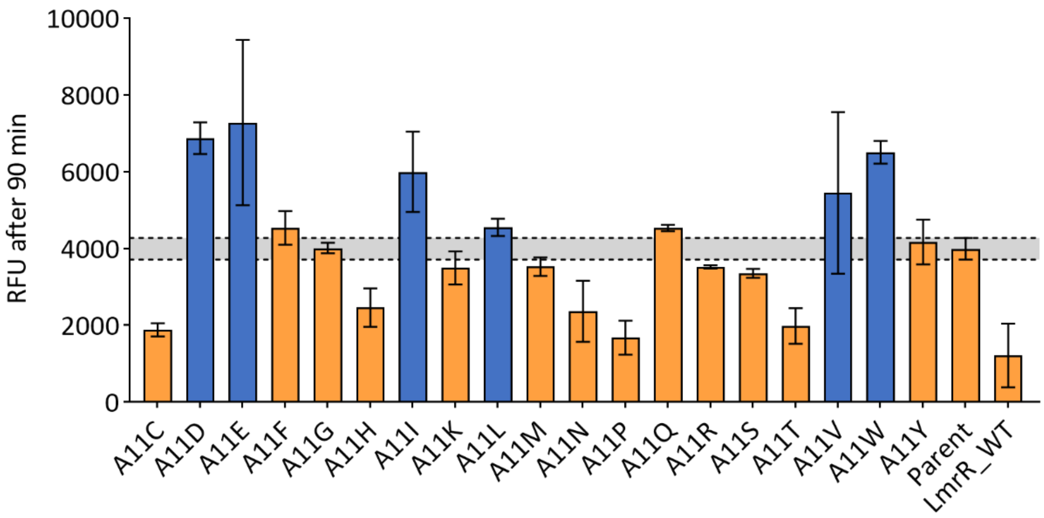

Figure S18. Fluorescence plate screening results from A11 site-saturation mutagenesis. Results are obtained as an average of two experiments, errors are given as ±(standard deviation).

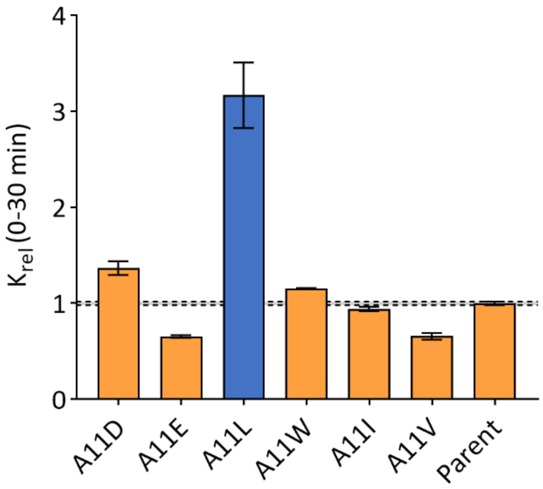

Figure S19. Fluorescence plate screening results from A11-hits with purified proteins. Results are obtained as an average of two experiments, errors are given as ±(standard deviation).

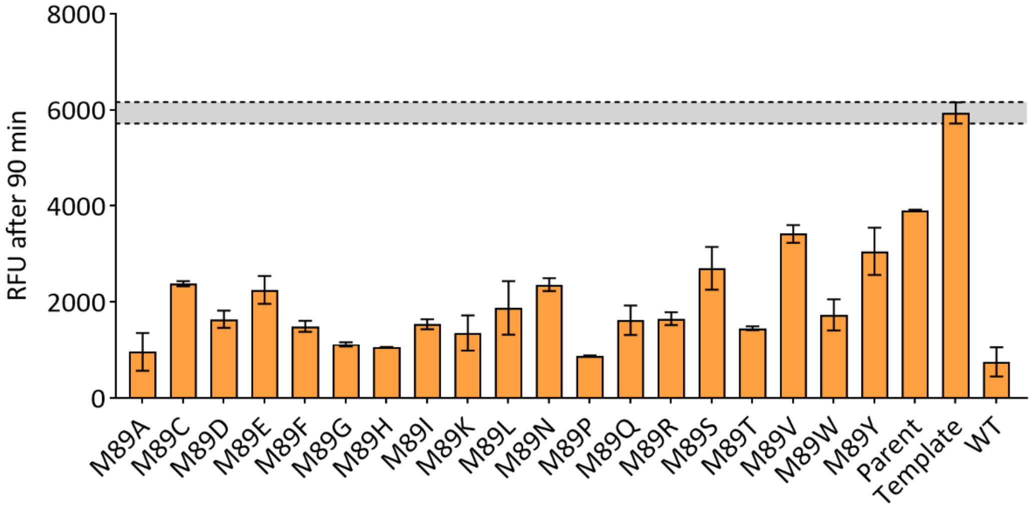

Figure S20. Fluorescence plate screening results from M89 site-saturation mutagenesis. Results are obtained as an average of two experiments, errors are given as ±(standard deviation).

SI.19 Comparison kinetic properties of improved variant and parent

To characterise the improvement of the A11L substitution we attempted Michaelis-Menten kinetics. We were not able to reach saturation, due to the poor solubility of substrate **6a** (Figure S21). In addition, while we qualitatively screened for better variants via fluorescence, quantification was limited due to the inner-filter effect. Therefore, the reaction was quenched at various time points and the formation of product **7a** was measured using UV absorption via SFC (Figure S22). We found a ~2.3-fold increase in catalytic efficiency for LmrR_V15*p*SHF_A11L-Au compared to the parent (Table S11).

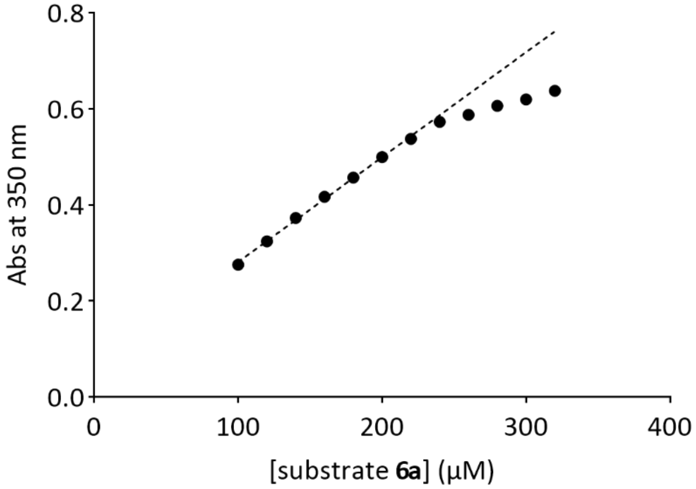

Figure S21. The 350 nm absorbance of substrate **6a** as a function of substrate concentration indicating poor substrate solubility

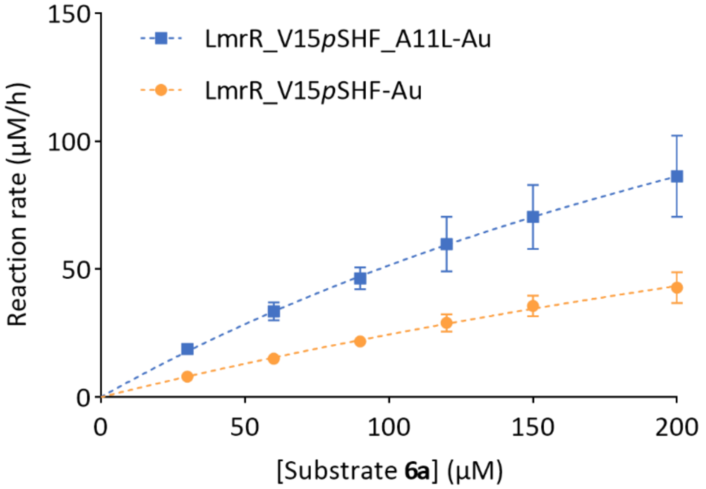

Figure S22. Measured reaction rates with respect to substrate concentration of LmrR_V15*p*SHF-Au and LmrR_V15*p*SHF_A11L-Au. Results are obtained as an average of four experiments (two duplicates of two different batches of enzyme), errors are given as ±(standard deviation).

Table S11. Comparison of kinetic parameters of LmrR_V15*p*SHF-Au and LmrR_V15*p*SHF_A11L-Au

	$(K_{cat}/K_M)\ \mu M^{-1}h^{-1}$ *Best fit*	$(K_{cat}/K_M)\ \mu M^{-1}h^{-1}$ 95% CI	Fold increase
LmrR_V15*p*SHF-Au	0.28	0.024-3.31	-
LmrR_V15*p*SHF_A11L-Au	0.64	0.066-6.32	2.3

SI.20 General considerations

Chemicals were purchased from Sigma-Aldrich, TCI, BLD-Pharm, Fisher scientific and Fluorochem/Doug Discovery and used without further purification. Flash column chromatography was performed using SiliaFlash P60 (40-63 µm, silicycle). ^{1}H-NMR (400 MHz) and ^{13}C-NMR (101 MHz) were recorded using a Agilent Technologies 400/54 premium shielded using DMSO-d_6 or $CDCl_3$ as solvent. The NMR data is reported as: chemical shift using residual solvent peaks as reference, multiplicity, coupling (Hz) and integration. *E. coli* strains XL1-blue, NEB10-β, BL21(DE3) and BL21(DE3) C43 were used for cloning and expression. *V. natriegens* (Vmax™ X2) competent cells were purchased from BioCat GmbH (Heidelberg, Germany). DENERASE® was purchased from c-LEcta GmbH (Leipzig, Germany). Primers were synthesised by Sigma-Aldrich, plasmid purification was performed using kits from QIAGEN and Phusion polymerase and Dpn1 were purchased from New England Biolabs. DNA sequencing was carried out by Eurofins Genomics. Strep-Tactin® Superflow® high capacity beads from IBA-Lifesciences were used to immobilise LmrR variants during protein purification and library screening. Desthiobiotin from IBA-Lifesciences was used for protein purification. A Thermo Scientific Nanodrop 2000 UV-vis spectrophotometer was used to measure absorption to determine concentrations of protein (280 nm) and DNA (260 nm or 280 nm). Molar extinction coefficients at 280 nm for proteins were calculated using the ProtParam Expasy web server https://web.expasy.org/protparam/ and corrected for the presence of unnatural amino acids. Proteins were characterised via LRMS using a Waters Acquity H-class UPLC with Waters Xevo G2 QTOF or HRMS using a Thermo LTQ Orbitrap XL. Biocatalytic reactions were analysed using GC-FID (Shimadzu GC-2014) or SFC (Waters Acquity UPC2).

SI.21 sfGFP assay procedures

OTS screening using sfGFP

pBAD_sfGFP_Y151TAG was co-transformed into *E. coli* BL21(DE3) or *E. coli* BL21(DE3) C43 with various OTS plasmids and a single colony was used to inoculate an overnight culture with 100 µg/mL ampicillin and the respective OTS antibiotic (Table S13). The cultures were stored at -70 °C with 25% glycerol for future use.

Table S13. List of OTSs screened for genetic *p*SHF incorporation with their respective antibiotics.

Entry	OTS	Antibiotic
1	pDULE2_pCNF	Spectinomycin (50 µg/mL)
2	pULTRA_pCNF	Spectinomycin (50 µg/mL)
3	pDULE2_pAF	Spectinomycin (50 µg/mL)
4	pEVOL_ONBY	Chloramphenicol (34 µg/mL)
5	pEVOL_pBpF	Chloramphenicol (34 µg/mL)
6	pEVOL_pAzF	Chloramphenicol (34 µg/mL)
7	pEVOL_NH2Y	Chloramphenicol (34 µg/mL)
8	pEVOL_pBpyAla	Chloramphenicol (34 µg/mL)
9	pEVOL_Pyl	Chloramphenicol (34 µg/mL)
10	pEVOL_PylRS(Mm)	Chloramphenicol (34 µg/mL)
11	pEVOL_PylRS_AF(Mm)	Chloramphenicol (34 µg/mL)

Tubes containing 5 mL LB medium with 100 µg/mL ampicillin and the respective OTS antibiotic were inoculated with *E. coli* BL21(DE3) strains containing the respective OTS plasmids and pBAD_sfGFP_Y151TAG from previously prepared glycerol stocks. The cells were incubated overnight (135 rpm, 37 °C). Samples of the densely grown cultures (50 µL) were transferred to fresh LB medium containing 100 µg/mL ampicillin and the respective OTS antibiotic. The cells were incubated (135 rpm, 37 °C) until an optical density (OD) at 600 nm of 0.3-0.6 was reached. Then, expression was induced with 1M IPTG (5 µL) and 20% L-arabinose (50 µL). Meanwhile, 200 mM stock solutions of ncAAs *p*SHF and *p*AzF were prepared in DMSO. The induced cultures and the ncAAs were transferred to a 96-well plate. Each well contained 198 µL of induced *E. coli* culture expressing sfGFP_Y151TAG and 2 µL of 200 mM ncAA stock. The ncAA *p*AzF in combination with pEVOL_pAzF served as positive control. For the negative controls no ncAA was added to the induced *E. coli* culture. The 96-well plate was placed in a fluorescence plate reader, which was preheated to 30 °C. The plate was shaken continuously, while fluorescence intensity ($\lambda_{excitation}$ = 485 nm and $\lambda_{emission}$ = 528 nm) was measured for 16 h.

Protocol optimisation sfGFP – reduction of pSHF

*p*SHF was isolated as a mixture of monomer and dimer. Therefore we probed the effect of DTT on the expression levels of sfGFP_Y151*p*SHF. Reduction of *p*SHF prior to addition to the expression culture was performed by shaking 200 mM *p*SHF with 400 mM DTT in DMSO at 37 °C for 90 min. This was compared with various concentrations of fresh DTT added to the expression culture. The analogue sfGFP expression protocol of the OTS screening was applied here.

SI.22 Molecular biology procedures (protein expression and mutagenesis)

List of previously prepared plasmids

Plasmid	Ref.	Note
pEVOL_pAzFRS.2.t1	[7]	pEVOL-pAzFRS.2.t1 was a gift from Farren Isaacs (Addgene plasmid # 73546; http://n2t.net/addgene:73546 ; RRID:Addgene_73546).
pDULE2_pCNF	[8]	pDule2-pCNF was a gift from Ryan Mehl (Addgene plasmid # 85495 ; http://n2t.net/addgene:85495 ; RRID:Addgene_85495).
pULTRA_pCNF	[9]	pULTRA-CNF was a gift from Peter Schultz (Addgene plasmid # 48215 ; http://n2t.net/addgene:48215 ; RRID:Addgene_48215).
pDULE2_pAF	[10]	pDule2-para-aminoPhe was a gift from Ryan Mehl (Addgene plasmid # 85503 ; http://n2t.net/addgene:85503 ; RRID:Addgene_85503).
pEVOL_ONBY	[11]	The plasmid pEVOL_NH2Y was a gift from Prof. A. Deiters (University of Pittsburgh).
pEVOL_pBpF	[12]	The plasmid pEVOL_pBpF was a gift from Prof. P. Schultz (The Scripps Research Institute).
pEVOL_pAzF	[13]	pEVOL-pAzF was a gift from Peter Schultz (Addgene plasmid # 31186 ; http://n2t.net/addgene:31186 ; RRID:Addgene_31186).
pEVOL_NH2Y	[14]	The plasmid pEVOL_NH2Y was a gift from Prof. J. Stubbe (MIT).
pEVOL_pBpyAla	[15]	The plasmid pEVOL_pBpyAla was a gift from Prof. P. Schultz (The Scripps Research Institute).
pEVOL_Pyl	[16]	The pDULE-ABK was previously used to construct pEVOL_Pyl by Bart Brouwer in unpublished work pDULE-ABK was a gift from Peter Schultz (Addgene plasmid # 49086 ; http://n2t.net/addgene:49086 ; RRID:Addgene_49086).
pEVOL_PylRS(Mm)	[17]	Obtained by E.A.Lemke (EMBL).
pEVOL_PylRS_AF(Mm)		
pBAD_sfGFP151Tag	[18]	
pET17b+_LmrR	[19]	
pET17b+_LmrR_K55D_K59Q		
pET17b+_LmrR_K55D_K59Q_V15Y	[3]	
pET17b+_LmrR_K55D_K59Q_V15TAG		
pET17b+_LmrR_K55D_K59Q_N19TAG		
pET17b+_LmrR_K55D_K59Q_M89TAG		
pET17b+_LmrR_K55D_K59Q_F93TAG		
pET17b+_LmrR_K55D_K59Q_V15TAG_E7A	[20]	
pET17b+_LmrR_K55D_K59Q_V15TAG_A11L		
pET17b+_LmrR_K55D_K59Q_V15TAG_L18A		
pET17b+_LmrR_K55D_K59Q_V15TAG_N19A		

pET17b+_LmrR_K55D_K59Q_V15TAG_K22A		
pET17b+_LmrR_K55D_K59Q_V15TAG_N88A		
pET17b+_LmrR_K55D_K59Q_V15TAG_M89A		
pET17b+_LmrR_K55D_K59Q_V15TAG_A92E		
pET17b+_LmrR_K55D_K59Q_V15TAG_F93A		
pET17b+_LmrR_K55D_K59Q_V15TAG_S95A		
pET17b+_LmrR_K55D_K59Q_V15TAG_S97A		
pET17b+_LmrR_K55D_K59Q_V15TAG_D100A		

Recipe MMV

A solution of $Na_2HPO_4 \cdot 7\ H_2O$ (6.4 g), KH_2PO_4 (1.2 g) and NH_4SO_4 (1.0 g) in dH_2O (1 L) was autoclaved for 20 min at 121 °C. Then vitamin solution (1 mL of 1000x stock), trace metals solution (5 mL of 200x stock), 50% glucose solution (4 mL of 250x stock) and autoclaved $MgSO_4$ (4 mL of 1 M solution) were added before use.

**Trace metals solution (200x)**[21]	**Concentration (mg/L)** in dH_2O
Calcium nitrate · 4 H_2O	780
Iron (II) sulfate · 7 H_2O	200
Zinc sulfate · 7 H_2O	10
Boric acid	10
Cobalt chloride · 6 H_2O	10
Copper sulfate · 5 H_2O	10
Manganese sulfate · H_2O	4
Sodium molybdate · 2 H_2O	3
Nickel chloride · 6 H_2O	2
Sodium tungstate · 2 H_2O	2

**Vitamin solution (1000x)** (Modified from literature)[22]	**Concentration (mg/L)** in 50/50 EtOH/dH_2O
Thiamine · HCl	440
Pyridoxine · HCl	440
Nicotinamide	440
Pantothenic acid	440
Riboflavin	220
p-aminobenzoic acid	220
Folic acid	2.2
Biotin	2.2

Extinction coefficient of ncAAs

The ε_{280} of the ncAAs was determined by measuring their absorbances at 280 nm at various concentrations (Figure S23, Figure S24, Table S14). The UV-vis spectra were recorded on a JASCO V-660 spectrophotometer. Different concentrations were prepared by serial dilution in MOPS medium (20 mM MOPS + 150 mM NaCl, pH 5). Prior to the measurements, *p*SHF (1 mM in 20 mM MOPS, 150 mM NaCl, pH 5) was incubated with TCEP (1 mM) for 45 min at 37 °C.

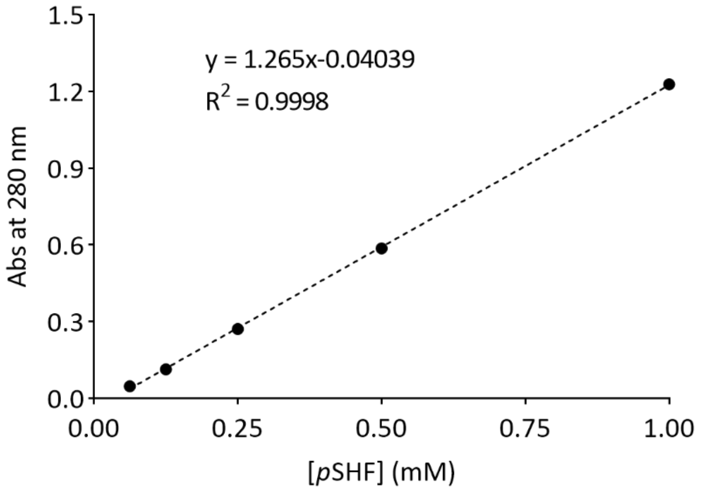

Figure S23. Determination of ε_{280} values of *p*SHF at pH 5 by plotting absorbance at 280 nm with respect to [*p*SHF].

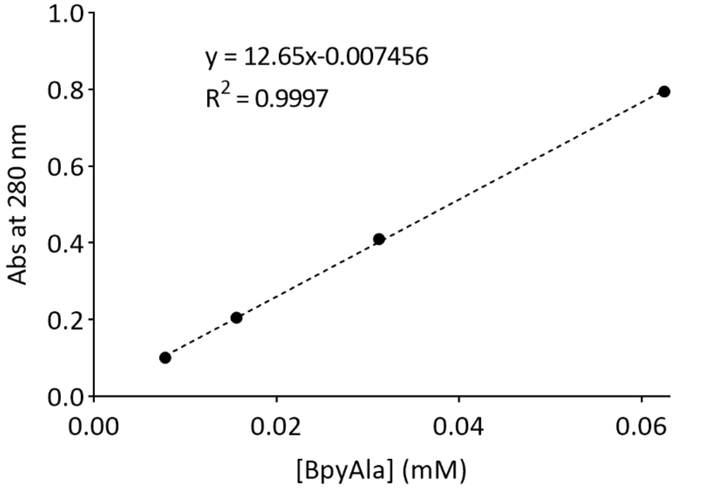

Figure S24. Determination of ε_{280} values of *p*SHF at pH 5 by plotting absorbance at 280 nm with respect to [BpyAla].

Table S14. List of non-canonical amino acids used with their respective ε_{280} values.

ncAA	$\boldsymbol{\varepsilon_{280}}$
*p*SHF (pH 5)	1265 M^{-1} cm^{-1}
BpyAla (pH 5)	12650 M^{-1} cm^{-1}
*p*AF (from literature)[3]	1333 M^{-1} cm^{-1}

Primer list

Primer name	Sequence
M8TAG_FW	ATCCCGAAAGAA**TAG**CTGCGTGCTCAA
M8TAG_RV	TTGAGCACGCAG**CTA**TTCTTTCGGGAT
A11TAG_FW	GAAATGCTGCGT**TAG**CAAACCAATGTC
A11TAG_RV	GACATTGGTTTG**CTA**ACGCAGCATTTC
K22TAG_FW	CTGAATGTGCTG**TAG**CAAGGCGATAAC
K22TAG_RV	GTTATCGCCTTG**CTA**CAGCACATTCAG
A92TAG_FW	AACATGCGCCTG**TAG**TTCGAATCCTGG
A92TAG_RV	CCAGGATTCGAA**CTA**CAGGCGCATGTT
M8A_FW	CGAAAGAA**GCG**CTGCGTGCTCAAACC
M8A_RV	CACGCAG**CGC**TTCTTTCGGGATTTCG
V15C_FW	GCTCAAACCAAT**TGC**ATCCTGCTGAAT
V15C_RV	ATTCAGCAGGAT**GCA**ATTGGTTTGAGC
A11_RV	ACGCAGCATTTCTTTCGGGATTTCGGCAC
A11C_ FW	GAAATGCTGCGT**TGC**CAAACCAATTAGATCCTG
A11D_ FW	GAAATGCTGCGT**GAT**CAAACCAATTAGATCCTG
A11E_ FW	GAAATGCTGCGT**GAA**CAAACCAATTAGATCCTG
A11F_ FW	GAAATGCTGCGT**TTC**CAAACCAATTAGATCCTG
A11G_ FW	GAAATGCTGCGT**GGT**CAAACCAATTAGATCCTG
A11H_ FW	GAAATGCTGCGT**CAC**CAAACCAATTAGATCCTG
A11I_ FW	GAAATGCTGCGT**ATC**CAAACCAATTAGATCCTG
A11K_ FW	GAAATGCTGCGT**AAA**CAAACCAATTAGATCCTG
A11M_ FW	GAAATGCTGCGT**ATG**CAAACCAATTAGATCCTG
A11N_ FW	GAAATGCTGCGT**AAC**CAAACCAATTAGATCCTG
A11P_ FW	GAAATGCTGCGT**CCG**CAAACCAATTAGATCCTG
A11Q_ FW	GAAATGCTGCGT**CAG**CAAACCAATTAGATCCTG
A11R_ FW	GAAATGCTGCGT**CGT**CAAACCAATTAGATCCTG
A11S_ FW	GAAATGCTGCGT**AGC**CAAACCAATTAGATCCTG
A11T_ FW	GAAATGCTGCGT**ACC**CAAACCAATTAGATCCTG
A11V_ FW	GAAATGCTGCGT**GTG**CAAACCAATTAGATCCTG
A11W_ FW	GAAATGCTGCGT**TGG**CAAACCAATTAGATCCTG
A11Y_ FW	GAAATGCTGCGT**TAC**CAAACCAATTAGATCCTG
M89A_FW	TGAAAAC**GCG**CGCCTGGCGTTCGAAT
M89A_RV	CCAGGCG**GCG**GTTTTCATGGCCGATT
M89X_RV and M89X_FW (except M89A)	From literature[23]

Quikchange mutagenesis

LmrR_TAG variants and LmrR_V15C were prepared using a Quikchange mutagenesis protocol as described in the literature.[24]

LmrR variants accessed by using previously prepared plasmids were transformed into chemically competent *E. coli* BL21(DE3) cells harbouring the pEVOL_pAzFRS.2.t1 plasmid.

Site-saturation libraries were prepared using the following protocol:

PCR reaction mixtures were prepared in PCR tubes according the following recipe:

Component	Amount
''master-mix''	
Template DNA	100 ng (2.86 µL)
Sterile milliQ water	133 µL
Phusion Flash master mix	200 µL
PCR-reaction mixture	
FW primer (5 µM)	1.6 µL
RV primer (5 µM)	1.6 µL
''master-mix''	16.8 µL

As a negative control, one sample contained sterile millliQ water (3.2 µL) instead of primers. The PCR tubes were shortly centrifuged, transferred to a thermocycler (Eppendorf® Mastercycler® Nexus X2) and subjected to the following PCR-protocol:

Time	Temperature
Initial denaturation	
1 min	98 °C
26 cycles of denaturation, annealing, extension	
20 s	98 °C
20 s	56 °C *
1 min	72 °C
Final extension	
5 min	72 °C

*increasing 0.3 °C per cycle

Subsequently, Dpn1 in cutsmart (2.5 µL, 10x cutsmart:Dpn1 4:1) was added to each PCR tube and incubated for 1 h at 37 °C. After which the PCR mixtures were used in the transformation protocol.

Transformation E. coli

PCR reactions (5 µL) were added to chemically competent *E. coli* NEB-10β cells (50 µL, prepared using Inoue method)[25] in a 1.5 mL microcentrifuge tube. The cells were transformed by heat shock at 42 °C for 30 sec and then placed on ice for 1 min. Then, the cells were incubated in SOC medium (900 µL) for 1 h (37 °C, 900 rpm). The tubes were centrifuged (2400 g, 1 min), the supernatant was decanted, and the cells were resuspended in the 100-200 µL that remained in the tube. Then cells (50 µL) were transferred to LB-agar plates (containing 100 µg/mL ampicillin), spread by glass beads and incubated overnight (37 °C). A single colony was picked from the plates to inoculate 5 mL LB medium cultures (containing 100 µg/mL ampicillin), which were incubated overnight (200 rpm, 37 °C). The plasmid DNA was isolated using a miniprep kit (QIAprep® Spin Miniprep Kit, Qiagen) by following the manufacturer protocol and sent for sequencing (Eurofins Genomics).

Then 5 µL of plasmid DNA was added to 50 µL of chemically competent *E. coli* BL21(DE3) cells harbouring the pEVOL_pAzFRS.2.t1 plasmid (prepared using Inoue method)[25] in a 1.5 mL microcentrifuge tube. The cells were transformed by heat shock at 42 °C for 20 sec and then placed on ice for 1 min. Then, the cells were incubated in 900 µL of SOC medium for 1 h (37 °C, 900 rpm). The tubes were centrifuged (2400 g, 1 min), the supernatant was decanted, and the cells were resuspended in the 100-200 µL that remained in the tube. Then 50 µL of the cells was transferred to LB-agar plates (containing 100 µg/mL ampicillin and 34 µg/mL chloramphenicol), spread by glass beads and incubated (37 °C, overnight). A single colony was picked from the plates to inoculate 5 mL LB medium cultures (containing 100 µg/mL ampicillin and 34 µg/mL chloramphenicol), which were incubated overnight (200 rpm, 37 °C). The cultures (500 µL) were mixed with 50% glycerol (500 µL) in cryo-tubes and stored at -70 °C.

Transformation V. natriegens

The transformation procedure was adapted from the literature.[4] One aliquot of *V. natriegens* chemically competent cells (50 µL) was taken from the -70 °C storage and placed in ice. After 10 min, approximately 1.5 µL (approximately 50 ng) of each plasmid (pET17_LmrR_V15TAG and pEVOL_pAzFRS.2.t1) was added, gently mixed, and then incubated in ice for 30 min. The cells were heat shocked in a water bath at 42 °C for 45 sec, then briefly placed back in ice. Prewarmed Vmax™ Chemicompetent Cell Recovery Media or LB + V2 salts was added to the cells (1 mL). Then the cell suspension was transferred to a sterile culture tube and incubated for 2 h (30 °C, 200 rpm). The suspension was then spun down in a sterile 1.5 mL tube (8000 g, 1 min), the supernatant was decanted, and the cells were resuspended in the 100-200 µL that remained in the tube. Then the cells (50 µL) were transferred to LB-agar plates (containing 50 µg/mL ampicillin and 12 µg/mL chloramphenicol).

Growth and expression E. coli (used for all LmrR variants)

A tube containing 5 mL LB medium (100 µg/mL ampicillin and 34 µg/mL chloramphenicol) was inoculated with a toothpick from an *E. coli* BL21(DE3) glycerol stock harbouring the plasmids pET17b_LmrR_V15TAG and pEVOL_pAzFRS.2.t1. The cells were incubated overnight (200 rpm, 37 °C). The 5 mL culture was then used to inoculate a flask containing 250 mL MMV medium (100 µg/mL ampicillin and 34 µg/mL chloramphenicol). Then, the cells were incubated (200 rpm, 37 °C) until an OD at 600 nm of 0.6-0.7 was reached. Meanwhile, a 200 mM *p*SHF + 200 mM TCEP · HCl solution in MeOH:water (1:1) with a final volume of 2.5 mL was prepared. The solution was neutralised with 10 M NaOH and incubated for 15 min at room temperature. At this point, the OTS was induced by the addition of L-arabinose (500 mg, 0.2 %w/v), and TCEP (1.25 mL of 200 mM TCEP in milliQ water pH 8, 1 mM) and the *p*SHF solution (2.5 mL, 2 mM *p*SHF) were added. The cells were incubated for 30-60 min (200 rpm, 37 °C). Then the expression of LmrR was induced with IPTG (250 µL of 1 M IPTG in milliQ water, 1 mM). The cells were incubated for 22 h (200 rpm, 24 °C). The cells were harvested by centrifugation (3000 g, 15 min, 4 °C). The supernatant was decanted, and the cell pellets were frozen at -20 °C.

Growth and expression V. natriegens
(only used for comparative analysis during the optimisation of the expression protocol)

For the growth and expression of LmrR with *V. natriegens*, we based our method on the literature that describes expression of proteins with ncAAs in *V. natriegens*.[5] Once cells with both plasmids (pET17_LmrR_V15TAG and pEVOL_pAzFRS.2.t1) were obtained, a preculture was prepared in LB + V2 salts, which was grown overnight (30 °C, 200 rpm). The next day the main culture was inoculated from the preculture (1 mL) in a flask containing 100 mL LB + V2 salts (50 µg/mL ampicillin and 12 µg/mL chloramphenicol). Then, the cells were incubated (150 rpm, 37 °C) till an OD at 600 nm of 0.6 was reached. Meanwhile, a 200 mM *p*SHF + 200 mM TCEP · HCl solution in MeOH:water (1:1) with a final volume of 1 mL was prepared. The solution was neutralised with 10 M NaOH and incubated for 15 min at 37 °C. At this point, the OTS was induced by the addition of L-arabinose (200 mg, 0.2 w/v %), TCEP (0.5 mL of 200 mM TCEP in milliQ water pH 8, 1 mM) and the *p*SHF solution (1 mL, 2 mM *p*SHF). The cells were incubated for 30-60 min (200 rpm, 37 °C). Then the expression was induced with IPTG (100 µL of 1 M IPTG in milliQ water, 1mM). The cells were incubated for 18 h (200 rpm, 30 °C). The cells were harvested by centrifugation (3000 g, 15 min, 4 °C). The supernatant was decanted, and the cell pellets were frozen at -20 °C.

Protein purification

The frozen cell pellets were resuspended in washing buffer (50 mM NaH_2PO_4, 150 mM NaCl, pH 8.0) containing 1 mM TCEP and 1 mM EDTA. The cells were lysed by sonication (70%, 10 sec on/15 sec off, 8 min), while the tube containing the cells was placed in ice-water to cool. The lysed cells were spun down by centrifugation (18500 g, 30 min, 4 °C) and loaded onto a Strep-Tactin resin. After collecting the flow-through, it was reapplied to the Strep-Tactin resin. Then the column was washed with 4 column volumes (CV) of washing buffer + 1 mM TCEP. The protein was eluted with elution buffer (washing buffer + 5 mM desthiobiotin, 1 mM TCEP) and the fractions were collected. The fractions were combined and concentrated using a 10,000 MWCO concentrator. The purity and molecular weight of the protein were assessed by ESI-HRMS.

Before use in catalysis, the protein was desalted into the reaction medium using a Sephadex G-25 size-exclusion column. The protein concentration was determined by using the calculated extinction coefficient for LmrR, corrected for the absorbance of *p*SHF ($\varepsilon_{280,\ pH\ 5}$ = 1265 $M^{-1}\ cm^{-1}$).

List of purified proteins

**Proteins**	
LmrR_WT	LmrR_V15*p*SHF_M89A
LmrR_V15*p*SHF_KK	LmrR_V15pSHF_A11L
LmrR_V15*p*SHF	LmrR_V15*p*SHF_A11I
LmrR_V15C	LmrR_V15*p*SHF_A11V
LmrR_V15Y	LmrR_V15*p*SHF_A11D
LmrR_V15*p*AF	LmrR_V15*p*SHF_A11E
LmrR_BpyAla	LmrR_V15*p*SHF_A11W
LmrR_A11*p*SHF	

DNA/Amino acid sequence LmrR_V15pSHF and LmrR_V15pSHF_A11L

LmrR_V15pSHF

GGTGCCGAAATCCCGAAAGAAATGCTGCGTGCTCAAACCAAT**TAG**ATCCTGCTGAATGTCCTGAAACAAGGCGATA
ACTATGTGTATGGCATTATCAAACAGGTGAAAGAAGCGAGCAACGGTGAAATGGAACTGAATGAAGCCACCCTGTA
TACGATTTTTGATCGTCTGGAACAGGACGGCATTATCAGCTCTTACTGGGGTGATGAAAGTCAAGGCGGTCGTCGC
AAATATTACCGTCTGACCGAAATCGGCCATGAAAACATGCGCCTGGCGTTCGAATCCTGGAGTCGTGTGGACAAAA
TCATTGAAAATCTGGAAGCAAACAAAAAATCTGAAGCGATCAAATCTAGAGGTGGCAGCGGTGGCTGGAGCCACCC
GCAGTTCGAAAAATAA

GAEIPKEMLRAQTN**(*p*SHF)**ILLNVLKQGDNYVYGIIKQVKEASNGEMELNEATLYTIFDRLEQDGIISSYWGDESQ
GGRRKYYRLTEIGHENMRLAFESWSRVDKIIENLEANKKSEAIKSRGGSGGWSHPQFEK*

LmrR_V15pSHF_A11L

GGTGCCGAAATCCCGAAAGAAATGCTGCGT**CTG**CAAACCAAT**TAG**ATCCTGCTGAATGTCCTGAAACAAGGCGATA
ACTATGTGTATGGCATTATCAAACAGGTGAAAGAAGCGAGCAACGGTGAAATGGAACTGAATGAAGCCACCCTGTA
TACGATTTTTGATCGTCTGGAACAGGACGGCATTATCAGCTCTTACTGGGGTGATGAAAGTCAAGGCGGTCGTCGC
AAATATTACCGTCTGACCGAAATCGGCCATGAAAACATGCGCCTGGCGTTCGAATCCTGGAGTCGTGTGGACAAAA
TCATTGAAAATCTGGAAGCAAACAAAAAATCTGAAGCGATCAAATCTAGAGGTGGCAGCGGTGGCTGGAGCCACCC
GCAGTTCGAAAAATAA

GAEIPKEMLR**L**QTN**(*p*SHF)**ILLNVLKQGDNYVYGIIKQVKEASNGEMELNEATLYTIFDRLEQDGIISSYWGDESQ
GGRRKYYRLTEIGHENMRLAFESWSRVDKIIENLEANKKSEAIKSRGGSGGWSHPQFEK*

SI.23 Library preparation and screening procedures

LmrR variants were expressed in tubes containing 5 mL MMV medium using the described expression protocol for *E. coli* (SI.23).

The cell pellets were resuspended in 500 µL lysis medium (20 mM MOPS, 150 mM NaCl, 1 mg/mL lysozyme (from chicken egg white), 0.1 mg/mL DNaseI, 10 mM $MgSO_4$, pH 5). The OD at 600 nm was measured (1:10 dilution with milliQ water). The tubes were incubated for 2 h (200 rpm, 30 °C). The lysed cells were transferred to 1.5 mL microcentrifuge tubes and spun down (17000 g, 5 min). The OD-normalised lysates (lowest OD = 140 µL) were mixed with 200 µL (100 µL for M89X-library) bead solution (Strep-Tactin Superflow high capacity, 50% suspension). 600 µL of phosphate buffer (50 mM NaH_2PO_4, 150 mM NaCl, pH 8.0) was added and the mixture was vortexed (1 min), incubated at room temperature (4 min) and centrifuged (17000 g, 2 min). The supernatant was discarded and the beads were resuspended in 600 µL MOPS buffer (20 mM MOPS, 150 mM NaCl, pH 7) and centrifuged (17000 g, 2 min). The supernatant was discarded and the beads were resuspended in 600 µL MOPS medium (20 mM MOPS, 150 mM NaCl, pH 5) and centrifuged (17000 g, 2 min). The supernatant was fully discarded and the beads were resuspended in 200 µL (300 µL for M89X-library) MOPS medium (20 mM MOPS, 150 mM NaCl, pH 5).

The bead solutions (140 µL per well, duplicates) were added to a 96-well microplate (Nunc F96 MicroWell, black, polystyrene, ThermoFisher). Then a freshly prepared stock of $Au(SMe_2)Cl$ in MeCN (5 µL, 300 µM stock, 10 µM final concentration) was added to each well and incubated at room temperature (5 min). Finally, a freshly prepared stock of substrate **6a** in MeCN (5 µL, 30 mM stock, 1 mM final concentration) was added to each well. Kinetic measurements were performed in a microplate reader (Synergy H1, BioTek) at 37 °C with double orbital shaking by measuring relative fluorescence intensity ($\lambda_{excitation}$= 320 nm and $\lambda_{emission}$ = 365 nm) over time. Based on the obtained time course, the RFU of different LmrR variants was compared at time points before saturation was reached.

SI.24 Mass spectrometry procedures

Calculated masses

Theoretical masses of the most abundant isotope of the proteins were calculated by extracting the molecular formula of the proteins aided by the expasy webserver: https://web.expasy.org/protparam/. An isotope simulation was performed on the molecular formula using Thermo Scientific FreeStyle – 1.8 with a profile resolution of 100,000. The highest intensity isotope from this simulation was used as M_{calc} for purified proteins.

In some cases, two isotopes were close in intensity in both the calculated and observed mass and the highest intensity isotopes were not aligned. Therefore, a deconvolution was performed using Thermo Scientific FreeStyle – 1.8 to obtain the monoisotopic mass of the recorded m/z spectrum. If this matched with the calculated monoisotopic mass, the calculated most abundant isotope was aligned with the same isotope in the recorded mass spectrum.

LC/LRMS method

Samples were measured using a WatersTM Acquity Ultra Performance LC coupled to a WatersTM Xevo G2 QTOF. The samples were injected on a ACQUITY UPLC BEH300 C4 1.7 µm column using water and MeCN with 0.1 %v/v formic acid as mobile phase with a flow rate of 0.3 mL/min using the following method.

Retention time (min)	Water (%)	MeCN (%)
0.00	90	10
2.00	90	10
10.00	50	50
11.00	5	95
13.00	5	95
13.10	90	10
17.00	90	10

Apo proteins

Identity confirmation of purified proteins was performed by LC/HRMS on a Vanquish™ core coupled to an Orbitrap Exploris™ 480. Samples were prepared in 20 µM dimer concentration in buffer: MOPS medium (pH 5, 20 mM MOPS + 150 mM NaCl) or NaH_2PO_4 (pH 8, 50 mM NaH_2PO_4 + 150 mM NaCl). The samples were separated with a bioZen™ 3.6 µm Intact XB-C8 column using water and MeCN with 0.1 %v/v formic acid as mobile phase with a flow rate of 0.3 mL/min using the following method. MS spectra were recorded with a scan parameter of 480,000 and a scan range of 200-2000.

Retention time (min)	water (%)	MeCN (%)
0.00	95	5
2.00	95	5
12.00	5	95
14.00	5	95
14.10	95	5

[Au]-bound proteins

Samples were prepared with 20 µM LmrR dimer concentration in 50 mM NH_4HCO_3 (pH 5). To this, 40 µM Au(SMe_2)Cl was added with 5 %v/v MeCN. The samples were incubated for 15-30 min at room temperature and then injected via a bypass column into an Orbitrap Exploris^{TM} 480 using a constant flow of 50:50 water:MeCN with 0.1 %v/v formic acid at a rate of 0.3 mL/min. MS spectra were recorded with a scan parameter of 240,000 and a scan range of 200-2000.

Identification modifications: oxidation

A 5 µM LmrR_V15*p*SHF (dimer) sample in NaH_2PO_4 (pH 8, 50 mM NaH_2PO_4 + 150 mM NaCl) buffer was incubated with 20 mM TCEP at room temperature while shaking. LCMS spectra were recorded at t = 0 h, 6 h and 26 h using the LC/LRMS method.

Trypsin digest

The protein solutions were denatured with 2 M urea and reduced with 10 mM TCEP for 1 h at 37 °C and then alkylated with 15 mM iodoacetamide at room temperature in the dark for 45 min. The samples were diluted with 100 mM NH_4HCO_3 until an urea concentration of 1 M was reached. The samples were digested by 1/50 (w/w) sequencing grade modified trypsin at 37 °C overnight. After digestion, TFA was added to reach 1 %v/v final concentration and the digested protein was subsequently purified by solid phase extraction.

Digested protein samples to identify methylation on the *p*SHF residue were measured on a LTQ Orbitrap XL (MS1 resolution = 60,000, MS/MS is in iontrap). Data was analysed on PEAKS studio X 10.6 with an MS1 error tolerance of 20 ppm and a fragment error tolerance of 0.5 Da.

Digested protein samples to confirm the purity after expression and purification optimisation were measured on an Exploris 480 (MS1 resolution = 120,000, MS/MS resolution of 15,000). Data was analysed on PEAKS studio 11 with a MS1 error tolerance of 10 ppm and a fragment error tolerance of 0.02 Da.

Metal-binding studies

The respective protein solutions (20 µM) were prepared in 50 mM NH_4HCO_3 (pH 7.8) and incubated with the respective metal complex (40 µM of metal) with 0-2 %v/v MeCN at room temperature. After 30-60 min and 180-240 min of incubation the samples were injected into a WatersTM Xevo G2 QTOF via a bypass column with a constant flow of 90:10 water:MeCN with 0.1 %v/v formic acid at a rate of 0.3 mL/min. After 60-180 min the samples were characterised using the LC/LRMS method.

The TIC peak in the obtained chromatogram was extracted to get the m/z spectrum. This was deconvoluted in MagTran using a mass range of 10,000-80,000, a charge range of 1-80, a S/N threshold of 10 and a max no. of species of 10. Only if the apo protein peak was not visible under these parameters, the S/N threshold was lowered to 5. The centroid of the peaks was determined by using the Gaussian deconvolution function of MagTran on the peak of interest. The resulting mass domain was integrated in specific ranges by taking into account the calculated masses of the respective proteins and metal complexes. The peak areas containing [protein+metal] were divided by the peak areas containing protein without metal to obtain a ratio that is representative of the extent of metal adduct formation under MS conditions.

SI.25 HR-mass spectra of purified proteins and Au-bound proteins

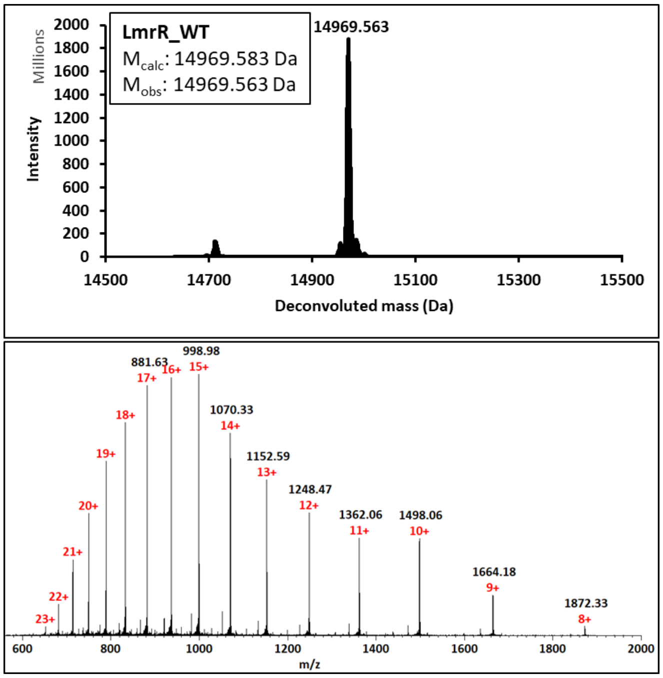

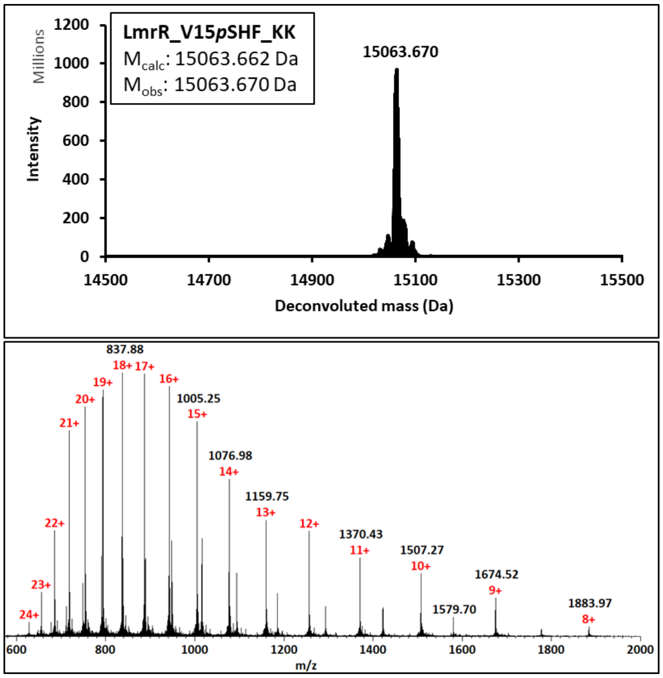

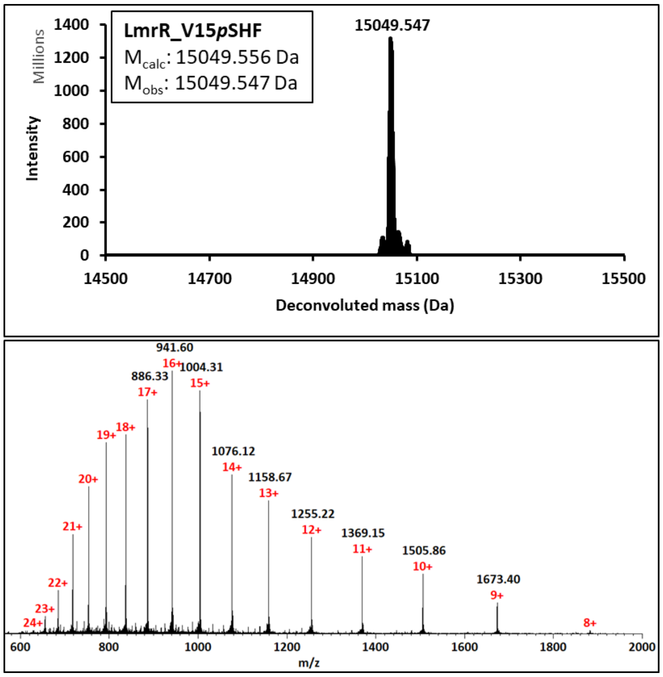

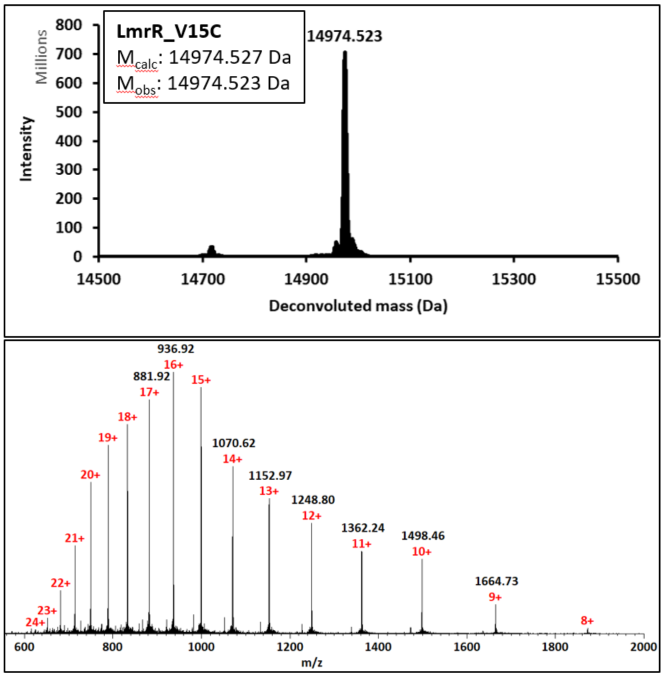

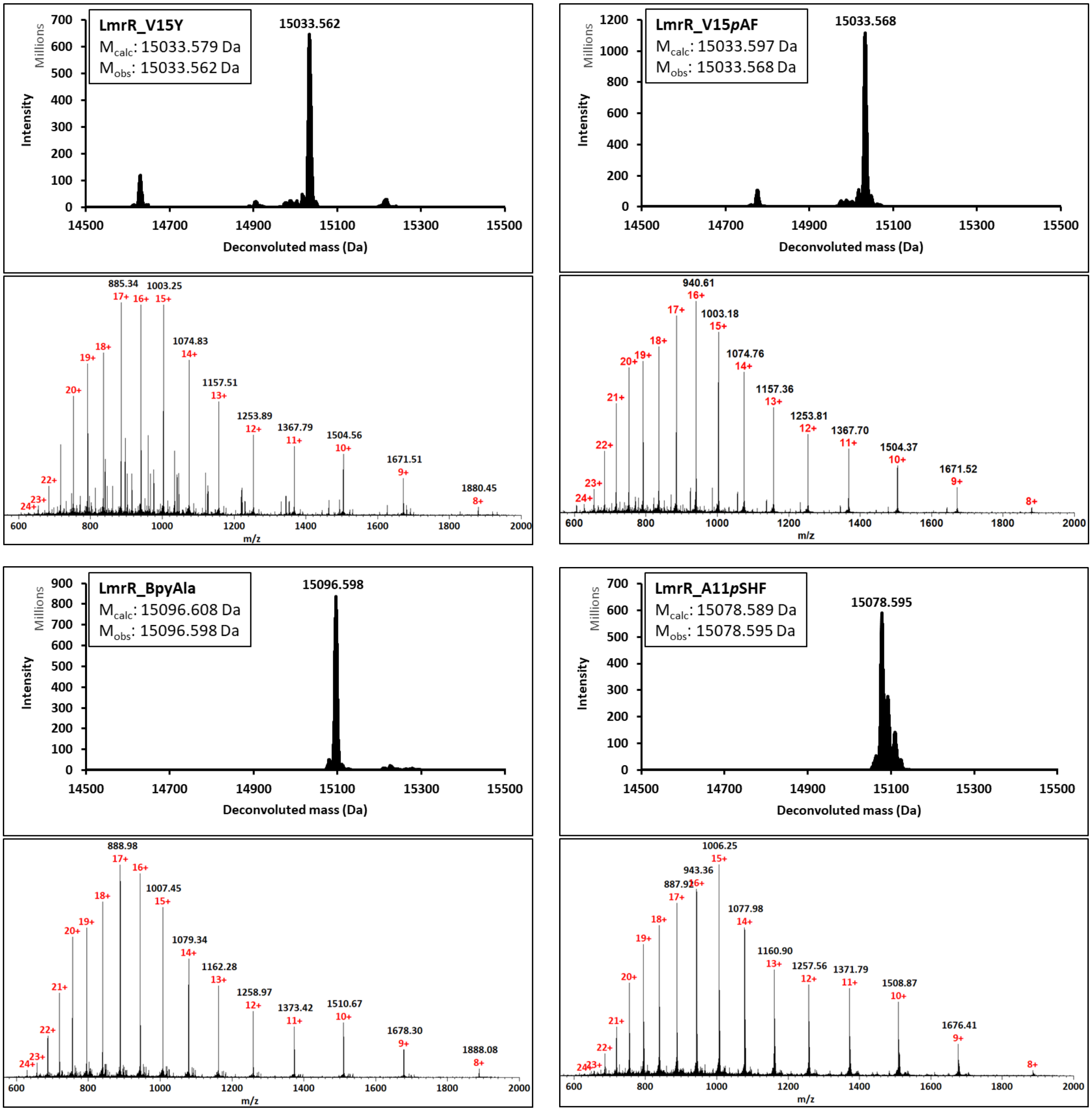
LmrR_V15Y
M$_{calc}$: 15033.579 Da
M$_{obs}$: 15033.562 Da
15033.562
Intensity
Millions
Deconvoluted mass (Da)
885.34 17+
1003.25 16+ 15+
18+
19+
1074.83 14+
20+
1157.51 13+
1253.89 12+
1367.79 11+
1504.56 10+
1671.51 9+
1880.45 8+
22+
23+
24+
m/z
LmrR_V15pAF
M$_{calc}$: 15033.597 Da
M$_{obs}$: 15033.568 Da
15033.568
Intensity
Millions
Deconvoluted mass (Da)
940.61 16+
17+
1003.18 15+
18+
20+ 19+
1074.76 14+
21+
1157.36 13+
1253.81 12+
1367.70 11+
22+
1504.37 10+
1671.52 9+
23+
24+
8+
m/z
LmrR_BpyAla
M$_{calc}$: 15096.608 Da
M$_{obs}$: 15096.598 Da
15096.598
Intensity
Millions
Deconvoluted mass (Da)
888.98 17+
16+
18+
1007.45 15+
19+
20+
1079.34 14+
1162.28 13+
21+
1258.97 12+
1373.42 11+
1510.67 10+
22+
1678.30 9+
1888.08 8+
23+
24+
m/z
LmrR_A11pSHF
M$_{calc}$: 15078.589 Da
M$_{obs}$: 15078.595 Da
15078.595
Intensity
Millions
Deconvoluted mass (Da)
1006.25 15+
943.36
887.92 16+
17+
1077.98 14+
18+
19+
1160.90 13+
1257.56 12+
1371.79 11+
1508.87 10+
20+
21+
1676.41 9+
22+
24 23+
8+
m/z

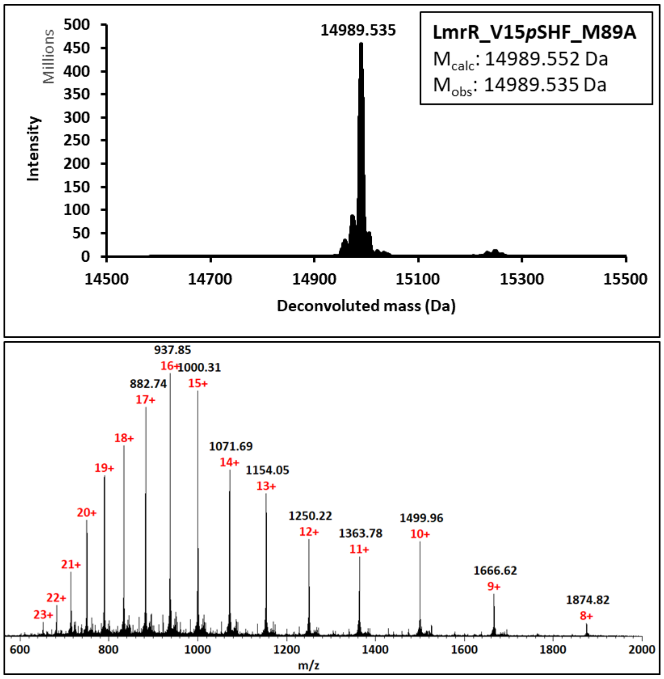
LmrR_V15pSHF_M89A
Mcalc: 14989.552 Da
Mobs: 14989.535 Da
14989.535
Intensity
Millions
Deconvoluted mass (Da)
937.85
1000.31
882.74
1071.69
1154.05
1250.22
1363.78
1499.96
1666.62
1874.82
m/z

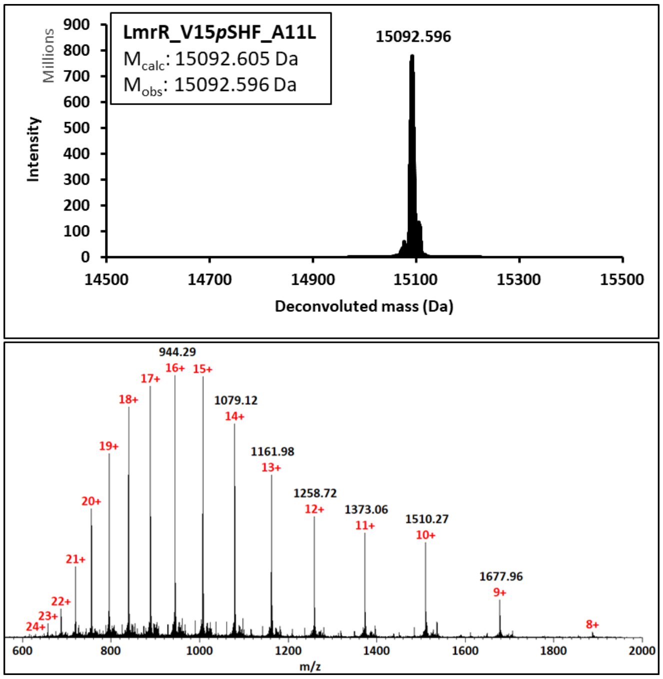
LmrR_V15pSHF_A11L
Mcalc: 15092.605 Da
Mobs: 15092.596 Da
15092.596
Intensity
Millions
Deconvoluted mass (Da)
944.29
1079.12
1161.98
1258.72
1373.06
1510.27
1677.96
m/z

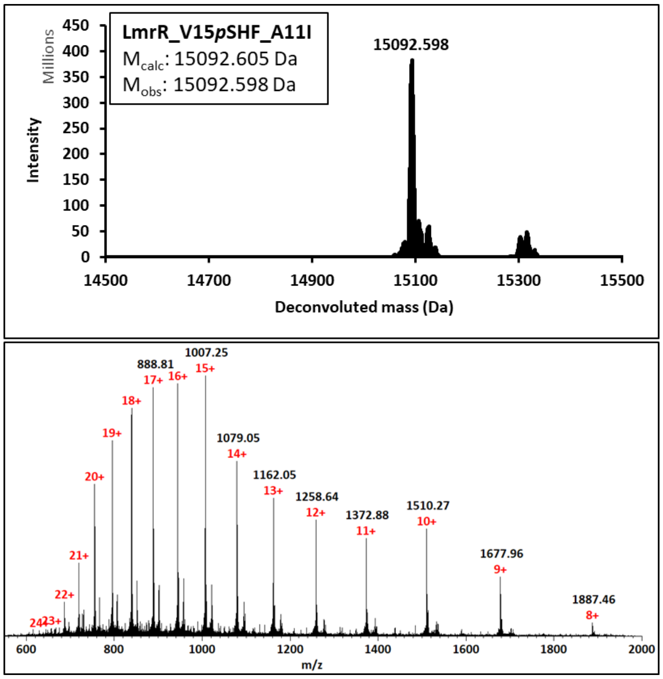
LmrR_V15pSHF_A11I
Mcalc: 15092.605 Da
Mobs: 15092.598 Da
15092.598
Intensity
Millions
Deconvoluted mass (Da)
1007.25
888.81
1079.05
1162.05
1258.64
1372.88
1510.27
1677.96
1887.46
m/z

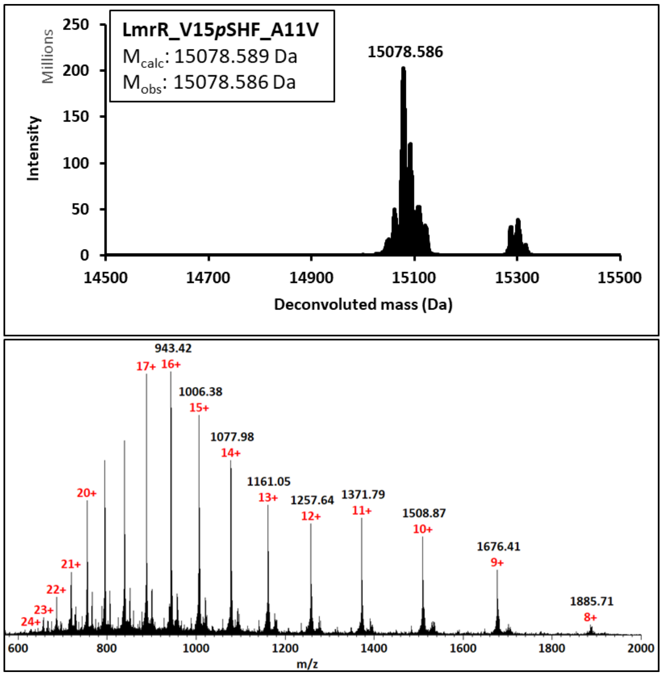
LmrR_V15pSHF_A11V
Mcalc: 15078.589 Da
Mobs: 15078.586 Da
15078.586
Intensity
Millions
Deconvoluted mass (Da)
943.42
1006.38
1077.98
1161.05
1257.64
1371.79
1508.87
1676.41
1885.71
m/z

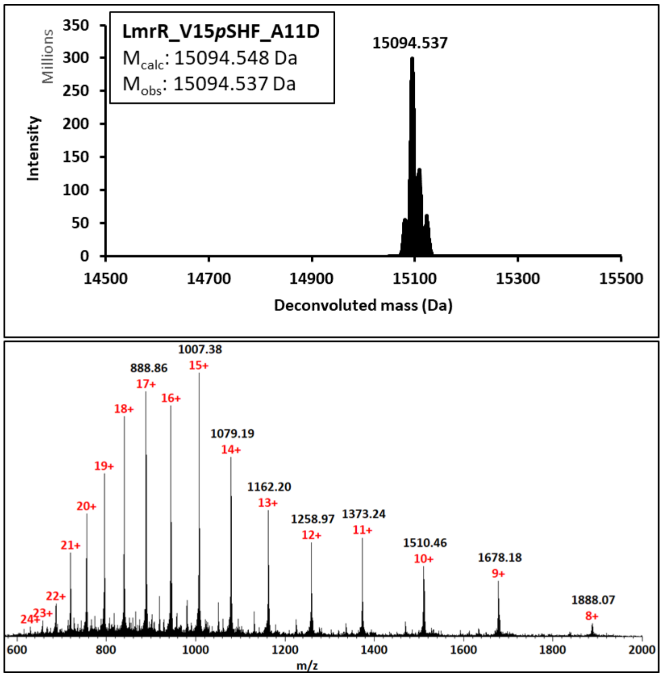
LmrR_V15pSHF_A11D
M_{calc}: 15094.548 Da
M_{obs}: 15094.537 Da
15094.537
Intensity
Millions
Deconvoluted mass (Da)
1007.38
15+
888.86
17+
16+
18+
1079.19
14+
19+
1162.20
13+
20+
1258.97
12+
1373.24
11+
1510.46
10+
21+
1678.18
9+
1888.07
8+
22+
24+
23+
m/z

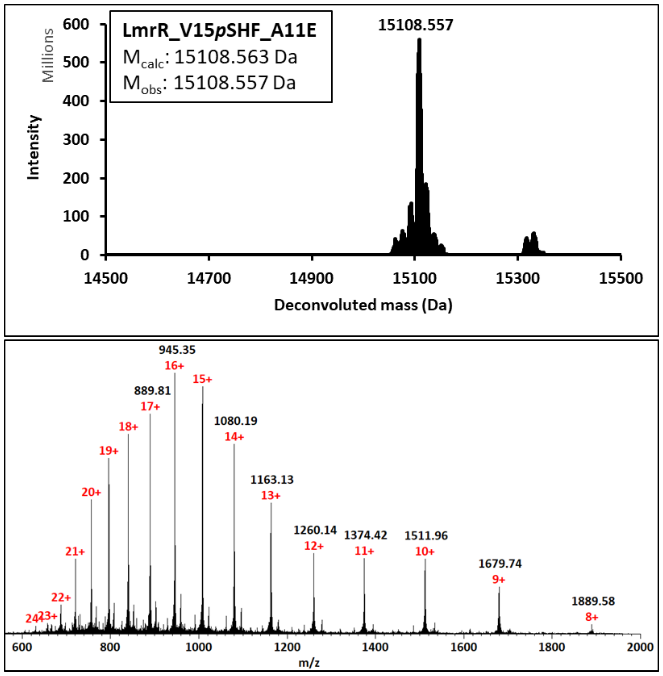
LmrR_V15pSHF_A11E
M_{calc}: 15108.563 Da
M_{obs}: 15108.557 Da
15108.557
Intensity
Millions
Deconvoluted mass (Da)
945.35
16+
15+
889.81
17+
18+
1080.19
14+
19+
1163.13
13+
20+
1260.14
12+
1374.42
11+
1511.96
10+
21+
1679.74
9+
22+
1889.58
8+
24+
23+
m/z

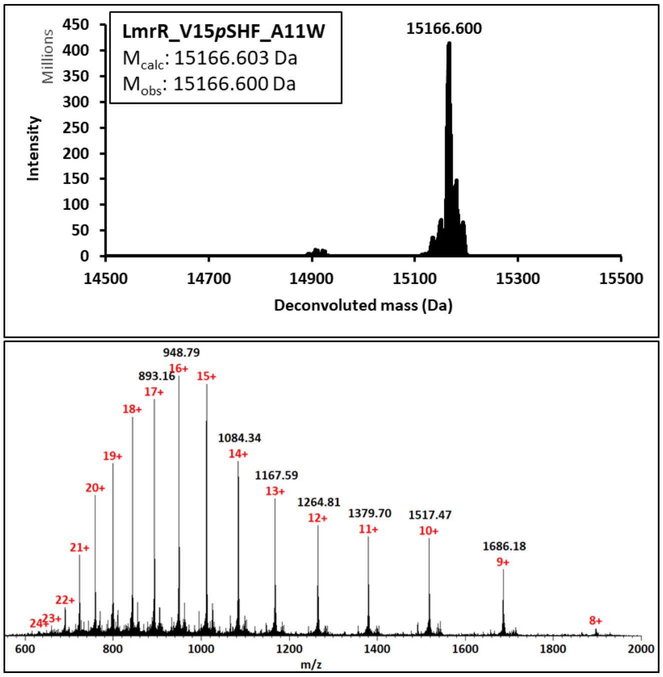
LmrR_V15pSHF_A11W
M_{calc}: 15166.603 Da
M_{obs}: 15166.600 Da
15166.600
Intensity
Millions
Deconvoluted mass (Da)
948.79
893.16
16+
15+
17+
18+
1084.34
14+
19+
1167.59
13+
20+
1264.81
12+
1379.70
11+
1517.47
10+
1686.18
9+
21+
22+
23+
24+
8+
m/z

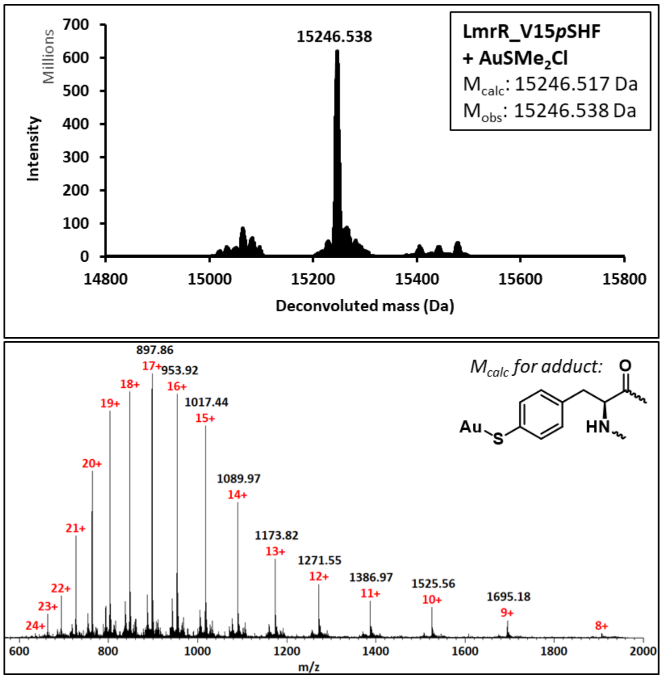
LmrR_V15pSHF
+ AuSMe2Cl
Mcalc: 15246.517 Da
Mobs: 15246.538 Da
15246.538
Intensity
Millions
Deconvoluted mass (Da)
Mcalc for adduct:
Au
S
HN
O
m/z

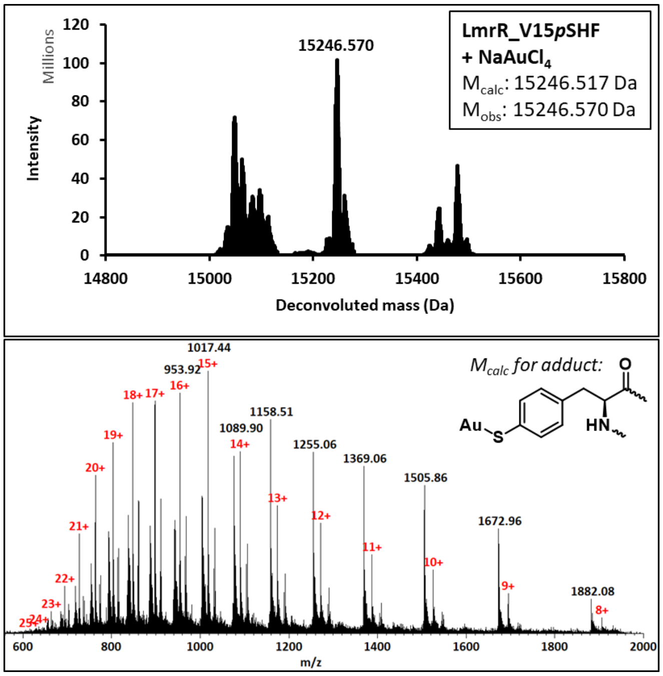
LmrR_V15pSHF
+ NaAuCl4
Mcalc: 15246.517 Da
Mobs: 15246.570 Da
15246.570
Intensity
Millions
Deconvoluted mass (Da)
Mcalc for adduct:
Au
S
HN
O
m/z

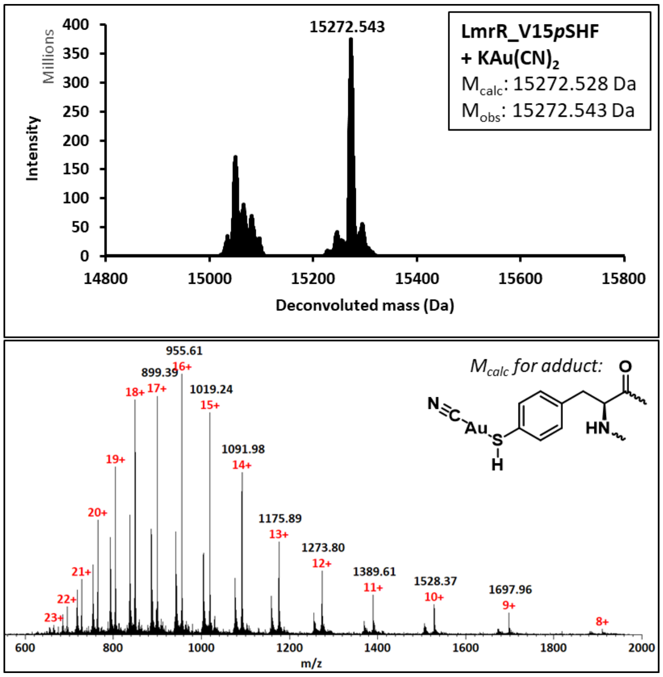
LmrR_V15pSHF
+ KAu(CN)2
Mcalc: 15272.528 Da
Mobs: 15272.543 Da
15272.543
Intensity
Millions
Deconvoluted mass (Da)
Mcalc for adduct:
N
C
Au
S
H
HN
O
m/z

SI.26 X-ray crystallography procedures

Expression and purification of LmrR_V15pSHF_KK

LmrR_V15*p*SHF containing wild-type DNA-binding residues K55 and K59 (LmrR_V15pSHF_KK) was produced for crystallisation purposes (re-introduction of the DNA-binding lysines facilitates the growth of ordered crystals). LmrR_V15pSHF_KK was expressed using the described expression protocol for *E. coli* (SI.23).

The frozen cell pellets were resuspended in washing buffer (50 mM NaH_2PO_4, pH 8). The cells were lysed by sonication (70%, 10 sec on/15 sec off, 5 min). After lysis, TCEP (4 mM final concentration), EDTA (1 mM final concentration), 0.5 µL of DENERASE (>250 U/µL) and $MgCl_2$ (20 mM final concentration) were added. The lysed cells were spun down by centrifugation (18500 g, 60 min, 4 °C) and the supernatant loaded onto a Strep-Tactin column (Strep-Tactin Superflow high capacity), equilibrated in 50 mM NaH_2PO_4, pH 8. After collecting the flow-through, it was reapplied to the Strep-Tactin resin. Then the column was washed with 4 CVs of washing buffer + 1 mM TCEP. The protein was eluted with elution buffer (washing buffer + 5 mM desthiobiotin, 1 mM TCEP) and the fractions were collected. The protein-containing fractions were combined and concentrated using a 10,000 MWCO concentrator. Subsequently, DNA was removed from the protein sample using a Heparin column (Cytiva), pre-equilibrated with 50 mM NaH_2PO_4, pH 8. The concentrated protein solution was added to the Heparin column and the column was washed with 1 CV of 50 mM NaH_2PO_4, pH 8. Then, a linear gradient of 0-1 M NaCl concentration was applied, and the protein eluted around 1 M NaCl. The protein fractions were pooled and cooled on ice overnight.

Crystallisation of apo LmrR_V15pSHF_KK

Prior to crystallisation, purified LmrR_V15*p*SHF_KK was polished via size-exclusion chromatography with a Superdex 200 Increase 10/300 GL column (Cytiva), using 25 mM Tris-HCl, pH 7.5, 150 mM NaCl and 0.5 mM TCEP as running buffer, and concentrated to 14 mg/ml. Crystallisation conditions were identified using the sitting drop vapour diffusion technique, with the help of a Mosquito dispensing robot (SPTLabTech). Drops of 200 nL were dispensed at two protein:reservoir volume ratios (1.25:0.75 and 0.75:1.25) using reservoir solutions containing 16-21% (w/v) PEG 3350, 0.2 M KSCN in 0.2 M Bis-Tris propane, pH 6.5-7.2. Crystals grew within a few days at 21 °C. As a preparation for X-ray data collection, crystals were transferred to a drop of mother liquor supplemented with 25% glycerol, immediately followed by flash-cooling in liquid nitrogen.

Co-crystallisation of LmrR_V15pSHF_KK with $KAuCN_2$

To obtain protein with bound Au(I) ions, purified LmrR_V15*p*SHF_KK was first applied to a Superdex 200 Increase 10/300 GL column (Cytiva), equilibrated with 20 mM MOPS, 150 mM NaCl, pH 7. The protein eluted after roughly 11 minutes, which matches the previously reported elution time of dimeric LmrR variants.[3] The protein solution was concentrated and the concentration determined by diluting a defined aliquot in 0.2 M acetate buffer (pH 5) and measuring the absorption at 280 nm. Next, $KAuCN_2$ was added (10 mM stock in 20 mM MOPS, 150 mM NaCl, pH 7) such that the final concentrations of $KAuCN_2$ and protein were 824 µM and 412 µM, respectively. The mixture was incubated at room temperature (10 min) and then stored in ice untill crystallisation drops were set up. Crystallisation was performed as for the apo protein, using the PACT Premier screen (Molecular Dimensions) for providing the reservoir solutions. Crystals grew at various conditions, with the best crystals obtained with 20% (w/v) PEG 3350, 0.2 M NaF in 0.1 M Bis-Tris propane, pH 7.5. The crystals were fished, sequentially transferred into a droplet of cryoprotecting solution (crystallisation solution with 25% glycerol) and flash-cooled in liquid nitrogen.

X-ray data collection and crystal structure determinations

X-ray diffraction data were collected at 100 K at the ID30A-1 (MASSIF-1) beamline of the European Synchrotron Radiation Facility (ESRF) in Grenoble, France. Initial data processing was performed using the automatic XDSAPP data processing pipeline[26] implemented at the synchrotron. Starting from the unmerged data, merging and reduction to unique structure factors was carried out using the AIMLESS[27] task from the CCP4 software suite (version 8.0)[28]. Crystal structures of apo and Au(I)-bound LmrR_V15*p*SHF_KK were determined at 2.35 Å and 2.50 Å resolution, respectively. Both crystal structures contain one LmrR dimer in the asymmetric unit. Initial phases and structures were determined by molecular replacement with PHASER[29] using a subunit of a previously published structure of LmrR (PDB entry 6I8N)[30] as a search model. The structures were subsequently improved via rounds of manual model building using Coot[31], alternated with restrained refinement using REFMAC[32]. Geometry restraints for the *p*SHF residue were generated using AceDRG[33]. The presence and position of the gold atom were identified by searching for peaks in anomalous and Fo-Fc difference Fourier maps. Large overall atomic B-factors hampered refinement of the structures, resulting in crystallographic R-factors that are somewhat larger as expected, compared to crystal structures in the PDB determined at similar resolutions. The geometries of the final structures were validated with MolProbity[34]. A summary of the data collection and refinement statistics is available in Table S15. Coordinates and structure factors have been deposited in the Protein Data Bank with entry codes 9G51 and 9G52 for the apo and Au-bound structure, respectively.

Table S15. LmrR_V15*p*SHF_KK crystallographic data collection and refinement statistics. Values in parentheses refer to the highest resolution shell.· The R_{free} is calculated as the R-factor, using 5% of all reflections randomly chosen which were excluded from structure refinement.

	apo	Au(I)-bound
**Data collection**		
Wavelength (Å)	0.96546	0.96546
Resolution range (Å)	68 – 2.35<br>(2.44 – 2.35)	49 – 2.50<br>(2.60 – 2.50)
Space group	C 2	P 2_1 2_1 2_1
Unit cell, a, b, c (Å)	102.9, 35.0, 68.1<br>β = 97.2°	35.4, 52.7, 146.2
R_{meas}	0.050 (1.447)	0.127 (1.427)
R_{pim}	0.024 (0.677)	0.041 (0.482)
Mean(I/σI)	13.3 (1.0)	11.1 (1.6)
$CC_{1/2}$	1.000 (0.586)	0.998 (0.726)
Multiplicity	4.4 (4.4)	9.4 (8.6)
Completeness (%)	100.0 (100.0)	100.0 (100.0)
Wilson B-factor (Å^2)	67.1	50.1
**Refinement**		
Content asymmetric unit	1 protein dimer,<br>1 water	1 protein dimer,<br>1 Au(I), 1 OH^-
R-factor, R_{free}	0.217, 0.262	0.245, 0.294
Average B-factors (Å^2)<br>protein, solvent, other	97.9, 46.6, 84.3	74.8, 46.4, 76.5
RMSD,<br>bond lengths (Å)<br>bond angles (°)	0.009<br>2.0	0.007<br>1.8
Ramachandran,<br>favoured (%)<br>outliers (%)	99.0<br>0.0	97.1<br>0.0
Rotamer outliers (%)	1.6	3.6
Molprobity score	1.40	1.96
PDB entry	9G51	9G52

SI.27 Spectroscopy procedures (UV-vis, CD)

UV-vis [Au]-titration

UV-vis spectra were recorded on a SPECORD 210 PLUS spectrophotometer at room temperature in a 1 cm pathlength quartz cuvette. Samples with 20 µM LmrR variant or 40 µM thiophenol (3.33 µL, 6 mM stock in MeCN) were prepared in MOPS medium (20 mM MOPS, 150 mM NaCl, pH 5) with a total volume of 500 µL. To this was titrated Au(SMe_2)Cl in MeCN (8 x 1.25 µL, 4 mM stock, 10 µM increments). Addition of each aliquot was followed by incubation for 1-2 min at room temperature before recording the UV-vis spectrum (scan range 200-800 nm, 20 nm/s). Absorbances were corrected for dilution.

CD spectroscopy

LmrR_V15*p*SHF samples were prepared in 4 µM dimer protein concentration in 50 mM Na_2HPO_4 buffer at pH 5 with 2.6 %v/v MeCN either with or without 8 µM Au(SMe_2)Cl. The samples were incubated for 15 min at ambient temperature. CD spectra were recorded on a JASCO J-810 using 1 mm pathlength quartz cuvettes with a 2 nm bandwidth and 1 nm increments.

SI.28 Catalysis and kinetic experiments procedures

Reaction set-up hydroamination with different metal complexes

A microcentrifuge tube (1.5 mL) was charged with freshly desalted LmrR_V15*p*SHF (21 µM final dimer concentration) in phosphate buffer (100 mM Na_2HPO_4, pH 5). The respective metal complex in MeCN was added (2.67 µL, 2.25 mM stock, 40 µM final concentration) to the solution and incubated for 5 min at room temperature. Then substrate **1a** in MeCN (2.5 µL, 60 mM stock, 1 mM final concentration) was added to the solution. The reaction mixture (150 µL) was shaken for 16 h (850 rpm, 37 °C). Samples were prepared for GC-FID analysis.

Reaction set-up hydroamination with Au(SMe₂)Cl

A microcentrifuge tube (1.5 mL) was charged with freshly desalted protein (10 µM final dimer concentration) in MOPS medium (20 mM MOPS, 150 mM NaCl, pH 5). Then a freshly prepared stock of $Au(SMe_2)Cl$ in MeCN (1.33 µL, 2.25 mM stock, 20 µM final concentration) was added to the solution. After 5 min of incubation at room temperature, the substrate in MeCN (2.5 µL, 60 mM stock, 1 mM final concentration) was added. The reaction mixture (150 µL) was shaken for 16 h (850 rpm, 37 °C). Samples were prepared for GC-FID or SFC analysis.

Reaction set-up hydroamination with $Au(SMe_2)Cl$ (substrate 3)

A microcentrifuge tube (1.5 mL) was charged with freshly desalted protein (20 µM final dimer concentration) in MOPS medium (20 mM MOPS, 150 mM NaCl, pH 5). Then a freshly prepared stock of $Au(SMe_2)Cl$ in MeCN (varying concentrations depending on [Au] equiv.) was added to the solution. MeCN was added appropriately to get a final concentration of 3.3 %v/v MeCN. After 5 min of incubation at room temperature, substrate **3** in MeCN (2.5 µL, 60 mM stock, 1 mM final concentration) was added. The reaction mixture (150 µL) was shaken for 16 h (850 rpm, 37 °C). Samples were prepared for SFC analysis.

Kinetics and time course experiments

A microcentrifuge tube (1.5 mL) was charged with a mixture of 5 µM freshly desalted LmrR variant, 10 µM $Au(SMe_2)Cl$ and MeCN (final concentration 10 %v/v) in MOPS medium (20 mM MOPS, 150 mM NaCl, pH 5). After 5 min of incubation at room temperature, substrate **6a** in MeCN (varying concentrations, for time course: 200 µM final concentration) was added to reach a total volume of 150 µL. The reaction mixture (150 µL) was incubated for 30 min (850 rpm, 37 °C) (for time course: varying incubation times). Then the tubes were quenched with TCEP (0.6 µL, 50 mM stock in phosphate buffer pH 8, 200 µM final concentration) and cooled on ice. Samples were prepared for SFC analysis.

Work-up for GC-FID analysis

To the reaction mixture, 300 µL DCM with 250 µM mesitylene as internal standard was added. The sample was vortexed for 1 min and inverted (x3). The layers were separated by centrifugation (13000 g, 2 min). 250 µL of the organic layer was pipetted into a GC-vial. The samples were analysed on a Shimadzu GC-2014 with a flame ionisation detector.

Work-up for SFC analysis

The tube was centrifuged (13000 g, 30 sec) and 300 µL DCM with 2-phenylquinoline (250 µM or 50 µM for time course and kinetics) as internal standard was added. The sample was vortexed (1 min) and inverted (x3). The layers were separated by centrifugation (13000 g, 2 min). 290 µL of the organic layer was pipetted into a new microcentrifuge tube (1.5 mL) and the solvent was removed *in vacuo* (15 min, 30 °C). The residue was redissolved in SFC grade MeOH (80 µL). The samples were analysed on a WatersTM Acquity UPC2 system.

SI.29 Synthetic chemistry (synthesis and NMR)

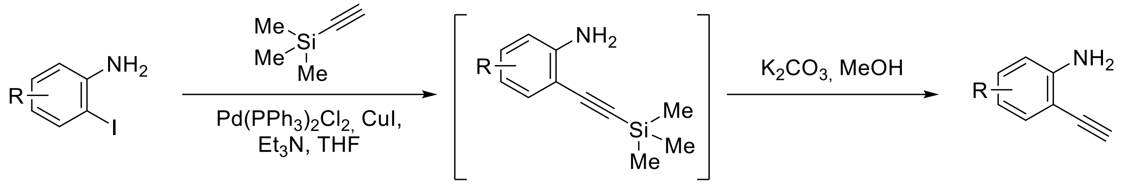

Scheme S3 two-step synthesis towards 2-ethynyl anilines

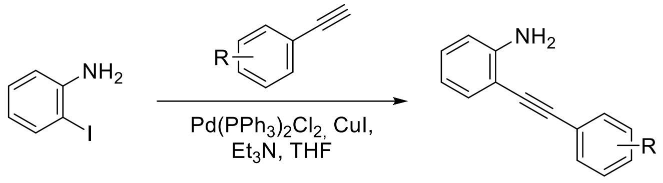

Scheme S4 synthesis towards 2-(arylethynyl)anilines

General synthetic procedure towards 2-ethynyl anilines (GP1)[35]

The 2-iodoaniline substrate (2.5 mmol, 1 eq.), $Pd(PPh_3)_2Cl_2$ (88 mg, 0.13 mmol, 0.05 eq.), CuI (24 mg, 0.13 mmol, 0.05 eq.), Et_3N (0.7 mL, 5.0 mmol, 2 eq.) and THF (5 mL) were added to a reaction flask. The mixture was deoxygenated by sonication while bubbling with nitrogen for 5 min. Then the acetylene substrate (2.75 mmol, 1.1 eq.) was added and the reaction mixture was stirred at RT under N_2 atmosphere till complete consumption of the 2-iodoaniline (2-3 h). Then the reaction mixture was filtered through celite and the solvent was removed *in vacuo*. The crude products of **6b-e** were purified by flash column chromatography.

The TMS-protected crudes were deprotected via addition of K_2CO_3 (496 mg, 3.59 mmol, 1.4 eq.) and MeOH (10 mL). The reaction mixtures were stirred at RT for 60-90 min. Then the MeOH was removed *in vacuo* and water (5 mL) and Et_2O (10 mL) were added. The organic layer was separated and the aqueous layer was extracted with Et_2O (3 x 10 mL). The combined organic fractions were dried over Na_2SO_4 and the solvent was removed *in vacuo*. The crude products of **1b-e** were purified by flash column chromatography.

5-chloro-2-ethynylaniline (1b)

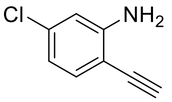

Substrate **1b** was synthesised following procedure **GP1** using 5-chloro-2-iodoaniline and (trimethylsilyl)acetylene as reagents. The crude was purified by flash column chromatography (pentane:EtOAc = 9:1 to 7:3) to yield the product (94 mg, 0.62 mmol, 25%) as an orange solid. ^{1}H NMR (400 MHz, $CDCl_3$) δ 7.23 (d, J = 8.2 Hz, 1H), 6.69 (d, J = 2.0 Hz, 1H), 6.64 (dd, J = 8.2, 2.0 Hz, 1H), 4.30 (s, 2H), 3.40 (s, 1H). The spectrum is in agreement with literature data.[36]

2-ethynyl-4-nitroaniline (1c)

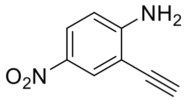

Substrate **1c** was synthesised following procedure **GP1** using 2-iodo-4-nitroaniline and (trimethylsilyl)acetylene as reagents. The crude was purified by flash column chromatography (pentane:EtOAc = 9:1 to 7:3) to yield the product (125 mg, 0.77 mmol, 31%) as a yellow solid. ^{1}H NMR (400 MHz, $CDCl_3$) δ 8.26 (d, J = 2.6 Hz, 1H), 8.05 (dd, J = 9.0, 2.6 Hz, 1H), 6.68 (d, J = 9.0 Hz, 1H), 4.96 (s, 2H), 3.46 (s, 1H). The spectrum is in agreement with literature data.[37]

2-ethynyl-4-methoxyaniline (1d)

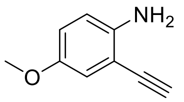

Substrate **1d** was synthesised following procedure **GP1** using changed amounts of the reagents 2-iodo-4-methoxyaniline (0.23 mL, 1.66 mmol) and (trimethylsilyl)acetylene (0.26 mL, 1.83 mmol). The amount (in mmol) of the other reagents is unchanged. The crude was purified by flash column chromatography (pentane:EtOAc = 9:1 to 7:3) to yield the product (80 mg, 0.54 mmol, 33%) as a yellow oil. ^{1}H NMR (400 MHz, $CDCl_3$) δ 6.87 (d, J = 2.9 Hz, 1H), 6.78 (dd, J = 8.8, 2.9 Hz, 1H), 6.65 (d, J = 8.8 Hz, 1H), 3.97 (s, 2H), 3.73 (s, 3H), 3.38 (s, 1H). The spectrum is in agreement with literature data.[37]

4-chloro-2-ethynylaniline (1e)

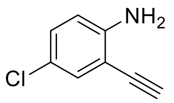

Substrate **1e** was synthesised following procedure **GP1** using 4-chloro-2-iodoaniline and (trimethylsilyl)acetylene as reagents. The crude was purified by flash column chromatography (pentane:EtOAc = 9:1 to 7:3) to yield the product (103 mg, 0.68 mmol, 27%) as an orange solid. ^{1}H NMR (400 MHz, $CDCl_3$) δ 7.28 (d, J = 2.5 Hz, 1H), 7.09 (dd, J = 8.7, 2.5 Hz, 1H), 6.62 (d, J = 8.7 Hz, 1H), 4.24 (s, 2H), 3.41 (s, 1H). The spectrum is in agreement with literature data.[37]

2-(phenylethynyl)aniline (6a)[38]

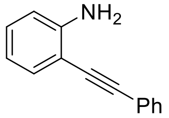

$Pd(PPh_3)_4$ (63 mg, 0.055 mmol, 0.02 eq.) and CuI (2.6 mg, 0.014 mmol, 0.005 eq.) were added to a 50 mL 2-necked flask equipped with a stirring bar. The flask was evacuated and refilled with N_2 (x3). A solution of 2-iodoaniline (2.73 g, 12.47 mmol, 4.6 eq.) and phenylacetylene (0.3 mL, 2.73 mmol, 1 eq.) in dry Et_3N (7 mL) was added to the mixture. The reaction was stirred for 4 h at RT under N_2 atmosphere. The mixture was diluted in toluene and concentrated *in vacuo*. After filtration over a celite plug. White precipitate was formed in the filtrate, which was then filtered off and the solvent was evaporated *in vacuo*. The crude was purified by flash column chromatography (pet. ether:EtOAc 20:1) to yield the product (356 mg, 1.84 mmol, 68%) as an off-white solid. ^{1}H NMR (400 MHz, DMSO-d_6) δ 7.60 (dd, J = 7.7, 1.8 Hz, 2H), 7.46 – 7.34 (m, 3H), 7.24 (dd, J = 7.7, 1.6 Hz, 1H), 7.08 (td, J = 7.7, 7.2, 1.6 Hz, 1H), 6.74 (d, J = 8.2 Hz, 1H), 6.54 (t, J = 7.4 Hz, 1H), 5.49 (s, 2H). ^{13}C NMR (101 MHz, DMSO-d_6) δ 149.61, 131.76, 131.20, 129.80, 128.53, 128.18, 123.00, 115.79, 113.94, 105.43, 93.82, 86.94. HRMS (ESI +) *m/z*: calculated mass for $C_{14}H_{12}N$ [M+H]+ 194.09697, found 194.09621.

2-(p-tolylethynyl)aniline (6b)

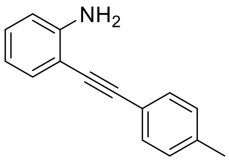

Substrate **6b** was synthesised following procedure **GP1** using 2-iodoaniline and 4-ethynyltoluene as reagents. The crude was purified by flash column chromatography (pentane:EtOAc = 19:1 to 9:1) to yield the product (395 mg, 1.91 mmol, 76%) as an orange solid. ^{1}H NMR (400 MHz, $CDCl_3$) δ 7.42 (d, J = 8.1 Hz, 2H), 7.39 – 7.33 (m, 1H), 7.20 – 7.10 (m, 3H), 6.72 (td, J = 7.8, 0.9 Hz, 2H), 4.27 (s, 2H), 2.37 (s, 3H). The spectrum is in agreement with literature data.[39]

2-((4-chlorophenyl)ethynyl)aniline (6c)

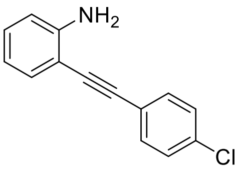

Substrate **6c** was synthesised following procedure **GP1** using 2-iodoaniline and 1-chloro-4-ethynylbenzene as reagents. The crude was purified by flash column chromatography (pentane:EtOAc = 19:1 to 9:1) to yield the product (462 mg, 2.03 mmol, 81%) as an orange solid. ^{1}H NMR (400 MHz, $CDCl_3$) δ 7.48 – 7.42 (m, 2H), 7.39 – 7.30 (m, 3H), 7.20 – 7.13 (m, 1H), 6.77 – 6.69 (m, 2H), 4.26 (s, 2H). The spectrum is in agreement with literature data.[39]

2-((4-fluorophenyl)ethynyl)aniline (6d)

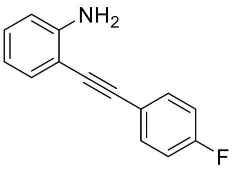

Substrate **7d** was synthesised following procedure **GP1** using 2-iodoaniline and 1-fluoro-4-ethynylbenzene as reagents. The crude was purified by flash column chromatography (pentane:EtOAc = 19:1 to 9:1) to yield the product (409 mg, 1.94 mmol, 77%) as an orange solid. ^{1}H NMR (400 MHz, $CDCl_3$) δ 7.56 – 7.45 (m, 2H), 7.39 – 7.32 (m, 1H), 7.20 – 7.12 (m, 1H), 7.09 – 6.99 (m, 2H), 6.72 (dd, J = 8.0, 6.8 Hz, 2H), 4.25 (s, 2H). The spectrum is in agreement with literature data.[39]

2-((4-methoxyphenyl)ethynyl)aniline (6e)

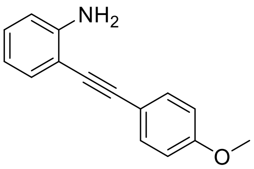

Substrate **7e** was synthesised following procedure **GP1** using 2-iodoaniline and 4-ethynylanisole as reagents. The crude was purified by flash column chromatography (pentane:EtOAc = 19:1 to 9:1) to yield the product (349 mg, 1.56 mmol, 63%) as an orange solid. ^{1}H NMR (400 MHz, $CDCl_3$) δ 7.50 – 7.44 (m, 2H), 7.38 – 7.32 (m, 1H), 7.17 – 7.09 (m, 1H), 6.92 – 6.84 (m, 2H), 6.74 – 6.67 (m, 2H), 4.26 (s, 2H), 3.83 (s, 3H). The spectrum is in agreement with literature data.[39]

1-(2-ethynylphenyl)-3-phenylurea (3)

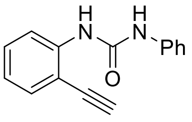

2-ethynylaniline (0.20 mL, 1.76 mmol, 1 eq.), phenyl isocyanate (0.19 mL, 1.76 mmol, 1 eq.) and chloroform (3.5 mL) were added to a dried Schlenk tube. The reaction was stirred for 5 h at 80 °C under N_2 atmosphere. The crude mixture was concentrated *in vacuo*. Then the crude solid was washed with cold chloroform (2 x 3 mL). The residue was dried under vacuum overnight to yield the product (336 mg, 1.42 mmol, 81%) as a white solid. ^{1}H NMR (400 MHz, DMSO-d_6) δ 9.57 (s, 1H), 8.19 (s, 1H), 8.13 (d, J = 8.5 Hz, 1H), 7.50 – 7.44 (m, 2H), 7.44 (dd, J = 7.7, 1.6 Hz, 1H), 7.35 (td, J = 8.7, 8.1, 1.6 Hz, 1H), 7.30 (t, J = 7.9 Hz, 2H), 6.99 (td, J = 7.5, 1.2 Hz, 2H), 4.66 (s, 1H). The spectrum is in agreement with literature data.[40]

4-methylene-3-phenyl-3,4-dihydroquinazolin-2(1H)-one (4)

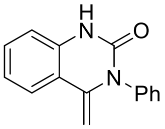

Compound **3** (96 mg, 0.41 mmol, 1 eq.), $NaAuCl_4$ (7.4 mg, 0.020 mol, 0.05 eq.) and $AgSbF_6$ (11.2 mg, 0.033 mmol, 0.08 eq.) were added to a Schlenk tube. The tube was evacuated and refilled with N_2 (x3) and 2 mL dry DMF was added. The mixture was heated to 60 °C and stirred for 5 h. The crude mixture was cooled to RT and diluted with DCM (4 mL). The solution was filtered over a celite plug and the solvent was evaporated *in vacuo*. The crude was purified by column chromatography (pet. ether:EtOAc 4:1) to yield the product (23 mg, 0.097 mmol, 24%) as a white solid. ^{1}H NMR (400 MHz, DMSO-d_6) δ 10.30 (s, 1H), 7.69 – 7.65 (m, 1H), 7.52 (t, J = 7.7 Hz, 2H), 7.43 (t, J = 7.4 Hz, 1H), 7.32 (t, J = 7.4 Hz, 1H), 7.26 (dd, J = 8.1, 1.4 Hz, 2H), 7.00 (td, J = 7.7, 7.2, 1.2 Hz, 1H), 6.94 (d, J = 8.0 Hz, 1H), 4.77 (d, J = 1.8 Hz, 1H), 3.39 (d, J = 1.9 Hz, 1H). The spectrum is in agreement with literature data.[40]

N-phenyl-1H-indole-1-carboxamide (5)

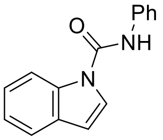

60% sodium hydride in mineral oil (260 mg, 6.50 mmol, 1.3 eq.) was dissolved in dry THF (25 mL). The solution was cooled to 4 °C and a solution of indole (586 mg, 5.00 mmol, 1 eq.) in dry THF (15 mL) was added dropwise. The solution was stirred for 30 min at 4 °C. Then a solution of phenyl isocyanate (0.54 mL, 5.00 mmol, 1 eq.) in dry THF (15 mL) was added dropwise. The mixture was allowed to warm to RT and stirred under N_2 atmosphere for 16 h. The reaction was quenched with water (50 mL). The solution was diluted with EtOAc (50 mL). The organic layer was separated and the aqueous layer was extracted with EtOAc (3 x 40 mL). The combined organic fractions were washed with brine (100 mL), dried over $MgSO_4$ and the solvent was removed *in vacuo*. The crude was purified by column chromatography (pentane:EtOAc 20:1) to yield the product (265 mg, 1.12 mmol, 22%) as a white solid. ^{1}H NMR (400 MHz, DMSO-d_6) δ 10.04 (s, 1H), 8.23 (d, J = 8.3 Hz, 1H), 8.04 (d, J = 3.7 Hz, 1H), 7.70 – 7.62 (m, 3H), 7.39 (dd, J = 8.5, 7.4 Hz, 2H), 7.30 (ddd, J = 8.4, 7.1, 1.3 Hz, 1H), 7.22 (td, J = 7.5, 1.1 Hz, 1H), 7.18 – 7.10 (m, 1H), 6.76 (d, J = 3.6 Hz, 1H). The spectrum is in agreement with literature data.[40]

(S)-2-((tert-butoxycarbonyl)amino)-3-(4-(tert-butylthio)phenyl)propanoic acid (8)

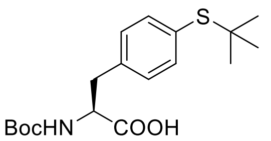

$Pd_2(dba)_3 \cdot CHCl_3$ (194 mg, 0.19 mmol, 0.015 eq.) and DPPF (474 mg, 0.85 mmol, 0.067 eq.) were added to a three-necked flask equipped with a stirring bar. The flask was evacuated and refilled with N_2 (x3) and 50 mL dry DMF was added. The mixture was stirred for 15-30 min at RT till a colour change from black to orange was observed. Then a solution of Et_3N (3.9 mL, 28.22 mmol, 2.2 eq.) and Boc-4-iodo-L-phenylalanine (4.99 g, 12.76 mmol, 1 eq.) in 70 mL dry DMF was added dropwise to the reaction mixture. The mixture was stirred for 10 min at RT. Then *t*-BuSH (1.6 mL, 14.02 mmol, 1.1 eq.) was added to the reaction mixture. The reaction was stirred for 3 h at 75 °C under N_2 atmosphere till all starting material was consumed. Then crude mixture was concentrated *in vacuo* and redissolved in EtOAc (100 mL) and 50 mM citric acid (100 mL). The organic layer was separated and the aqueous layer was extracted with EtOAc (2 x 100 mL). The combined organic fractions were washed with brine (3 x 50 mL), dried over Na_2SO_4 and the solvent was removed *in vacuo*. The crude was purified by flash column chromatography (pentane:EtOAc 3:2, 0.5 %v/v formic acid) to yield the product (3.65 g, 10.33 mmol, 81%) as a white solid. ^1H NMR (400 MHz, DMSO-d_6) δ 7.39 (d, J = 8.0 Hz, 2H), 7.26 (d, J = 7.9 Hz, 2H), 7.13 (d, J = 8.6 Hz, 1H), 4.14 (ddd, J = 10.5, 8.5, 4.4 Hz, 1H), 3.05 (dd, J = 13.8, 4.5 Hz, 1H), 2.83 (dd, J = 13.8, 10.6 Hz, 1H), 1.29 (s, 9H), 1.21 (s, 9H). ^{13}C NMR (101 MHz, DMSO-d_6) δ 173.44, 155.36, 139.08, 136.78, 129.70, 129.55, 77.99, 54.79, 45.43, 36.24, 30.60, 28.13. HRMS (ESI +) *m/z*: calculated mass for $C_{18}H_{27}N_1O_4S_1Na_1$ [M+Na]+ 376.15585, found 376.15530. The spectra are in agreement with literature data.[2]

(S)-2-amino-3-(4-mercaptophenyl)propanoic acid disulfide (9, *p*SHF)

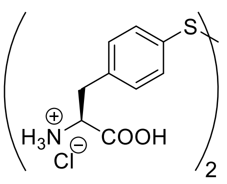

Compound **8** (3.65 g, 10.33 mmol) was dissolved in 37% HCl (125 mL). The mixture was stirred under reflux overnight and then allowed to cool to RT. The solution was washed with Et_2O (3 x 100 mL). The solution was concentrated *in vacuo* and then redissolved in 100 mL milliQ water. The resulting solution was lyophilised to yield the product (2.07 g, 4.45 mmol, 86%) as an off-white solid. ^1H NMR (400 MHz, DMSO-d_6) δ 8.55 (s, 3H), 7.46 (d, J = 8.0 Hz, 2H), 7.31 (d, J = 8.1 Hz, 2H), 4.09 (t, J = 6.3 Hz, 1H), 3.13 (d, J = 6.3 Hz, 2H). ^{13}C NMR (101 MHz, DMSO-d_6) δ 170.21, 134.87, 134.63, 130.77, 127.44, 53.07, 35.04. HRMS (ESI +) *m/z*: calculated mass for $C_{18}H_{21}N_2O_4S_2$ [M+H]+ 393.09372, found 393.09269. The spectra are in agreement with literature data.[2]

*^{1}H-NMR of compound **1b***

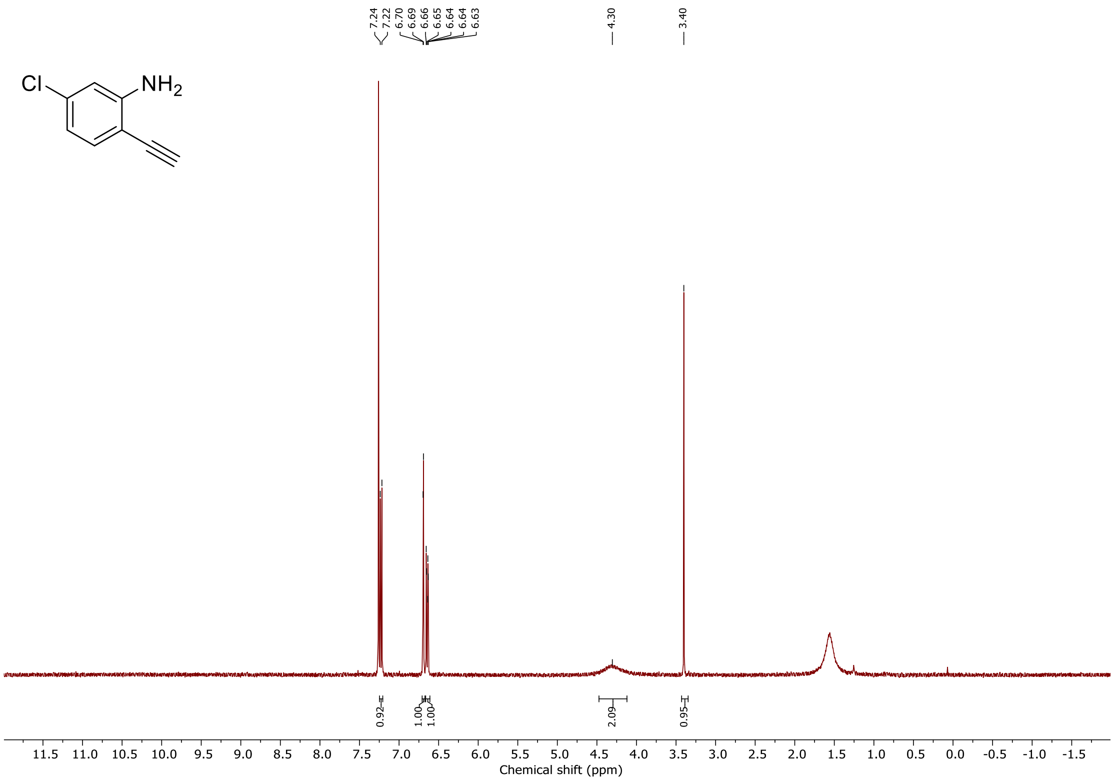

*^{1}H-NMR of compound **1c***

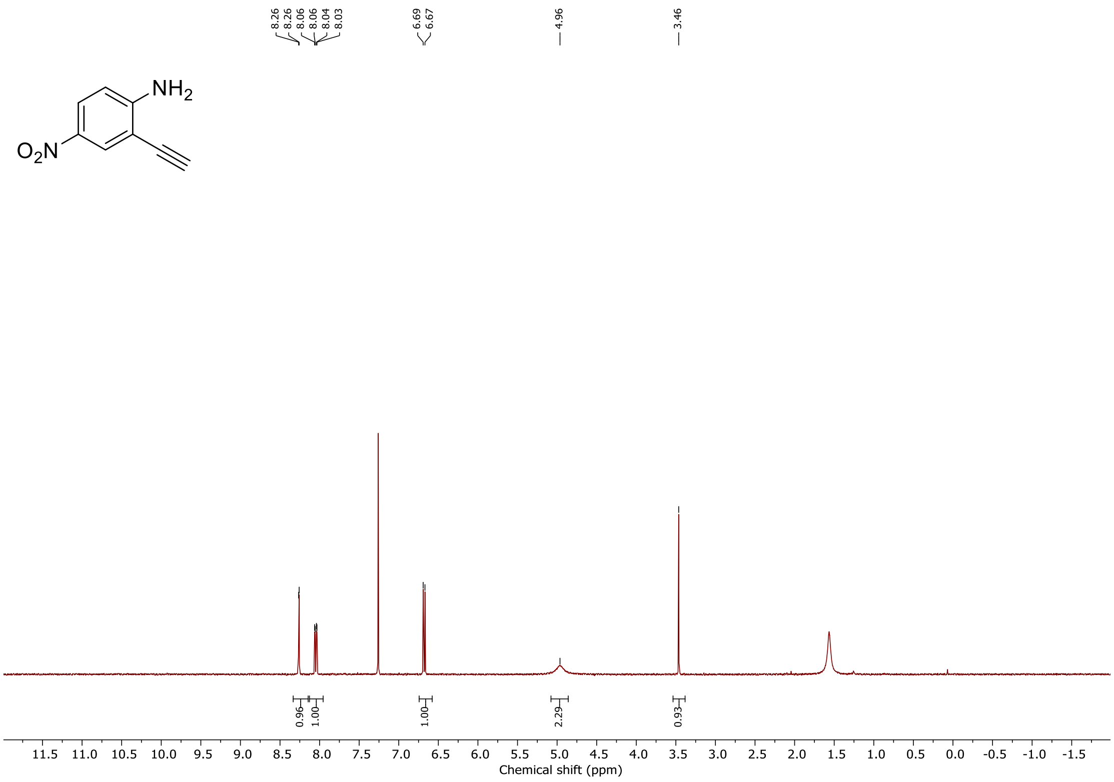

^{1}H-NMR of compound **1d**

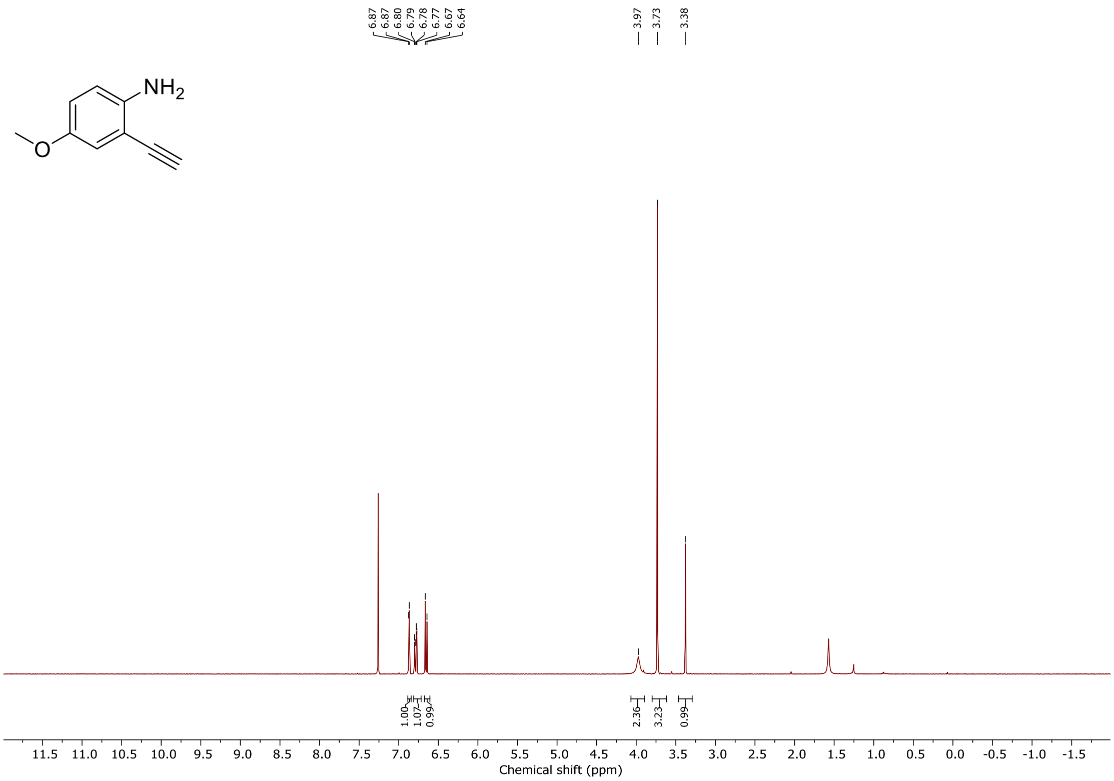

^{1}H-NMR of compound **1e**

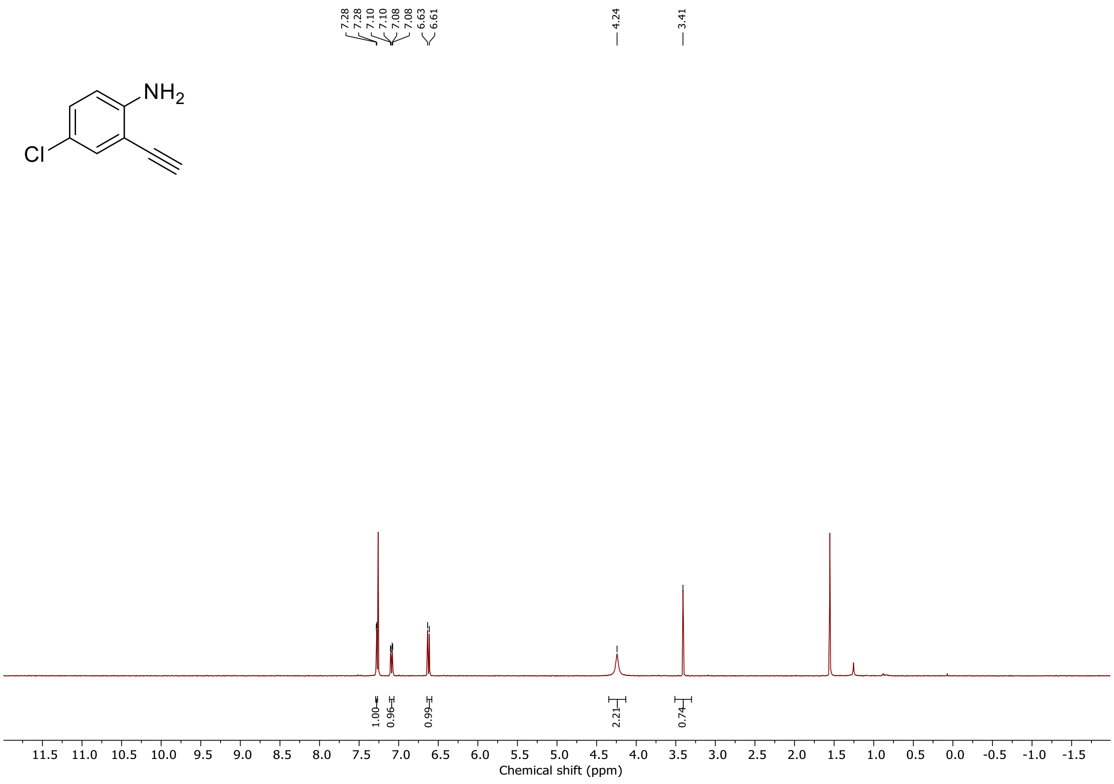

*^{1}H-NMR of compound **3***

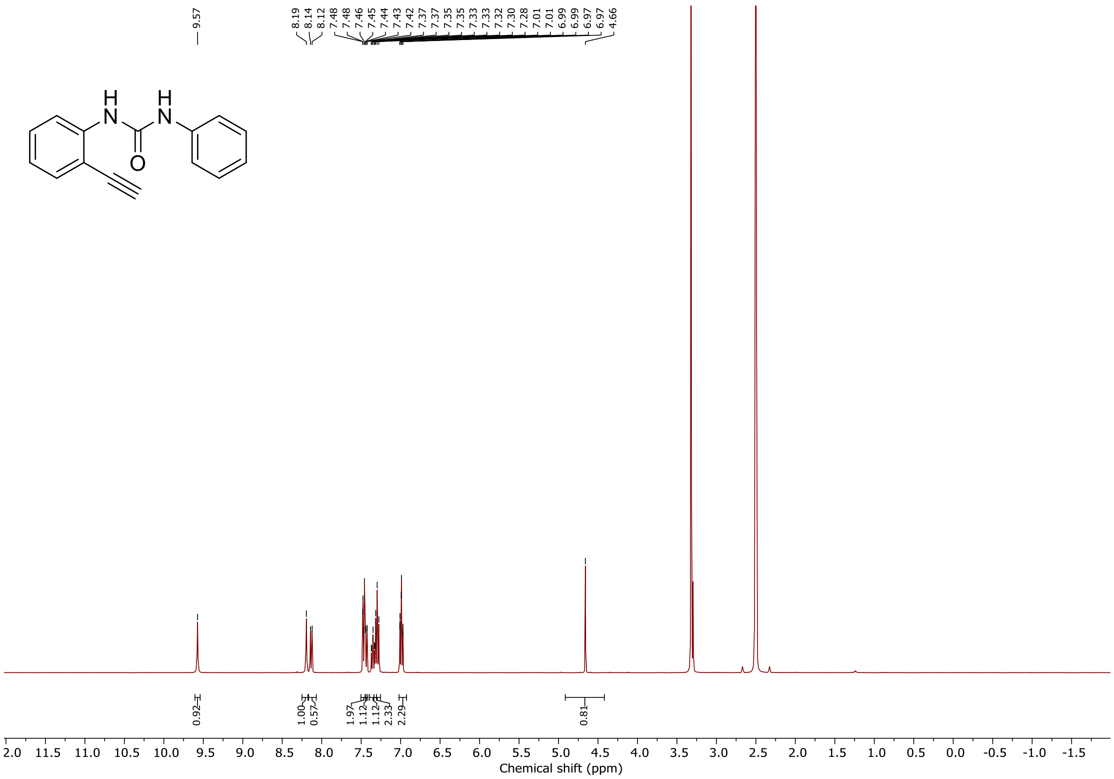

*^{1}H-NMR of compound **4***

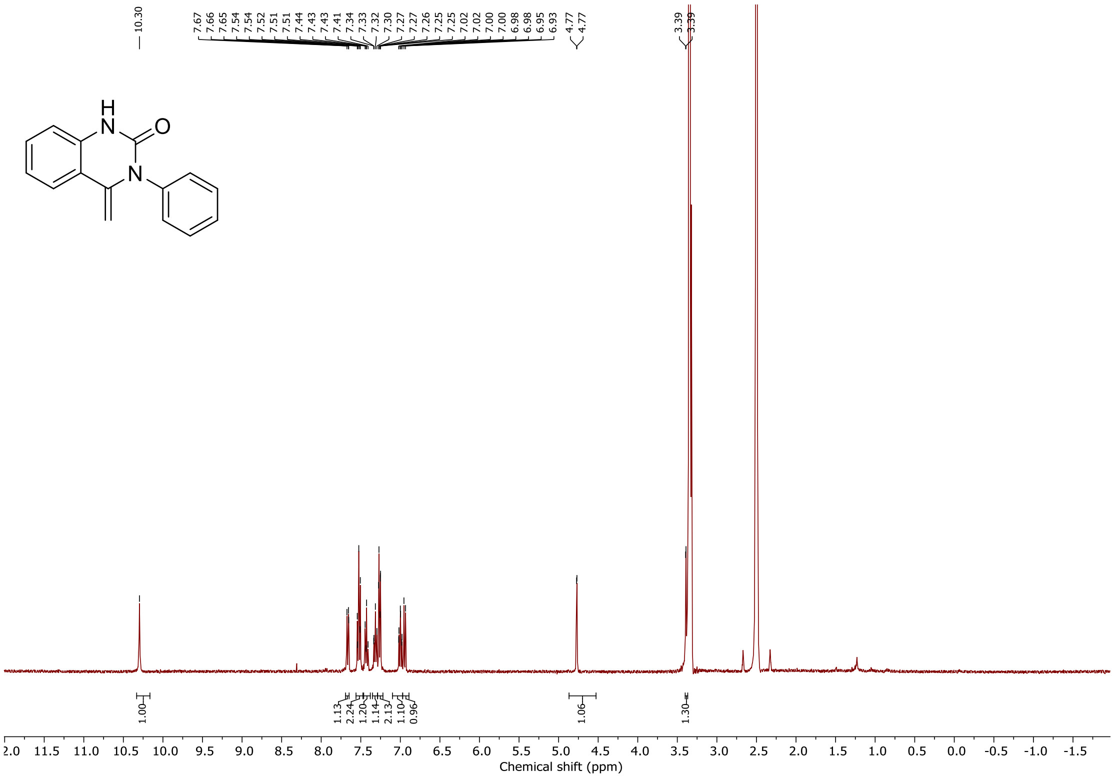

^{1}H-NMR of compound ***5***

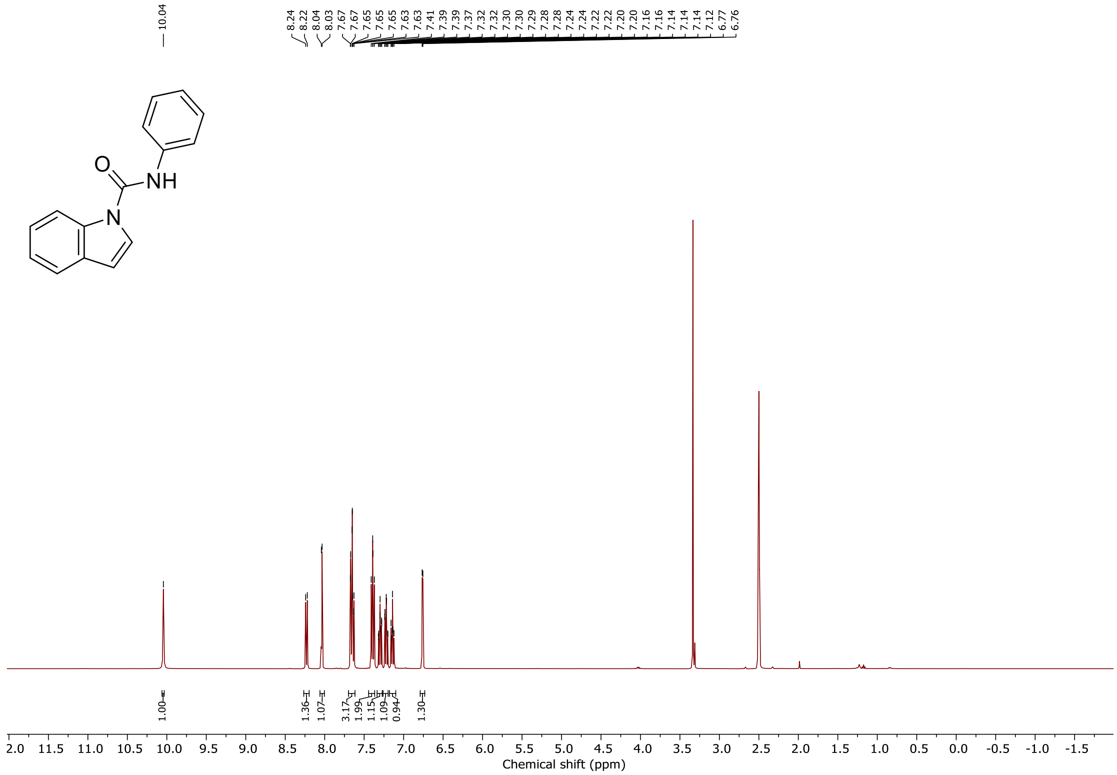

^{1}H-NMR of compound **6a**

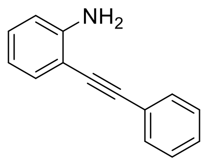

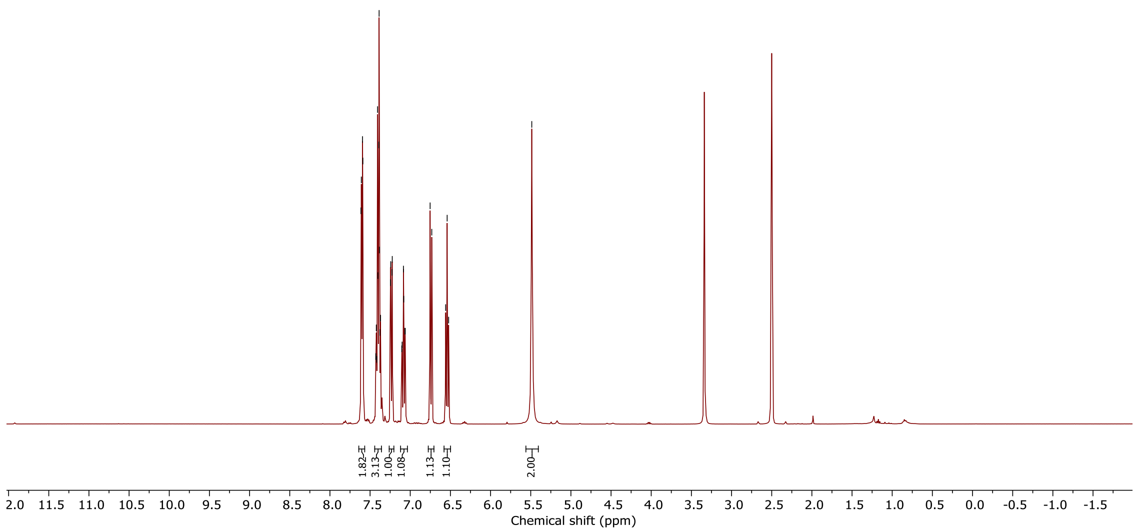

^{13}C-NMR of compound **6a**

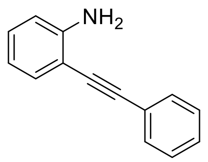

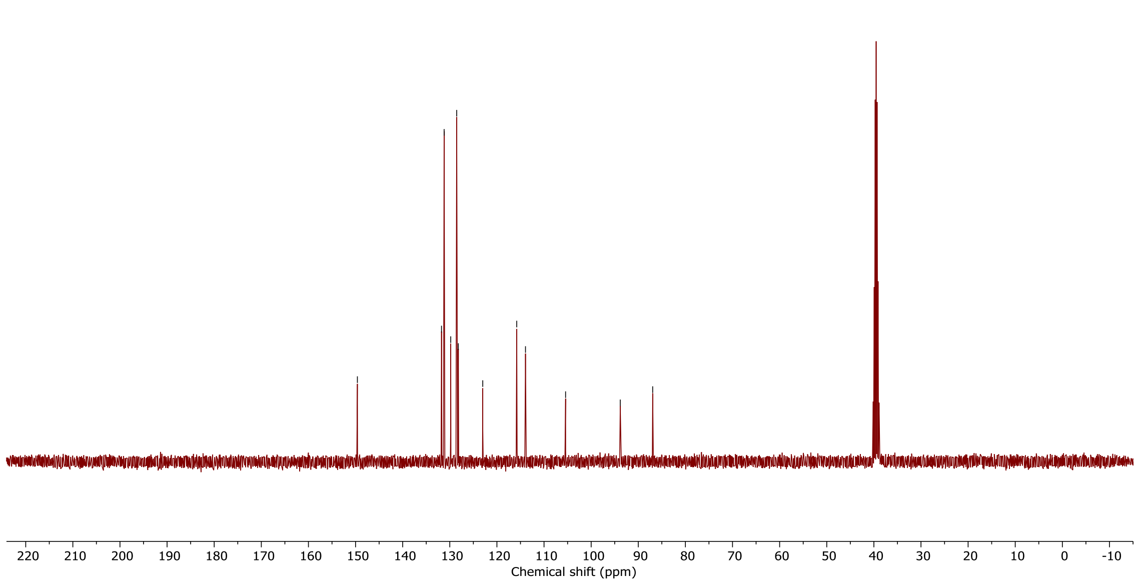

*^{1}H-NMR of compound **6b***

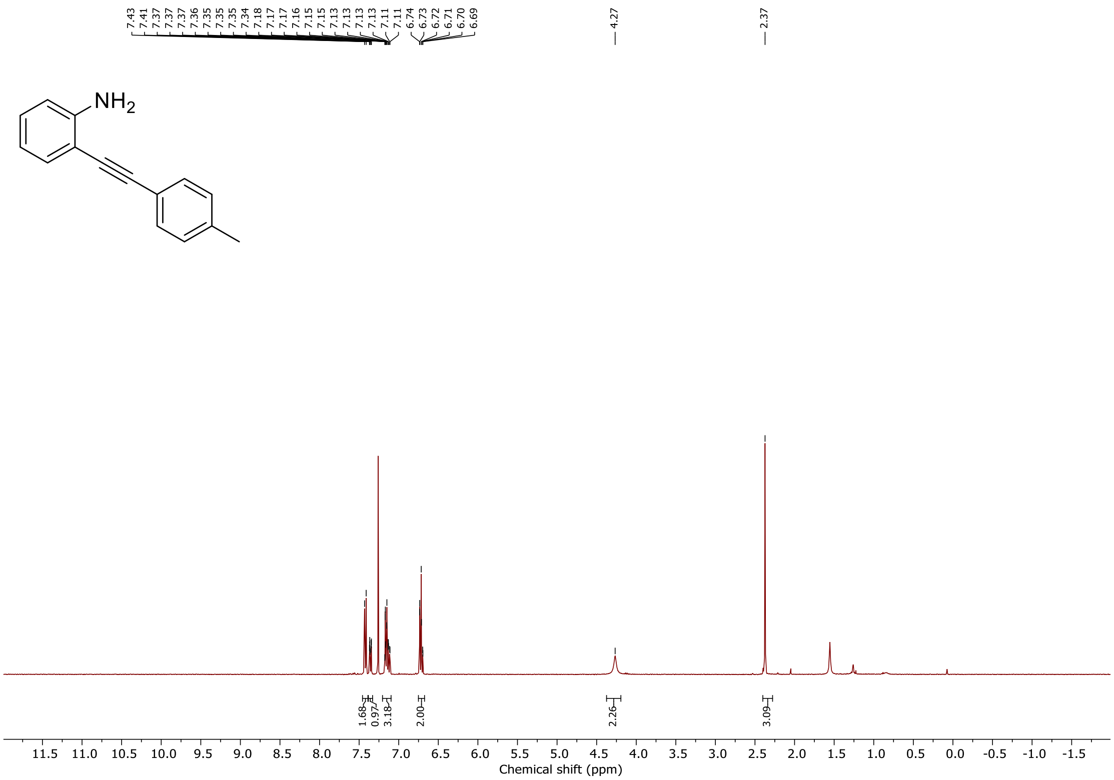

*^{1}H-NMR of compound **6c***

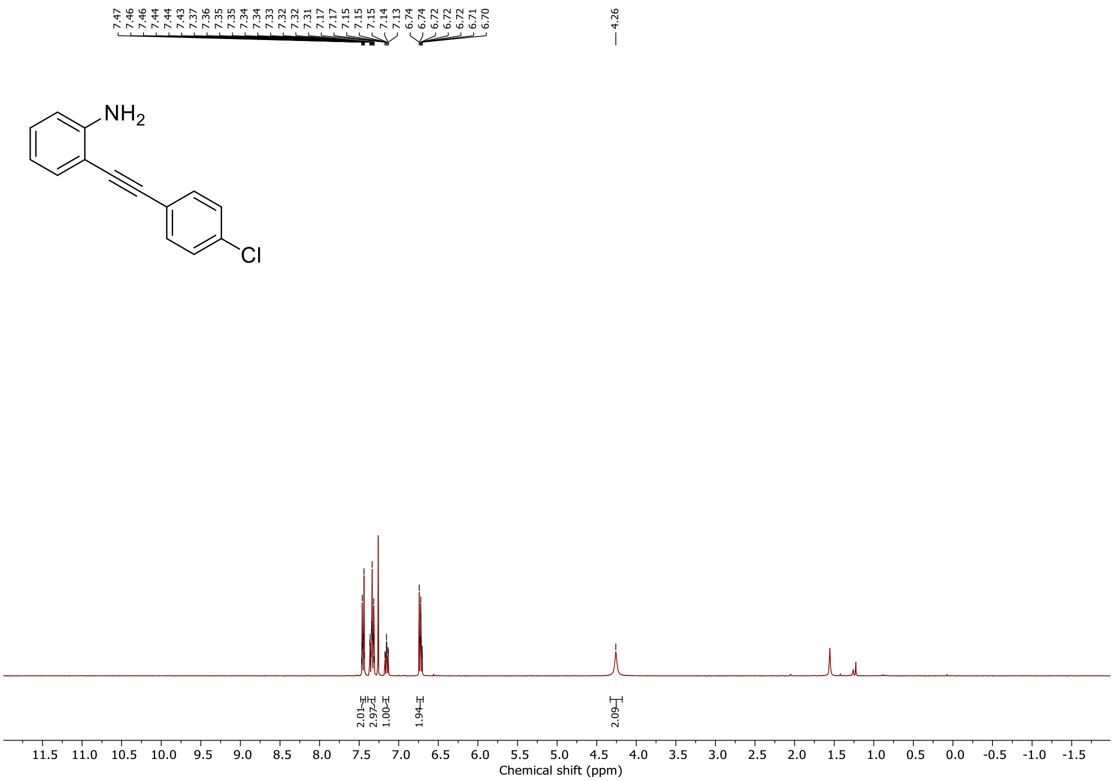

^{1}H-NMR of compound ***6d***

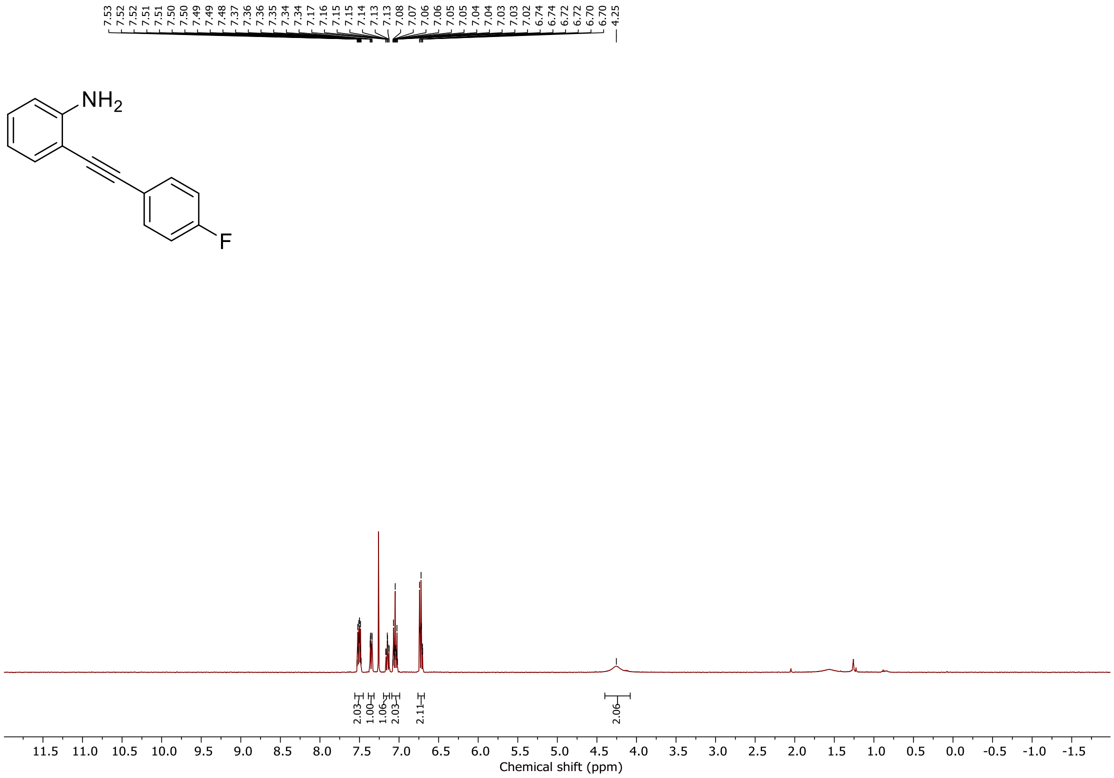

^{1}H-NMR of compound ***6e***

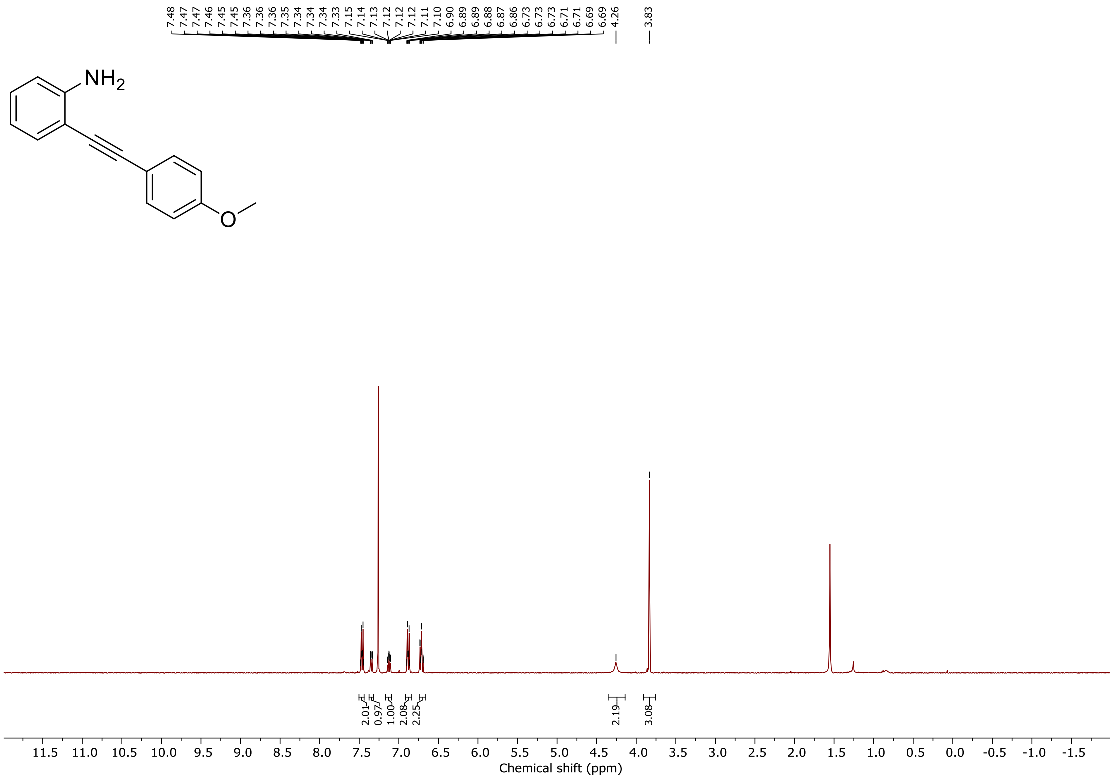

*^{1}H-NMR of compound **8***

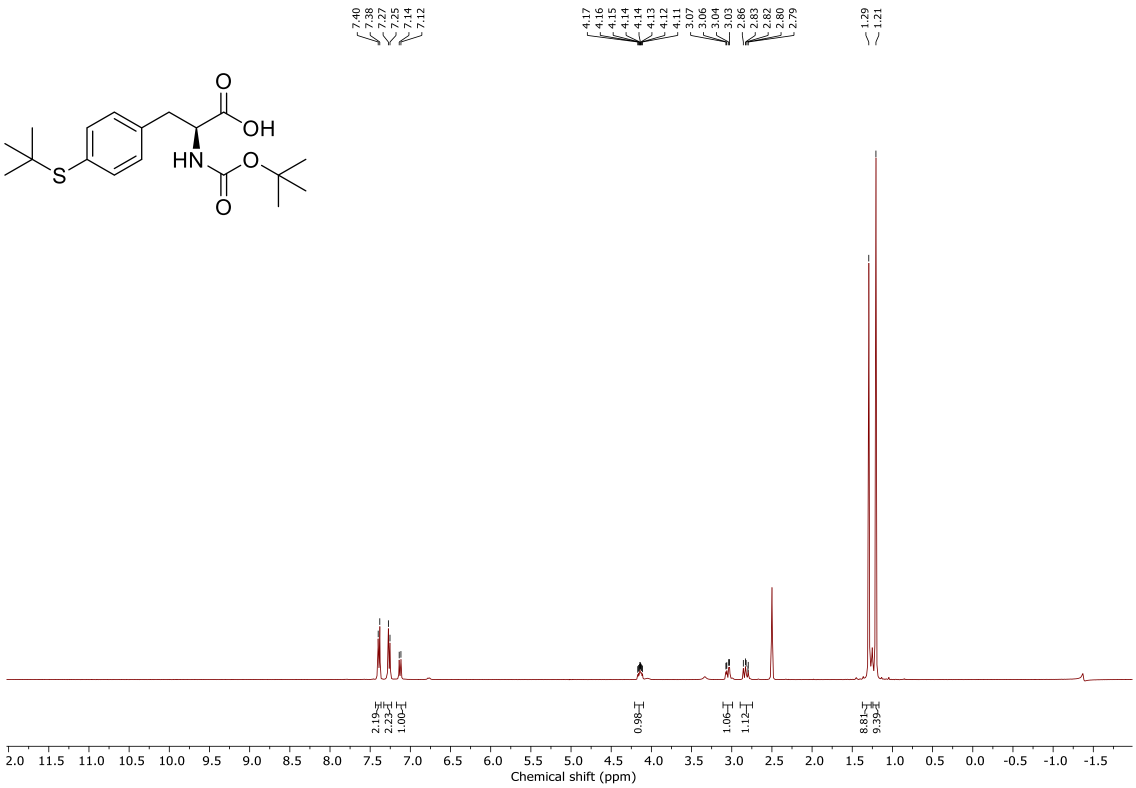

*^{13}C-NMR of compound **8***

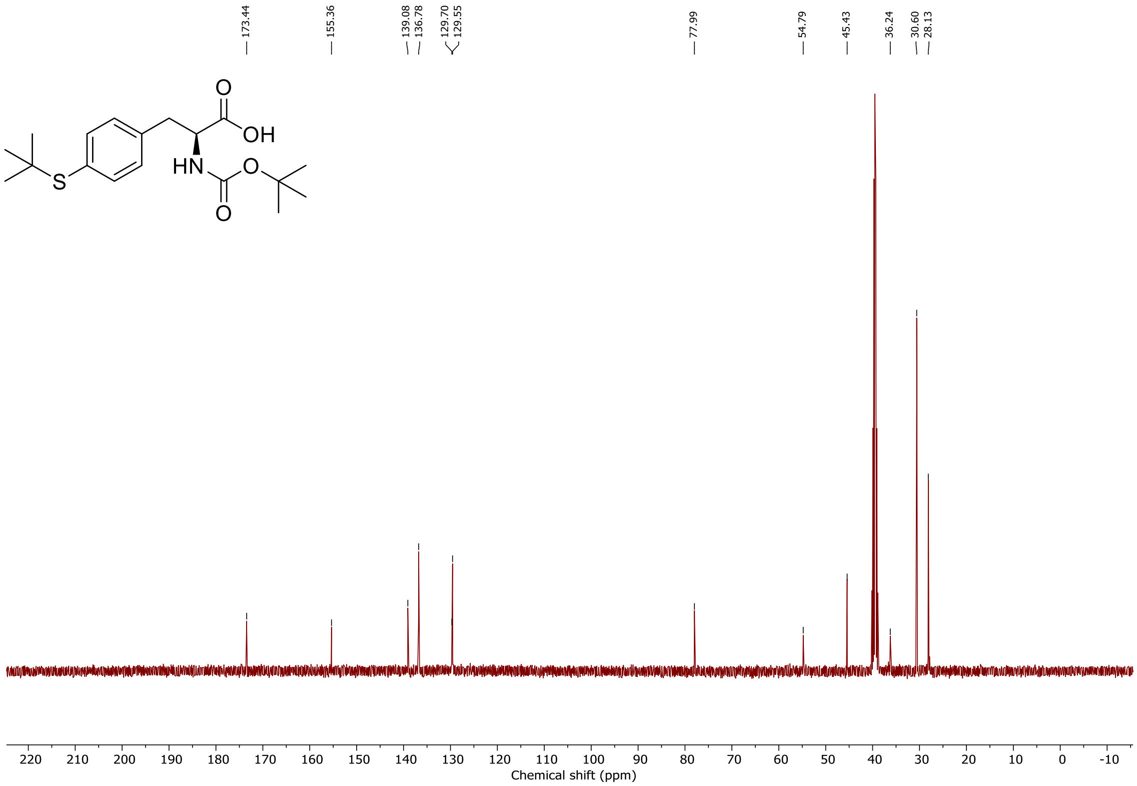

*^{1}H-NMR of compound **9***

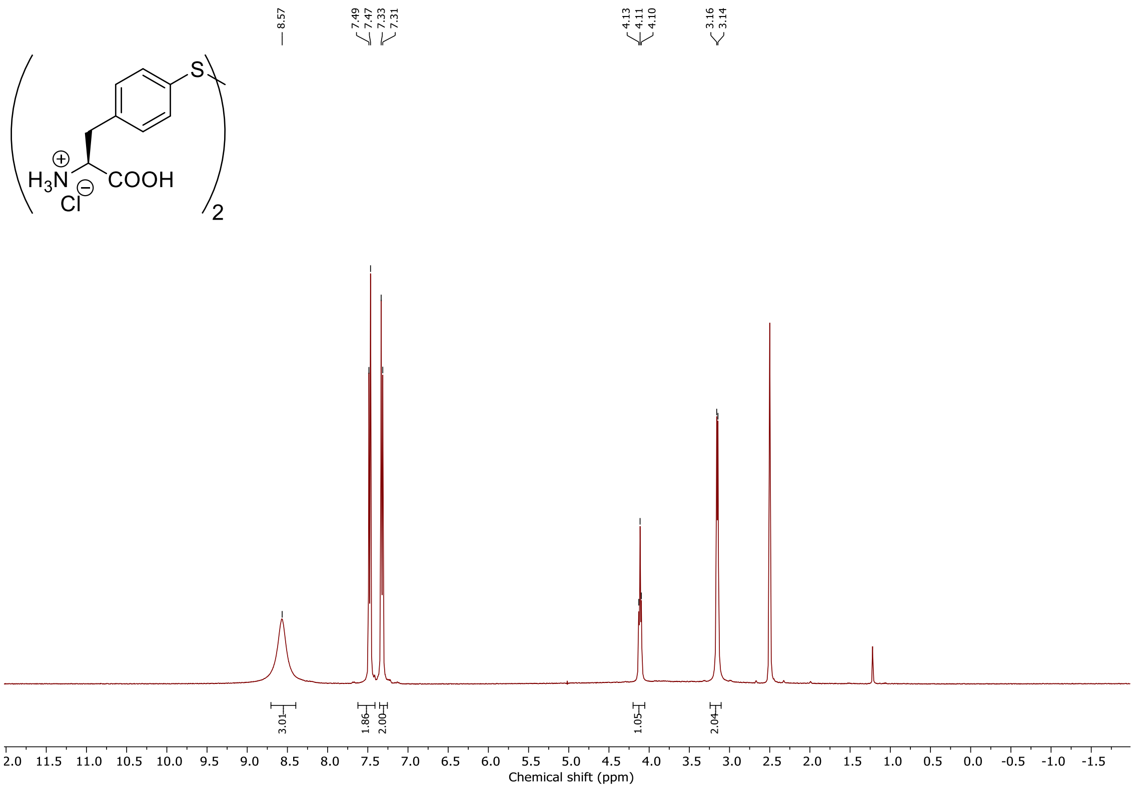

*^{13}C-NMR of compound **9***

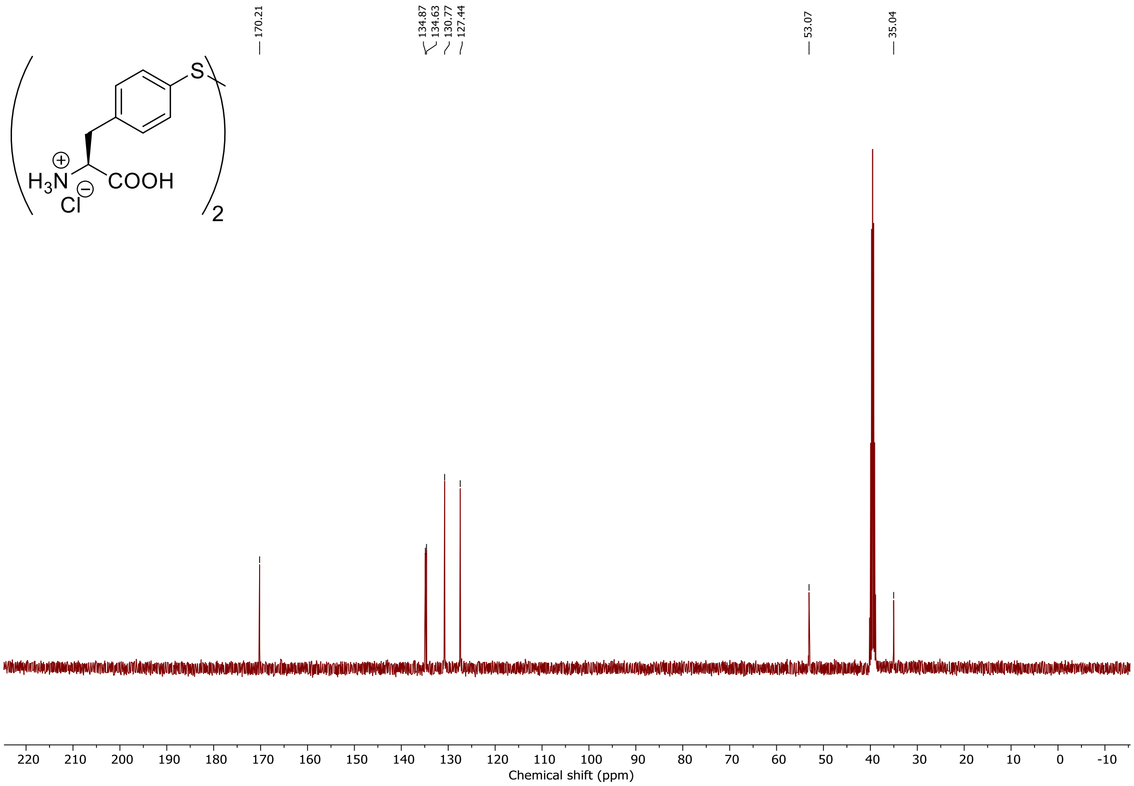

SI.30 GC-FID and SFC chromatograms and calibration curves

GC-FID calibrations

Compound 2a

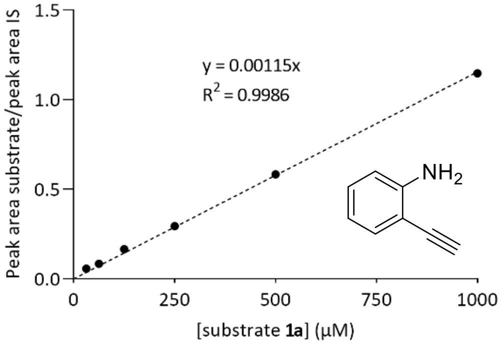

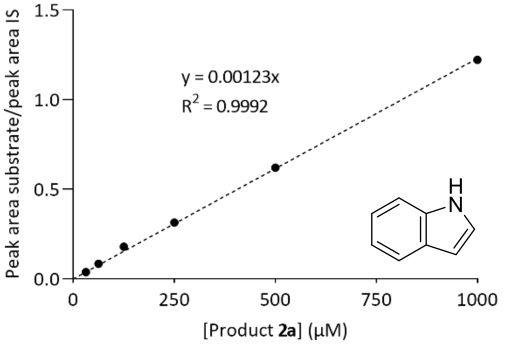

The samples were injected with a split ratio of 50 (1 µL injection volume) on an Agilent HP-5MS (25 m x 0.25 mm x 0.25 µm) column. The temperature program started at 60 °C and was ramped at 20 °C/min to 300 °C (12 min) using helium as carrier gas with a column flow of 0.88 mL/min. The injection temperature was set at 300 °C. The samples (150 µL) containing varying amounts of substrate were extracted with DCM (300 µL, 250 µM of mesitylene as internal standard). The organic layer was measured by GC-FID.

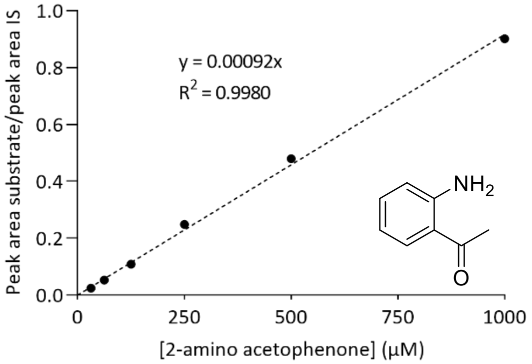

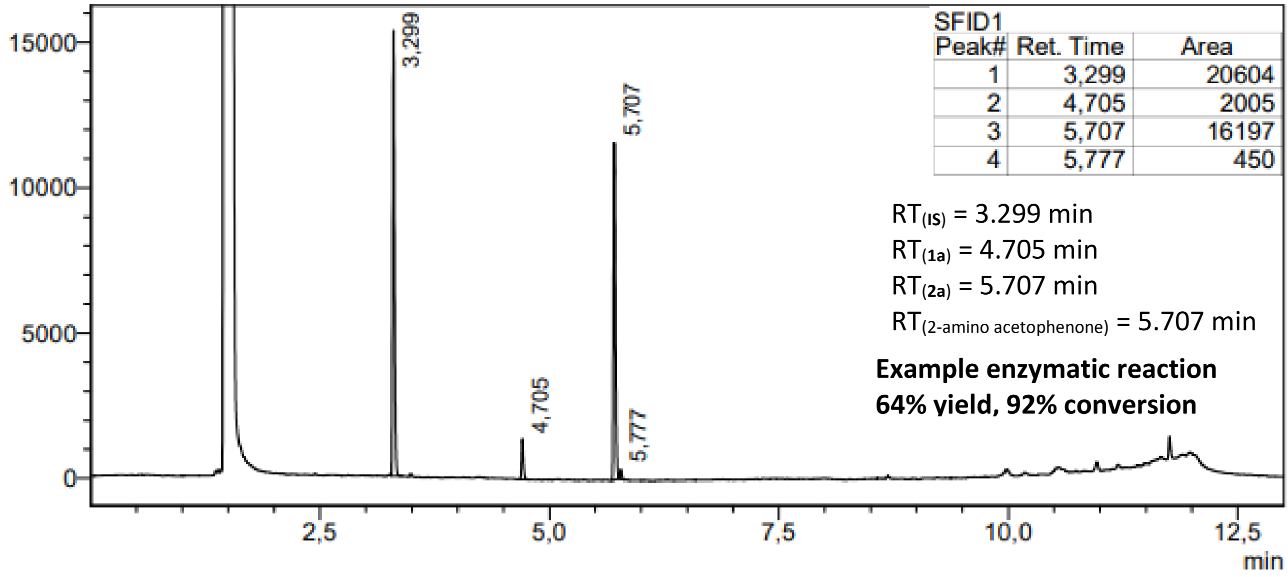

SFID1

Peak#	Ret. Time	Area
1	3,299	20604
2	4,705	2005
3	5,707	16197
4	5,777	450

$RT_{(IS)}$ = 3.299 min
$RT_{(1a)}$ = 4.705 min
$RT_{(2a)}$ = 5.707 min
$RT_{(2\text{-amino acetophenone})}$ = 5.707 min

Example enzymatic reaction
64% yield, 92% conversion

Compound 7a

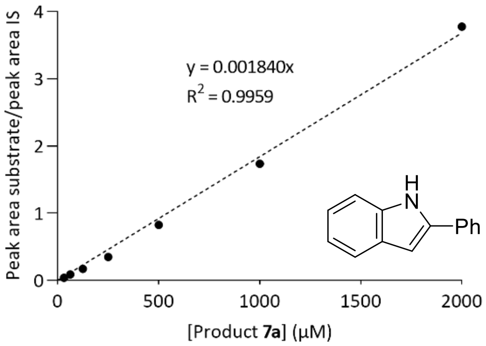

The samples were injected with a split ratio of 50 (1 µL injection volume) on an Agilent HP-5MS (25 m x 0.25 mm x 0.25 µm) column. The temperature program started at 60 °C and was ramped at 20 °C/min to 120 °C (3 min) followed by 30 °C/min to 300 °C (6 min) using helium as carrier gas with a column flow of 0.96 mL/min. The injection temperature was set at 300 °C. The samples (150 µL) containing varying amounts of substrate were extracted with DCM (300 µL, 250 µM of mesitylene as internal standard). The organic layer was separated and analysed by GC-FID.

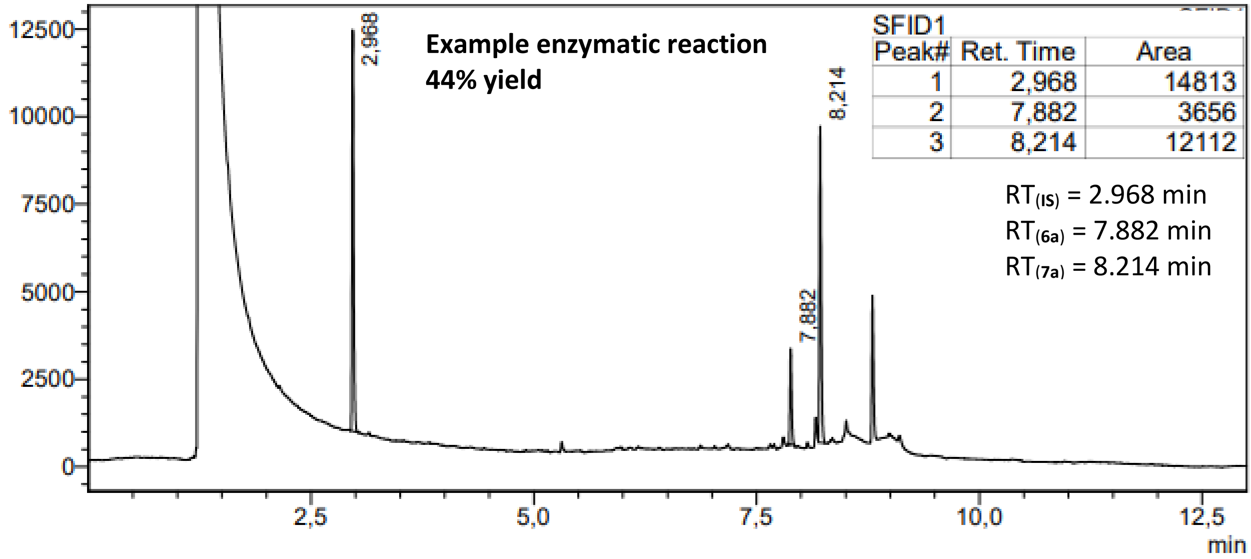

SFC calibrations

Compound 4 + 5

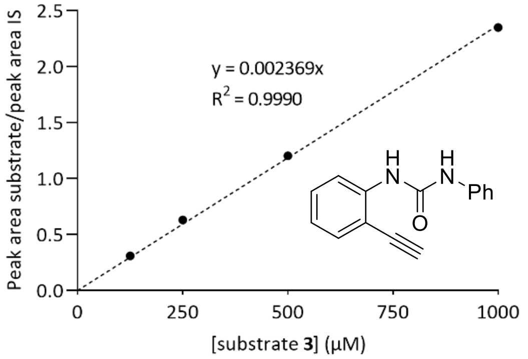

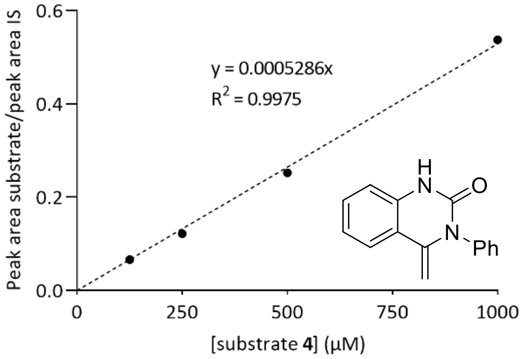

Retention time (min)	$scCO_2$ (%)	MeOH (%)
0.00	97	3
1.00	80	20
5.00	50	50
5.10	97	3
6.00	97	3

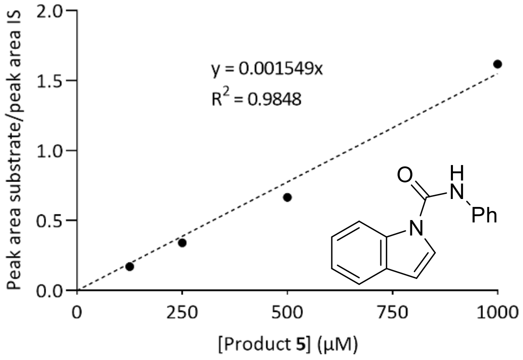

The samples were separated using a Trefoil CEL1 column with $scCO_2$ and MeOH as eluents. Flowrates are 1.8 mL/min and the injection volume is 3 µL. The samples (150 µL) containing varying amounts of substrate were extracted with DCM (300 µL, 250 µM of 2-phenylquinoline as internal standard). The sample was vortexed (1 min) and inverted (x3). The layers were separated by centrifugation (13000 g, 2 min). 290 µL of the organic layer was pipetted into a new microcentrifuge tube (1.5 mL) and the solvent was removed *in vacuo* (15 min, 30 °C). The residue was redissolved in SFC grade MeOH (80 µL). The samples were analysed by SFC.

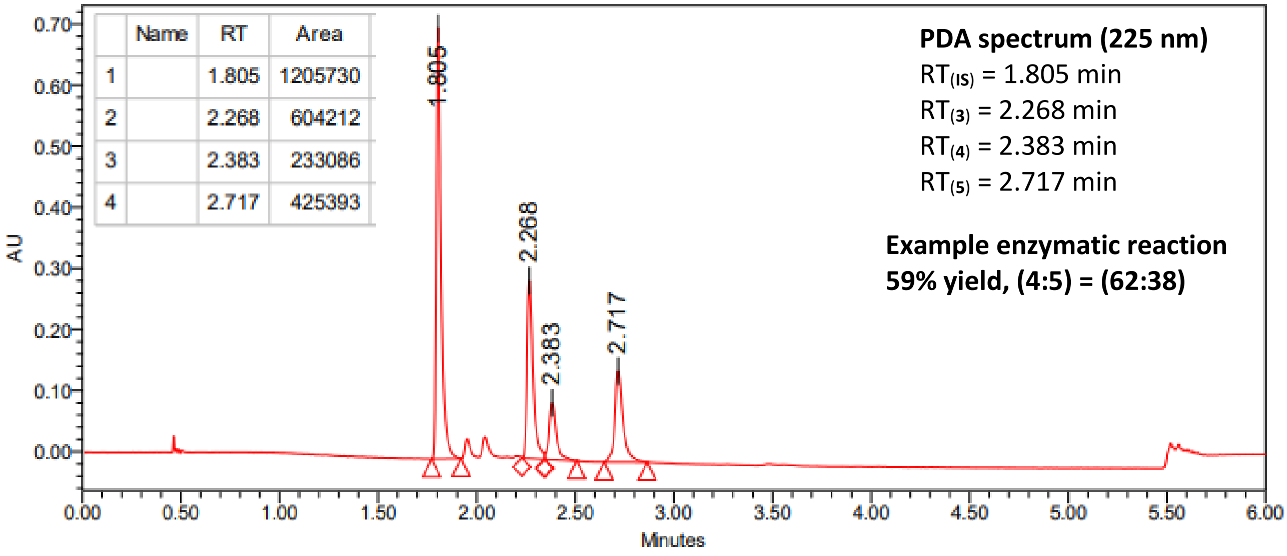

Kinetic- and time course measurements

Compound 7a

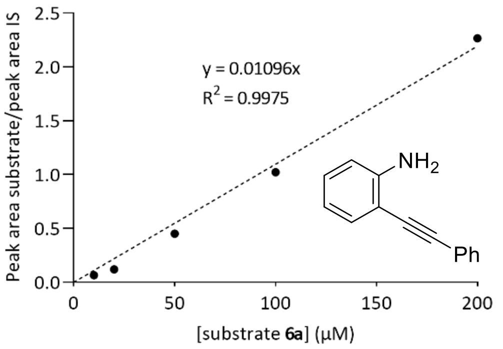

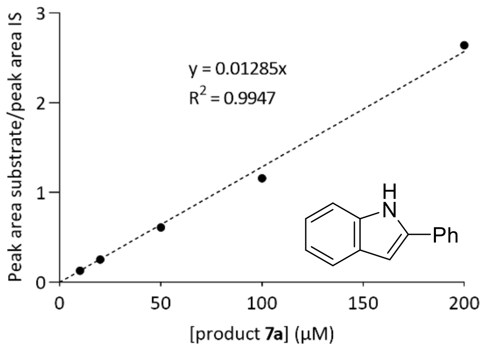

The samples were separated using a Trefoil CEL2 column with $scCO_2$ and MeOH as eluents. Flowrates are 1.8 mL/min and the injection volume is 6 µL. The samples (150 µL) containing varying amounts of substrate were extracted with DCM (300 µL, 50 µM of 2-phenylquinoline as internal standard). The sample was vortexed (1 min) and inverted (x3). The layers were separated by centrifugation (13000 g, 2 min). 290 µL of the organic layer was pipetted into a new microcentrifuge tube (1.5 mL) and the solvent was removed *in vacuo* (15 min, 30 °C). The residue was redissolved in SFC grade MeOH (80 µL). The samples were analysed by SFC.

Retention time (min)	$scCO_2$ (%)	MeOH (%)
0.00	97	3
3.00	75	25
4.50	50	50
5.00	97	3
6.00	97	3

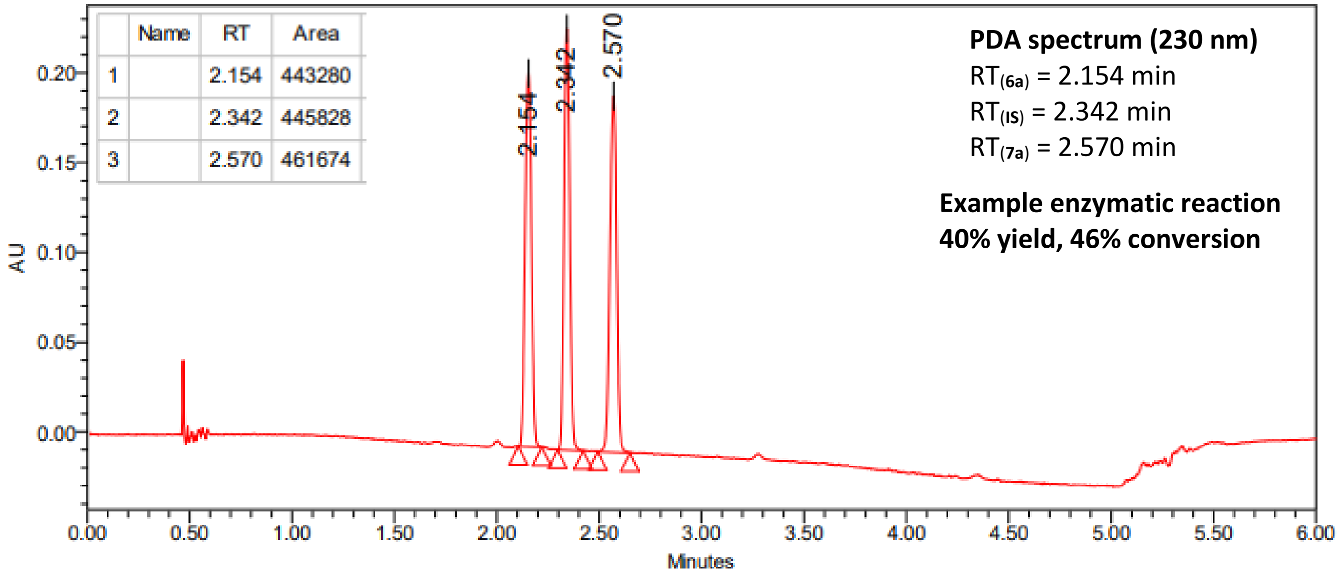

Substrate scope

The samples were separated using a Chiracel OJ-3 or Trefoil CEL1 column with $scCO_2$ and MeOH as eluents. Flowrates are 1.8 mL/min and the injection volume is 6 µL. The samples (150 µL) containing varying amounts of substrate were extracted with DCM (300 µL, 250 µM of 2-phenylquinoline as internal standard). The sample was vortexed (1 min) and inverted (x3). The layers were separated by centrifugation (13000 g, 2 min). 290 µL of the organic layer was pipetted into a new microcentrifuge tube (1.5 mL) and the solvent was removed *in vacuo* (15 min, 30 °C). The residue was redissolved in SFC grade MeOH (80 µL). The samples were analysed by SFC.

Compound 2a

Column = Chiracel OJ-3		
Retention time (min)	$scCO_2$ (%)	MeOH (%)
0.00	97	3
4.50	50	50
6.00	50	50
6.10	97	3
7.00	97	3

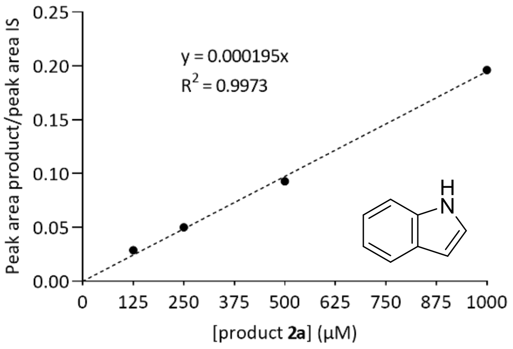

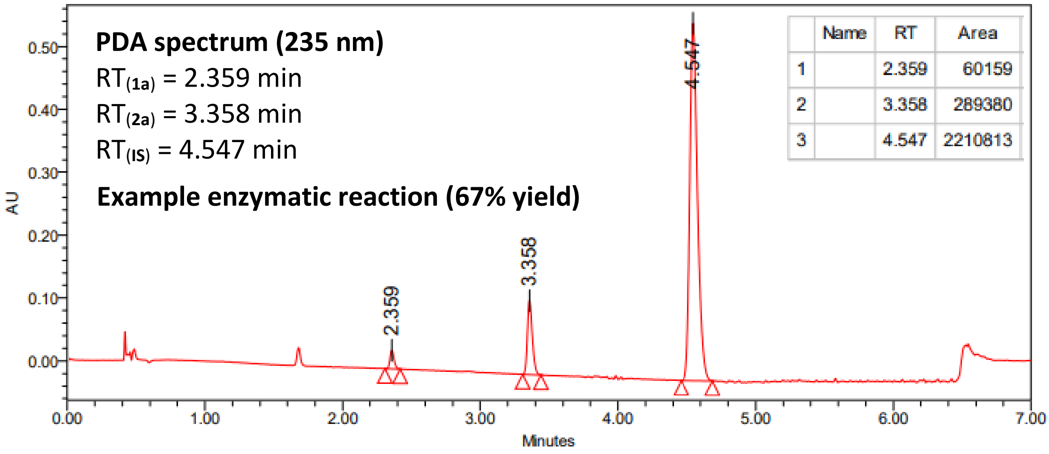

Compound 2b

Column = Chiracel OJ-3		
Retention time (min)	$scCO_2$ (%)	MeOH (%)
0.00	97	3
4.50	50	50
6.00	50	50
6.10	97	3
7.00	97	3

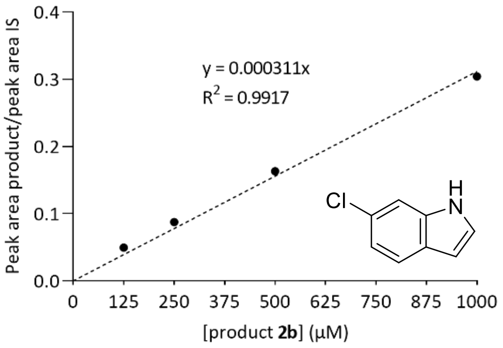

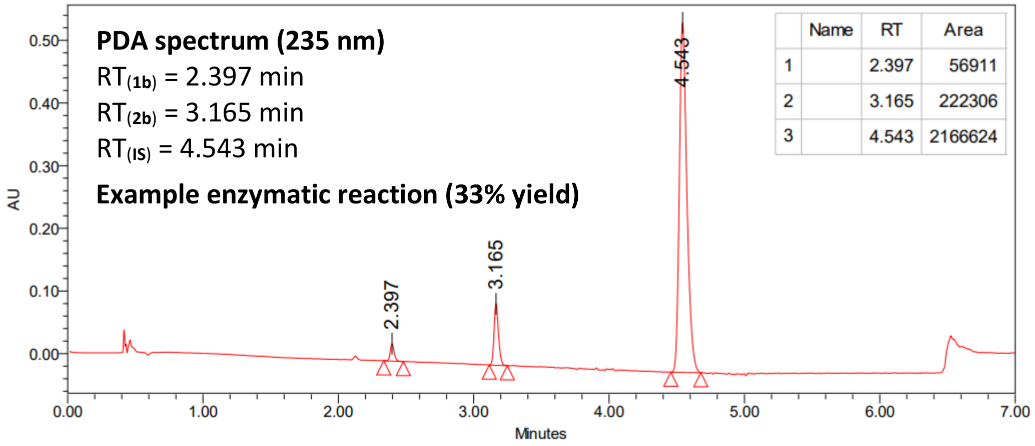

Compound 2c

Column = Trefoil CEL1		
Retention time (min)	$scCO_2$ (%)	MeOH (%)
0.00	97	3
1.00	90	10
8.00	70	30
8.10	97	3
9.00	97	3

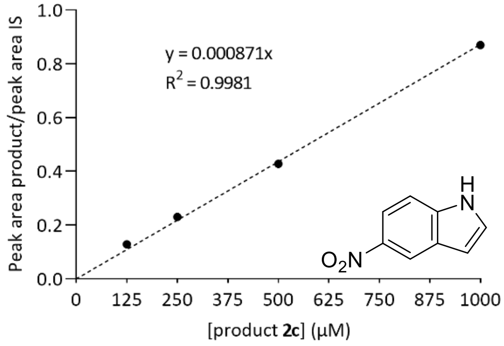

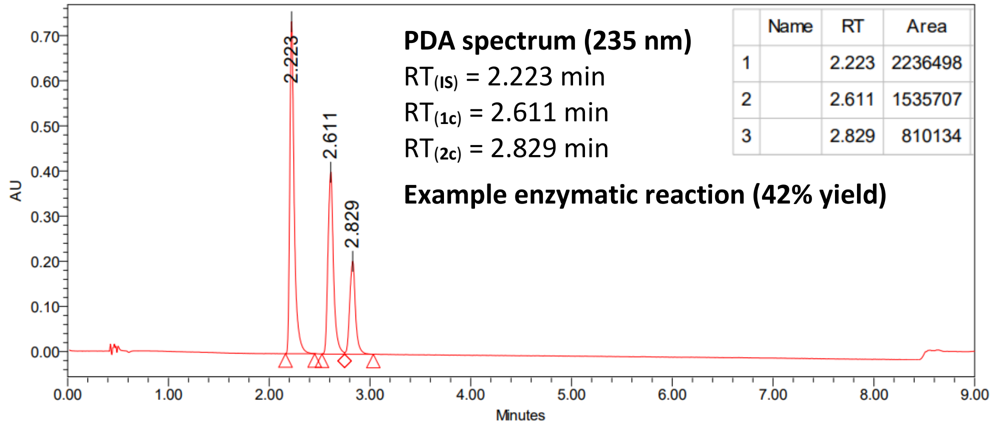

Compound 2d

Column = Chiracel OJ-3		
Retention time (min)	$scCO_2$ (%)	MeOH (%)
0.00	97	3
4.50	50	50
6.00	50	50
6.10	97	3
7.00	97	3

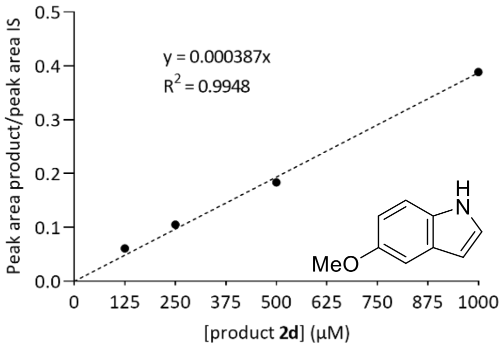

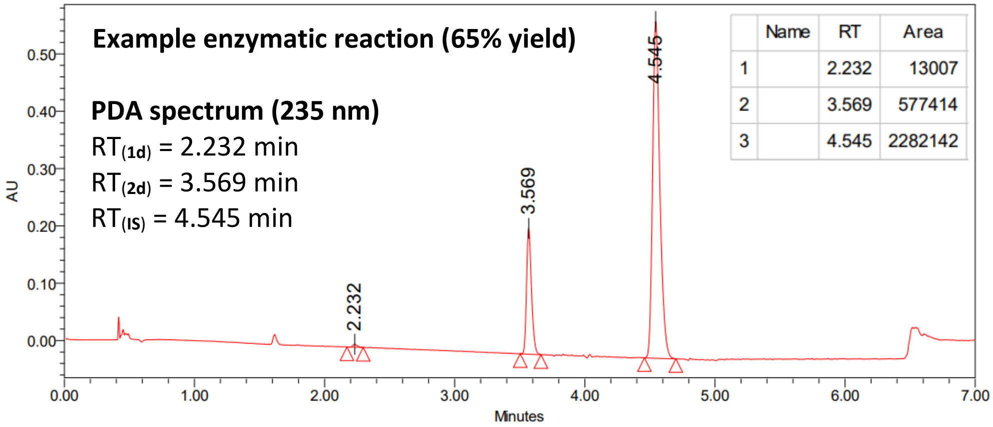

Compound 2e

Column = Chiracel OJ-3		
Retention time (min)	$scCO_2$ (%)	MeOH (%)
0.00	97	3
4.50	50	50
6.00	50	50
6.10	97	3
7.00	97	3

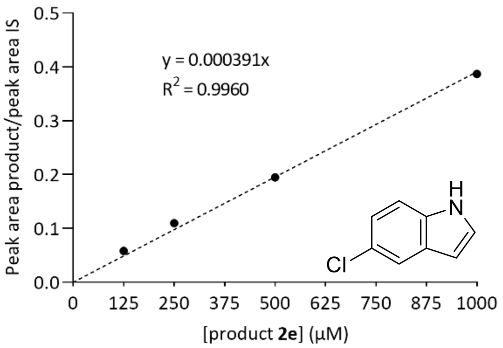

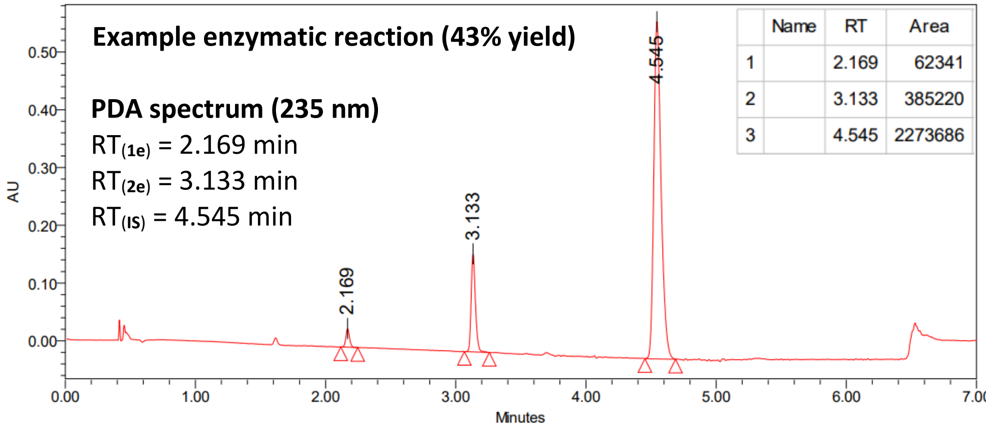

Compound 7a

Column = Trefoil CEL1		
Retention time (min)	$scCO_2$ (%)	MeOH (%)
0.00	97	3
4.50	50	50
6.00	50	50
6.10	97	3
7.00	97	3

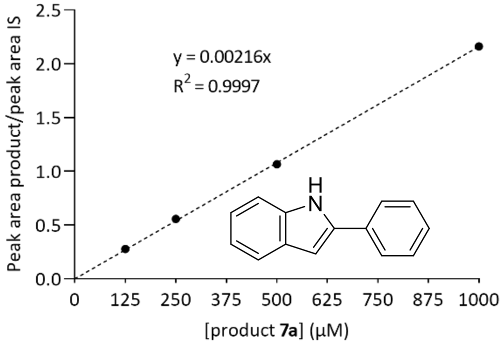

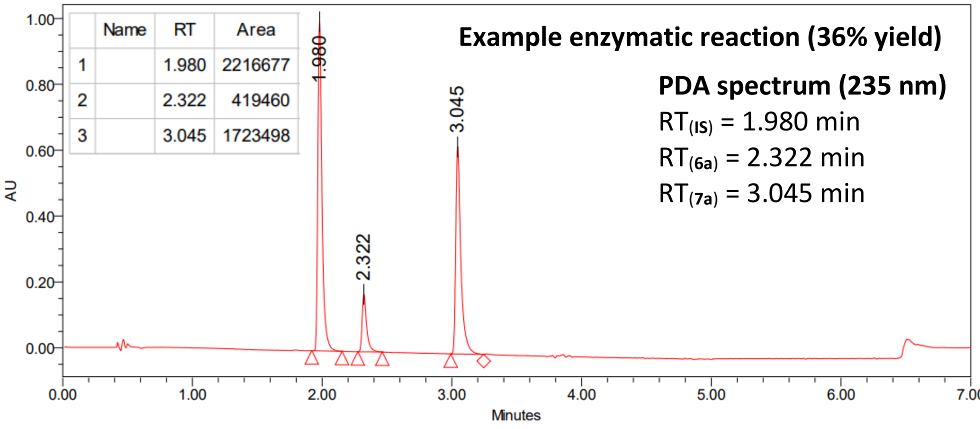

Compound 7b

Column = Trefoil CEL1		
Retention time (min)	$scCO_2$ (%)	MeOH (%)
0.00	97	3
4.50	50	50
6.00	50	50
6.10	97	3
7.00	97	3

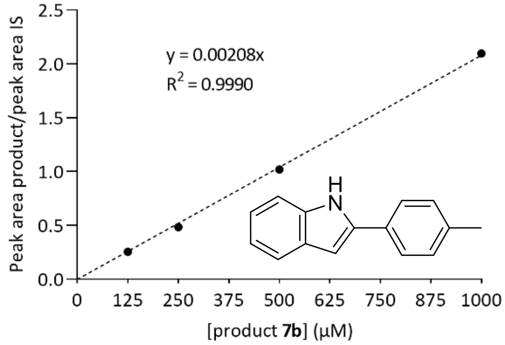

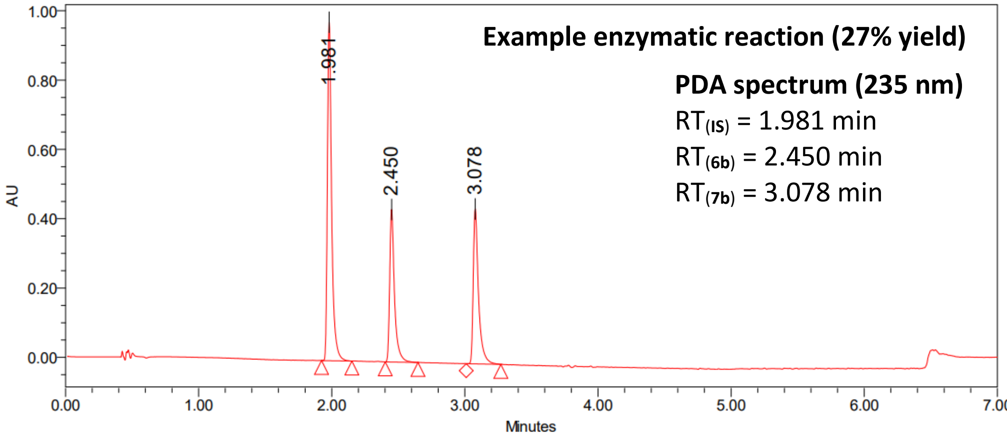

Compound 7c

Column = Trefoil CEL1		
Retention time (min)	$scCO_2$ (%)	MeOH (%)
0.00	97	3
4.50	50	50
6.00	50	50
6.10	97	3
7.00	97	3

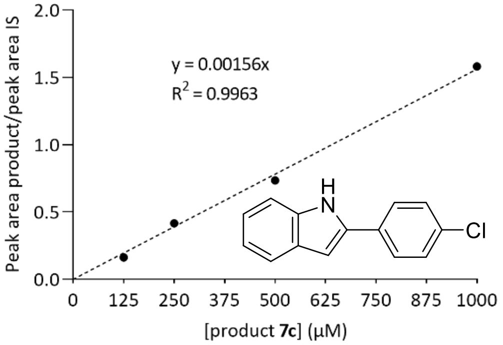

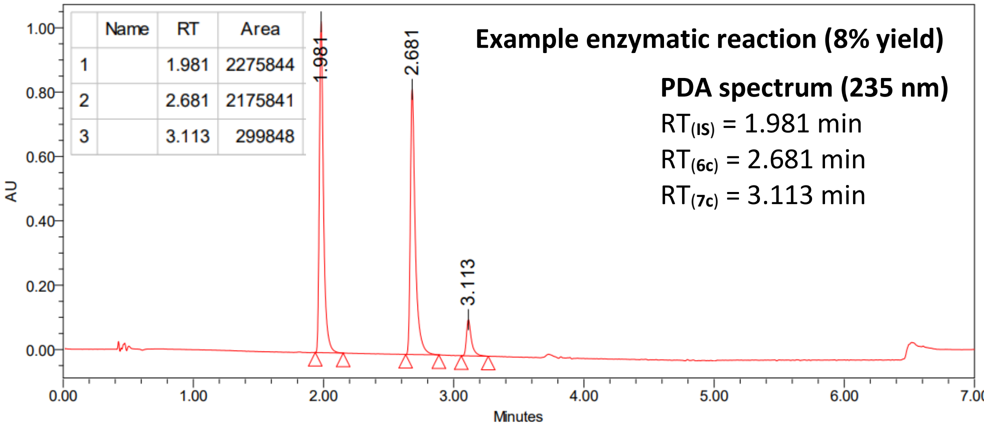

Compound 7d

Column = Trefoil CEL1		
Retention time (min)	$scCO_2$ (%)	MeOH (%)
0.00	97	3
4.50	50	50
6.00	50	50
6.10	97	3
7.00	97	3

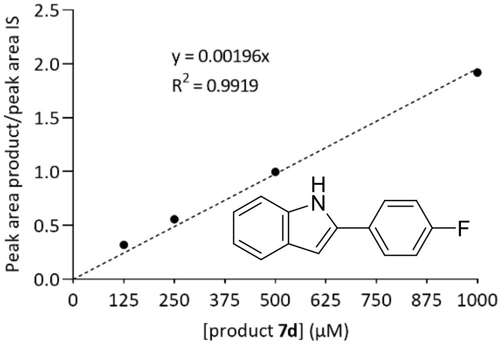

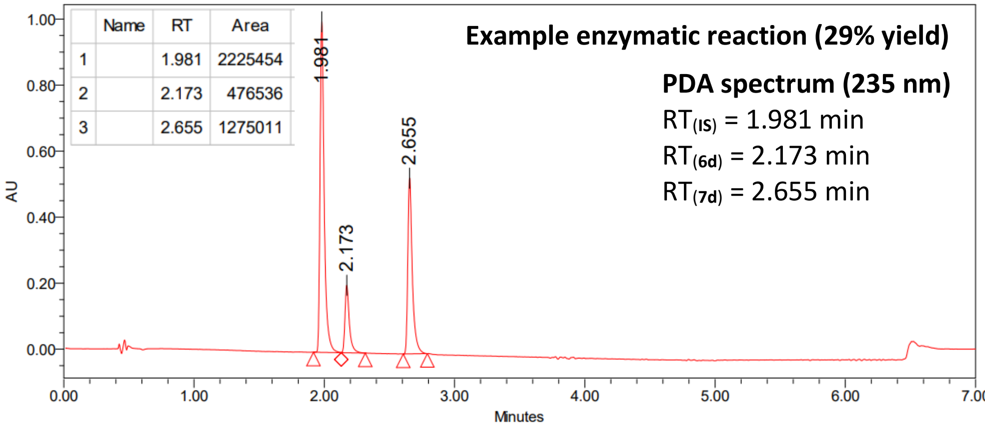

Compound 7e

Column = Trefoil CEL1		
Retention time (min)	$scCO_2$ (%)	MeOH (%)
0.00	97	3
4.50	50	50
6.00	50	50
6.10	97	3
7.00	97	3

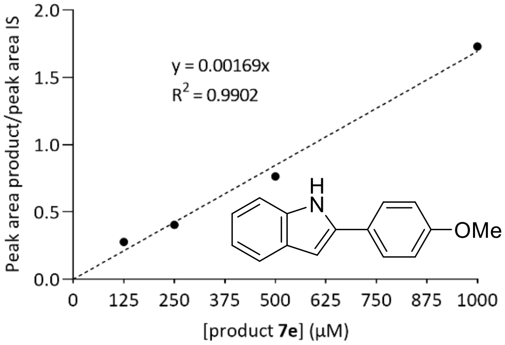

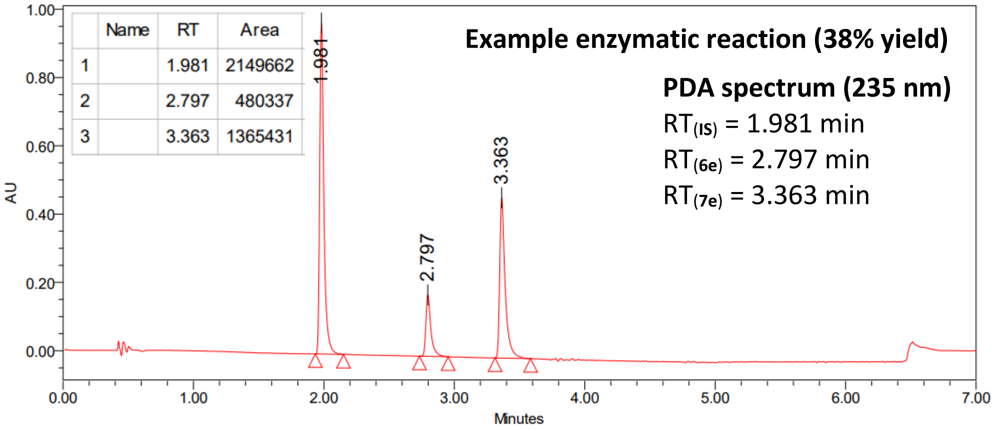

SI.31 References supporting information

[1] S. Rajagopalan, G. Radke, M. Evans, J. M. Tomich, *Synth. Commun.* **1996**, *26*, 1431–1440.

[2] J. D. Rudolf, C. D. Poulter, *ACS Chem. Biol.* **2013**, *8*, 2707–2714.

[3] I. Drienovská, C. Mayer, C. Dulson, G. Roelfes, *Nat. Chem.* **2018**, *10*, 946–952.

[4] M. T. Weinstock, E. D. Hesek, C. M. Wilson, D. G. Gibson, *Nat. Methods* **2016**, *13*, 849–851.

[5] S. S. González, O. Ad, B. Shah, Z. Zhang, X. Zhang, A. Chatterjee, A. Schepartz, *ACS Cent. Sci.* **2021**, *7*, 1500–1507.

[6] C. Lu, X. Peng, B. Maity, X. Sheng, Y. Zhou, T. Ueno, Z. Liu, D. Lu, *ACS Catal.* **2023**, *13*, 9918–9924.

[7] M. Amiram, A. D. Haimovich, C. Fan, Y.-S. Wang, H.-R. Aerni, I. Ntai, D. W. Moonan, N. J. Ma, A. J. Rovner, S. H. Hong, N. L. Kelleher, A. L. Goodman, M. C. Jewett, D. Söll, J. Rinehart, F. J. Isaacs, *Nat. Biotechnol.* **2015**, *33*, 1272–1279.

[8] S. J. Miyake-Stoner, A. M. Miller, J. T. Hammill, J. C. Peeler, K. R. Hess, R. A. Mehl, S. H. Brewer, *Biochemistry* **2009**, *48*, 5953–5962.

[9] K. C. Schultz, L. Supekova, Y. Ryu, J. Xie, R. Perera, P. G. Schultz, *J. Am. Chem. Soc.* **2006**, *128*, 13984–13985.

[10] R. A. Mehl, J. C. Anderson, S. W. Santoro, L. Wang, A. B. Martin, D. S. King, D. M. Horn, P. G. Schultz, *J. Am. Chem. Soc.* **2003**, *125*, 935–939.

[11] A. Deiters, D. Groff, Y. Ryu, J. Xie, P. G. Schultz, *Angew. Chem. Int. Ed.* **2006**, *45*, 2728–2731.

[12] J. W. Chin, A. B. Martin, D. S. King, L. Wang, P. G. Schultz, *Proc. Natl. Acad. Sci.* **2002**, *99*, 11020–11024.

[13] J. W. Chin, S. W. Santoro, A. B. Martin, D. S. King, L. Wang, P. G. Schultz, *J. Am. Chem. Soc.* **2002**, *124*, 9026–9027.

[14] M. R. Seyedsayamdost, J. Xie, C. T. Y. Chan, P. G. Schultz, J. Stubbe, *J. Am. Chem. Soc.* **2007**, *129*, 15060–15071.

[15] J. Xie, W. Liu, P. G. Schultz, *Angew. Chem. Int. Ed.* **2007**, *46*, 9239–9242.

[16] H. Ai, W. Shen, A. Sagi, P. R. Chen, P. G. Schultz, *ChemBioChem* **2011**, *12*, 1854–1857.

[17] T. Plass, S. Milles, C. Koehler, C. Schultz, E. A. Lemke, *Angew. Chem. Int. Ed.* **2011**, *50*, 3878–3881.

[18] R. Rubini, I. Ivanov, C. Mayer, *Chem. Eur. J.* **2019**, *25*, 16017–16021.

[19] J. Bos, W. R. Browne, A. J. M. Driessen, G. Roelfes, *J. Am. Chem. Soc.* **2015**, *137*, 9796–9799.

[20] R. B. Leveson-Gower, Z. Zhou, I. Drienovská, G. Roelfes, *ACS Catal.* **2021**, *11*, 6763–6770.

[21] E. M. Gabor, E. J. De Vries, D. B. Janssen, *Environ. Microbiol.* **2004**, *6*, 948–958.

[22] D. J. Weber, A. G. Gittis, G. P. Mullen, C. Abeygunawardana, E. E. Lattman, A. S. Mildvan, *Proteins* **1992**, *13*, 275–287.

[23] R. B. Leveson-Gower, R. M. de Boer, G. Roelfes, *ChemCatChem* **2022**, *14*, e202101875.

[24] L. Longwitz, R. B. Leveson-Gower, H. J. Rozeboom, A.-M. W. H. Thunnissen, G. Roelfes, *Nature* **2024**, *629*, 824–829.

[25] H. Inoue, H. Nojima, H. Okayama, *Gene* **1990**, *96*, 23–28.

[26] K. M. Sparta, M. Krug, U. Heinemann, U. Mueller, M. S. Weiss, *J. Appl. Crystallogr.* **2016**, *49*, 1085–1092.

[27] P. R. Evans, G. N. Murshudov, *Acta Crystallogr. D* **2013**, *69*, 1204–1214.

[28] M. D. Winn, C. C. Ballard, K. D. Cowtan, E. J. Dodson, P. Emsley, P. R. Evans, R. M. Keegan, E. B. Krissinel, A. G. W. Leslie, A. McCoy, S. J. McNicholas, G. N. Murshudov, N. S. Pannu, E. A. Potterton, H. R. Powell, R. J. Read, A. Vagin, K. S. Wilson, *Acta Crystallogr. D* **2011**, *67*, 235–242.

[29] A. J. McCoy, R. W. Grosse-Kunstleve, P. D. Adams, M. D. Winn, L. C. Storoni, R. J. Read, *J. Appl. Crystallogr.* **2007**, *40*, 658–674.

[30] C. Mayer, C. Dulson, E. Reddem, A.-M. W. H. Thunnissen, G. Roelfes, *Angew. Chem. Int. Ed.* **2019**, *58*, 2083–2087.

[31] P. Emsley, K. Cowtan, *Acta Crystallogr. D* **2004**, *60*, 2126–2132.

[32] G. N. Murshudov, P. Skubák, A. A. Lebedev, N. S. Pannu, R. A. Steiner, R. A. Nicholls, M. D. Winn, F. Long, A. A. Vagin, *Acta Crystallogr. D* **2011**, *67*, 355–367.

[33] F. Long, R. A. Nicholls, P. Emsley, S. Gražulis, A. Merkys, A. Vaitkus, G. N. Murshudov, *Acta Crystallogr. D* **2017**, *73*, 112–122.
[34] V. B. Chen, I. I. I. W. Bryan Arendall, J. J. Headd, D. A. Keedy, R. M. Immormino, G. J. Kapral, L. W. Murray, J. S. Richardson, D. C. Richardson, *Acta Crystallogr. D* **2010**, *66*, 12.
[35] E. Kumaran, W. Y. Fan, W. K. Leong, *Org. Lett.* **2014**, *16*, 1342–1345.
[36] S. Dhanasekaran, Vinod. K. Kannaujiya, R. G. Biswas, V. K. Singh, *J. Org. Chem.* **2019**, *84*, 3275–3292.
[37] A. Ikeda, M. Omote, K. Kusumoto, M. Komori, A. Tarui, K. Sato, A. Ando, *Org. Biomol. Chem.* **2016**, *14*, 2127–2133.
[38] C. Peng, Y. Wang, L. Liu, H. Wang, J. Zhao, Q. Zhu, *Eur. J. Org. Chem.* **2010**, *2010*, 818–822.
[39] K. Punjajom, J. Tummatorn, S. Ruchirawat, C. Thongsornkleeb, *Asian J. Org. Chem.* **2021**, *10*, 906–917.
[40] F. Christoffel, N. V. Igareta, M. M. Pellizzoni, L. Tiessler-Sala, B. Lozhkin, D. C. Spiess, A. Lledós, J.-D. Maréchal, R. L. Peterson, T. R. Ward, *Nat. Catal.* **2021**, *4*, 643–653.
